# Identification and development of a novel invasion-related gene signature for prognosis prediction in colon adenocarcinoma

**DOI:** 10.1186/s12935-021-01795-1

**Published:** 2021-02-12

**Authors:** Jiahua Liu, Chunhui Jiang, Chunjie Xu, Dongyang Wang, Yuguang Shen, Ye Liu, Lei Gu

**Affiliations:** Department of Gastrointestinal Surgery, Renji Hospital, School of Medicine, Shanghai Jiao Tong University, Shanghai, China

**Keywords:** Colon carcinoma, Prognosis, Molecular subtyping, Gene signature, Invasion-related genes

## Abstract

The overall survival of metastatic colon adenocarcinoma (COAD) remains poor, so it is important to explore the mechanisms of metastasis and invasion. This study aimed to identify invasion-related genetic markers for prognosis prediction in patients with COAD. Three molecular subtypes (C1, C2, and C3) were obtained based on 97 metastasis-related genes in 365 COAD samples from The Cancer Genome Atlas (TCGA). A total of 983 differentially expressed genes (DEGs) were identified among the different subtypes by using the limma package. A 6-gene signature (*ITLN1*, *HOXD9*, *TSPAN11*, *GPRC5B*, *TIMP1*, and *CXCL13*) was constructed via Lasso-Cox analysis. The signature showed strong robustness and could be used in the training, testing, and external validation (GSE17537) cohorts with stable predictive efficiency. Compared with other published signatures, our model showed better performance in predicting outcomes. Pan-cancer expression analysis results showed that *ITLN1*, *TSPAN11*, *CXCL13*, and *GPRC5B* were downregulated and *TIMP1* was upregulated in most tumor samples, including COAD, which was consistent with the results of the TCGA and GEO cohorts. Western blot analysis and immunohistochemistry were performed to validate protein expression. Tumor immune infiltration analysis results showed that TSPAN11, GPRC5B, TIMP1, and CXCL13 protein levels were significantly positively correlated with CD4+ T cells, macrophages, neutrophils, and dendritic cells. Further, the TIMP1 and CXCL13 proteins were significantly related to the tumor immune infiltration of CD8+ T cells. We recommend using our signature as a molecular prognostic classifier to assess the prognostic risk of patients with COAD.

## Introduction

Colorectal cancer (CRC) is the third most commonly diagnosed malignancy and the fourth leading cause of cancer-related death worldwide, with more than 2.2 million new cases and 1.1 million related deaths predicted by 2030 [1]. About 20% of CRC patients have metastatic disease when first diagnosed, and about 30–50% of patients with primary colon cancer relapse and die from metastatic cancer [2, 3]. Surgery is the main treatment for colon cancer, and the 5-year survival rate is 50% [4]. Tumor recurrence after radical surgery is the main obstacle when it comes to improving overall survival. Diagnoses of advanced colon cancer, the paucity of safe chemotherapy drugs, and the lack of effective therapeutic targets are serious obstacles in the treatment of colon cancer.

Metastasis is a major cause of death in cancer patients. Metastasis requires cancer cells to leave the primary tumor and acquire the ability to migrate and invade. Understanding the mechanisms involved in cell invasion and migration in complex environments is a critical step in successful anti-metastasis therapy. The process of epithelial-mesenchymal transition has been found to mediate cell-to-cell adhesion, regulate cell-to-matrix adhesion, and induce proteolytic enzyme secretion in the extracellular matrix [5]. Further, the S100P protein has been found to promote CRC invasion and metastasis by activating RAGE/ERK signaling and promoting the epithelial-mesenchymal transition process [6]. In addition, Hecht et al. focused on the basic principles of metastatic aggression, including motion, invasion, proliferation, and metabolism, by using experimental data-based modeling [7].

Due to the poor prognosis of colon cancer, it is necessary to identify prognostic biomarkers to determine molecular changes in patients with colon cancer, which will ultimately facilitate appropriate individualized treatments for patients with a high risk of recurrence. The tumor-node-metastasis staging system is the gold standard for predicting prognosis in patients with CRC [8]. The detection of blood-based tumor markers has been accepted as a potential non-invasive alternative method to detect cancer, but the incidence rates of false-positives and false-negatives are high [9]. Clinical markers, such as poor differentiation, vascular invasion, and/or nerve invasion, and molecular markers, such as microsatellite instability status, *KRAS* mutation status, *NRAS* mutation status, and *BRAF* mutation status, can also be used [10–12]. Furthermore, recent studies have emphasized the prognostic value of immune infiltration [13, 14]. Therefore, there is an urgent need to identify highly robust biomarkers to enable individualized treatment decisions, which may then guide drug development and the use of combination therapies, targeted therapies, and immunotherapies. With the development of genomic technology, many epigenetic changes have been identified as potential clinical biomarkers in CRC patients, particularly in terms of aberrant DNA methylation processes, microRNA and noncoding RNA disorders, and histone modification changes [15, 16]. However, genetic changes are still important factors in the development of colon cancer. Therefore, finding prognostic markers is critical for better patient management.

In this study, invasion-related genes were selected, and colon adenocarcinoma (COAD) subtypes based on tumor invasion-related genes were identified by using gene expression data from the public databases of The Cancer Genome Atlas (TCGA) and Gene Expression Omnibus (GEO). The molecular subtypes’ relationships with prognosis and clinical features were evaluated. A prognostic risk model was constructed by using differentially expressed genes (DEGs) among the molecular subtypes of COAD, and it could effectively predict prognosis in COAD samples. A 6-gene signature was developed by using the robust likelihood-based survival model. We verified the good performance of the prognostic risk model by using the GEO gene expression cohort. Finally, gene set enrichment analysis (GSEA) was used to study the biological and functional enrichment features of the 6-gene signature. Our 6-gene signature may be useful in the classification of colon cancer patients with different prognoses, and some of the genes in the signature may represent new therapeutic targets.

## Materials and methods

### Data download and preprocessing

The latest RNA sequencing expression profiles and clinical follow-up information of COAD samples were downloaded from the TCGA database. The following steps were performed for the RNA sequencing data of the TCGA-COAD cohort:

1. Samples with the pathological type of “Colon Adenocarcinoma” were selected for analysis;
2. Samples without clinical follow-up information were removed;
3. Samples with a survival time of < 30 days were removed;
4. Samples without survival status information were removed;
5. Ensembl was converted to Gene Symbol; and
6. The median value of the expression levels of multiple Gene Symbols was obtained.

GEO data were downloaded from the GEO database, and the GSE17537 chip data set with survival time was selected. The GEO data were preprocessed as follows:

1. Samples without clinical follow-up information were removed;
2. Samples without survival time and survival status information were removed;
3. Probe was transferred to Gene Symbol;
4. Probes corresponding to multiple genes were removed; and
5. The median value of the expression levels of multiple Gene Symbols was obtained.

There were 365 samples in the TCGA-COAD cohort and 54 COAD samples in the GSE17537 cohort after preprocessing. The clinical features of the samples are given in Table 1.

**Table 1:** Sample clinical features

Clinical features	TCGA-COAD	GSE17537
OS		
0	287	35
1	78	19
T stage		
T1	9	
T2	65	
T3	251	
T4	39	
TX	1	
N stage		
N0	214	
N1	89	
NX	62	
M stage		
M0	270	
M1	54	
MX	41	
Stage		
I	61	4
II	138	14
III	101	19
IV	54	17
X	11	
Gender		
Male	201	25
Female	164	29
Age		
≤ 65	153	33
> 65	212	21

### Identification of molecular subtypes based on invasion-related genes and functional enrichment analysis

A gene set containing 97 invasion-related genes was obtained from the CancerSEA database (Additional file 1: Table S1).

The expression profiles of these 97 invasion-related genes were extracted from the TCGA database, and they were analyzed by using the coxph function for univariate Cox analysis. Next, ConsensusClusterPlus (parameters: reps = 100, pItem = 0.8, pFeature = 1, distance = “Canberra”) was used to obtain molecular subtypes of colon cancer by consensus clustering of the significant genes gathered from the univariate Cox analysis. DEGs between the different molecular subtypes were identified by using the limma package. Kyoto Encyclopedia of Genes and Genomes (KEGG) pathway analysis and Gene Ontology (GO) functional enrichment analysis were performed on the DEGs between the molecular subtypes with the R software package WebGestaltR (v0.4.2).

### Construction of a prognostic risk model based on invasion-related genes

The 365 samples from the TCGA database were divided into training and validation cohorts. To prevent random allocation bias from affecting the stability of subsequent modeling, all samples were randomly grouped with replacement 200 times in advance. Group sampling was based on a training cohort-to-verification cohort ratio of 3:2. For DEGs between molecular subtypes and survival data in the training cohort, the coxph function of the survival package in R was applied to the univariate Cox proportional hazards regression model, and a P-value of < 0.05 was the threshold for filtering.

### Multivariate analysis in the training cohort

We used the R software package glmnet to perform least absolute shrinkage and selection operator (Lasso) analysis per Tibshirani (1996) and Cox regression analysis on the significant genes from the univariate analysis in order to reduce the number of genes in the risk model. The Lasso method is a compression estimate. It can be used to construct a penalty function to obtain a more refined model, compressing some coefficients and setting others to 0. It therefore retains the advantage of subset contraction and is a kind of biased estimation for processing multicollinearity data. This method can be used to realize the selection of variables while estimating parameters and better solve the problem of multicollinearity that is present in regression analysis.

The stepwise regression used the Akaike information criterion (AIC), which is a measure of the statistical goodness of fit and penalized for the number of parameters. The step method in the stats package starts from the most complex model and removes single variables in turn to decelerate the AIC. The smaller the value, the more superior the model. The model therefore has better fit with fewer parameters.

### Risk score and pathway relationships

To observe the relationships between risk score and biological functions in different samples, we selected the gene expression profiles corresponding to these samples and performed single-sample gene set enrichment analysis (ssGSEA) with the R software package GSVA. By calculating the score of each sample for different functions, that is, by obtaining each sample’s ssGSEA score corresponding to each function, we calculated the correlations between the functions and risk score.

### Construction of a nomogram based on clinical features

A nomogram can be used to predict the outcome of a risk model intuitively and effectively, and it is convenient to apply. The lengths of the lines in the nomogram indicate the degrees to which different variables influence the outcome and the effects of different variable values on the outcome. Furthermore, the calibration curve was used to estimate the prediction accuracy of the model. A calibration curve that is close to the standard curve indicates that the model has good predictive performance. We also used decision curve analysis (DCA) to evaluate the clinical utility of the model.

### Clinical expression of genes in the Oncomine and GEO cohorts

Oncomine (http://www.oncomine.org) is a gene chip-based database and integrated data-mining platform. In this study, we set the screening criteria as follows: (1) cancer type: COAD; (2) analysis type: Cancer vs Normal Analysis; and (3) threshold criteria: P < 0.05, fold change > 1.5, and gene rank = top 10%. The COAD cohort was downloaded from the GEO and TCGA databases. The ggplot2 and ggpubr R packages were used to visualize the expression levels of 6 genes in the colon cancer data set.

### Correlations between gene expression levels in pan-carcinoma and immune infiltrating cells

We downloaded the scores of 6 immune infiltrating cells from 33 cancers from the TIMER database (https://cistrome.shinyapps.io/timer/), and we analyzed the correlations between gene expression levels and immune cell scores by using Spearman’s method. We also used the TIMER database to analyze the expression levels of 6 genes in 33 cancer tissues.

### Immunohistochemistry and protein expression validation

The Human Protein Atlas (HPA) provides information on the tissue and cell distributions of 26,000 human proteins. We explored the expression levels of the single bar proteins of 6 genes (*ITLN1*, *HOXD9*, *TSPAN11*, *GPRC5B*, *TIMP1*, and *CXCL13*) in normal colon tissues and tumor tissues.

### Sample collection

The Ethics Committee of Renji Hospital Affiliated with Shanghai Jiaotong University approved this study. COAD tissues and adjacent normal tissues were collected from 3 patients, placed in liquid nitrogen immediately, and preserved at − 80 °C. The included patients and their families were fully informed, and the participants provided informed consent.

### Western blot analysis

Western blot analysis was carried out according to standard protocols. We used primary antibodies raised against GAPDH, CXCL13, ITLN1, HOXD9, and GPRC5B (Santa-Cruz Biotechnology, CA, USA), as well as TIMP1 and TSPAN11 (Proteintech, China). Goat anti-mouse and anti-rabbit antibodies conjugated with horseradish peroxidase were used as secondary antibodies (Jackson ImmunoResearch, PA, USA). We detected the blots by using enhanced chemiluminescence (Dura, Pierce, NJ, USA).

### RNA extraction and real-time polymerase chain reaction assay

Total RNA was extracted by using TRIzol Reagent (Invitrogen, CA, USA) following the manufacturer’s protocol, and it was reverse transcribed into complementary DNA (cDNA) by using a Superscript Reverse Transcriptase Kit (Transgene, France). A Super SYBR Green Kit (Transgene, France) was used to carry out real-time polymerase chain reaction in an ABI7300 real-time polymerase chain reaction system (Applied Biosystems). The primer pairs were: CXCL13 forward: GCTTGAGGTGTAGATGTGTCC, CXCL13 reverse: CCCACGGGGCAAGATTTGAA; ITLN1 forward: ACGTGCCCAATAAGTCCCC, ITLN1 reverse: CCGTTGTCAGTCCAACACTTTC; TIMP1 forward: CTTCTGCAATTCCGACCTCGT, TIMP1 reverse: ACGCTGGTATAAGGTGGTCTG; TSPAN11 forward: CATCTTTGCGGGCGTACTTG, TSPAN11 reverse: CAGGCAGAAATACGTGGAGAG; HOXD9 forward: GGACTCGCTTATAGGCCATGA, HOXD9 reverse: GCAAAACTACACGAGGCGAA; and GPRC5B forward: CCTCCTCCCTCAGTACGTGTC, GPRC5B reverse: AAGGCAAACGTCAGCCCAAA.

## Results

### Identification of molecular subtypes based on NMF

**Fig. 1:**
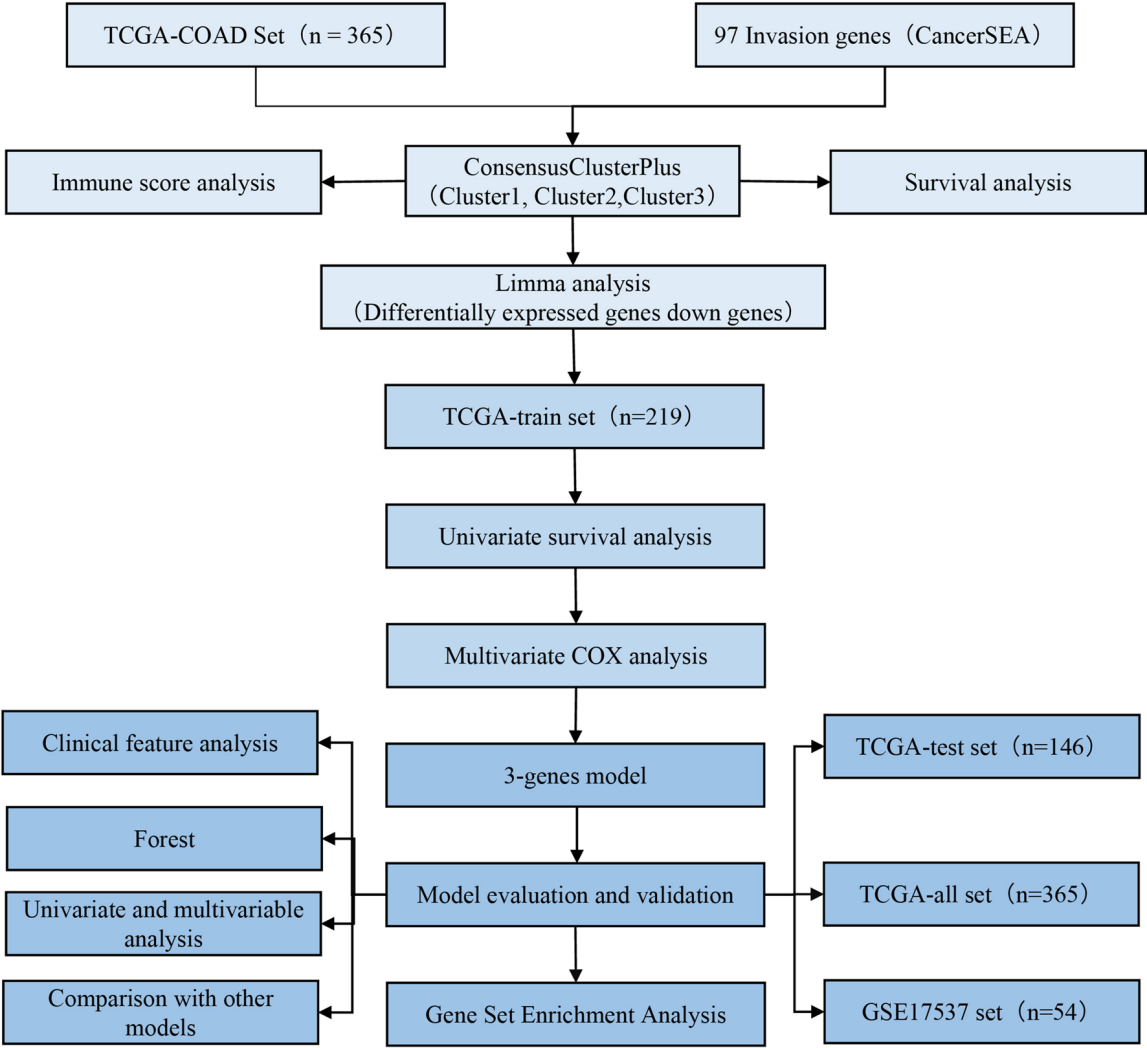
The protocol of colon carcinoma invasion-related prognosis features

**Fig. 2:**
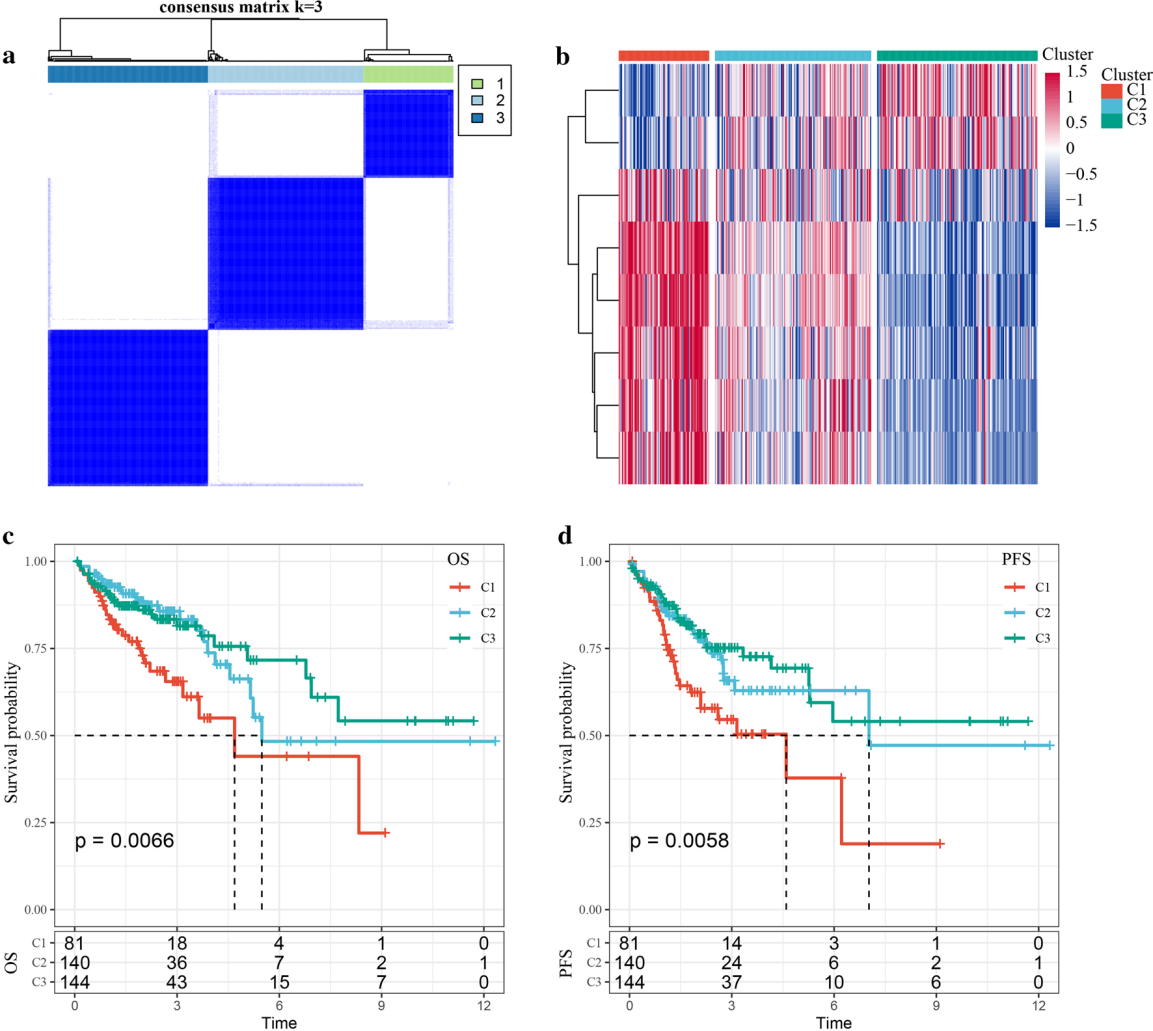
**a** Samples cluster heatmap with consistent cluster k = 3; **b** cluster heatmap of 8 prognostic genes; **c** OS survival curves of all TCGA colon carcinoma samples molecular subtypes; **d** PFS survival curves of all TCGA colon carcinoma samples molecular subtypes

We designed a protocol (Fig. 1) to analyze the invasion-related genes associated with the prognosis of colon carcinoma. Eight genes (Additional file 2: Table S2) were found to be associated with the prognosis of colon carcinoma (P < 0.05). Next, ConsensusClusterPlus was used to identify molecular subtypes based on these genes. The cluster was stable when k was equal to 3 (Fig. 2a). The expression levels of prognostic invasion-related genes in the 3 subtypes (Cluster 1 [C1], Cluster 2 [C2], and Cluster 3 [C3]) were plotted (Fig. 2b). The gene expression levels in C1, C2, and C3 were different, and most of the genes were highly expressed in the C1 subtype and lowly expressed in the C3 subtype. Furthermore, prognoses among the 3 subtypes were significantly different. Patients with the C1 subtype showed the worst prognosis, and patients with the C3 subtype showed the best prognosis (log-rank P < 0.05; Fig. 2c, d).

### Identification of differentially expressed genes

**Fig. 3:**
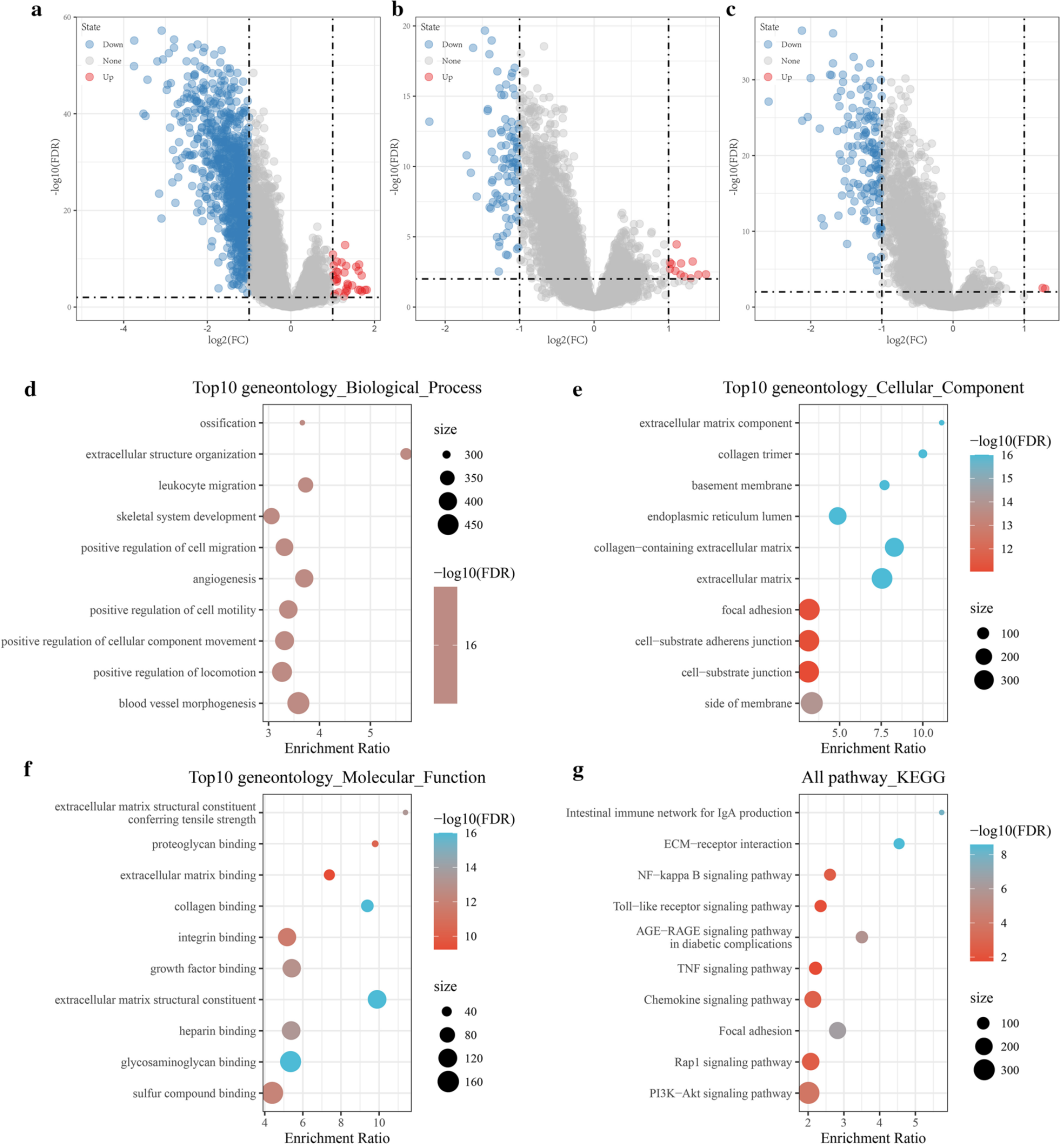
**a** Volcano map of C1 and C3 subtypes DEGs; **b** Volcano map of C1 and C2 subtypes DEGs; **c** Volcano map of C2 and C3 subtypes DEGs; **d** The significant top 10 GO functional annotations in biological process; **e** The significant top 10 GO functional annotations in cellular component; **f** M The significant top 10 GO functional annotations in molecular function; **g** the top 10 KEGG pathway of DEGs

The DEGs between C1 and C3, C2 and C3, and C1 and C2 were obtained by using the limma package, with a false discovery rate of < 0.05 and |log2FC| value of > 1 as the threshold. As shown in Fig. 3a, there were 38 upregulated and 942 downregulated genes between C1 and C3, and the genes were predominantly downregulated (Additional file 3: Table S3); as shown in Fig. 3b, there were 17 upregulated and 96 downregulated genes between C1 and C2, and the genes were predominantly downregulated (Additional file 4: Table S4); as shown in Fig. 3c, there were 2 upregulated and 150 downregulated genes between C2 and C3, and the DEGs are shown in Additional file 5: Table S5.

Further, we used the R software package WebGestaltR (v0.4.2) to perform KEGG pathway analysis and GO functional enrichment analysis of the 983 DEGs between C1 and C3, C1 and C2, and C2 and C3.

The 10 most significant GO functional annotations in biological processes (Fig. 3d), cellular components (Fig. 3e), and molecular functions (Fig. 3f) were visualized.

The top 10 KEGG pathways of the DEGs showed that most DEGs were significantly enriched in tumor pathogenesis signaling pathways, such as the intestinal immune network for IgA production, extracellular matrix-receptor interactions, the AGE-RAGE signaling pathway in diabetic complications, focal adhesions, and the PI3K-Akt signaling pathway (Fig. 3g).

### Comparisons of immune scores between molecular subtypes

**Fig. 4:**
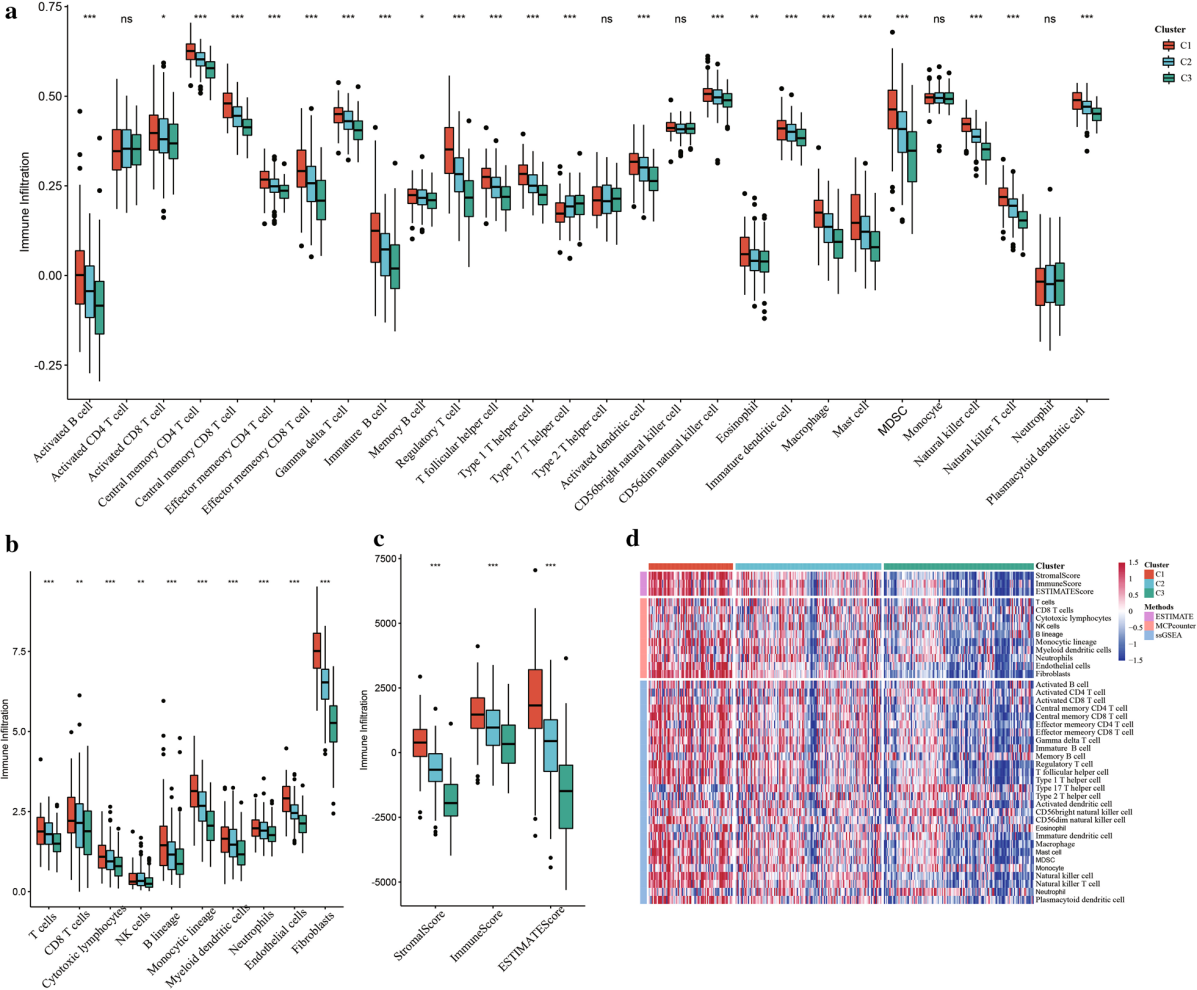
**a** Comparison of ssGSEA Immune score among molecular subtypes in TCGA dataset; **b** Comparison of Mcpounte Immune score among molecular subtypes in TCGA dataset; **c** Comparison of Estimate Immune score among molecular subtypes in TCGA dataset; **d** Comparison of different immune immune scores among molecular subtypes in TCGA dataset

To determine the relationships between immune scores and molecular subtypes, the ESTIMATE package was used to evaluate stromal scores, immune scores, and ESTIMATE scores. MCPcounter was used to assess the scores of 10 immune cells, and the GSCA package for ssGSEA was used to calculate the scores of 28 immune cells. The results of comparisons of immune scores between molecular subtypes showed that the immune scores of C1 in the 3 software programs were higher than those of C2 and C3 (Fig. 4a–c). We also plotted a heat map of the immune scores of the 3 subtypes (Fig. 4d).

### Comparisons of immune molecular subtypes

**Fig. 5:**
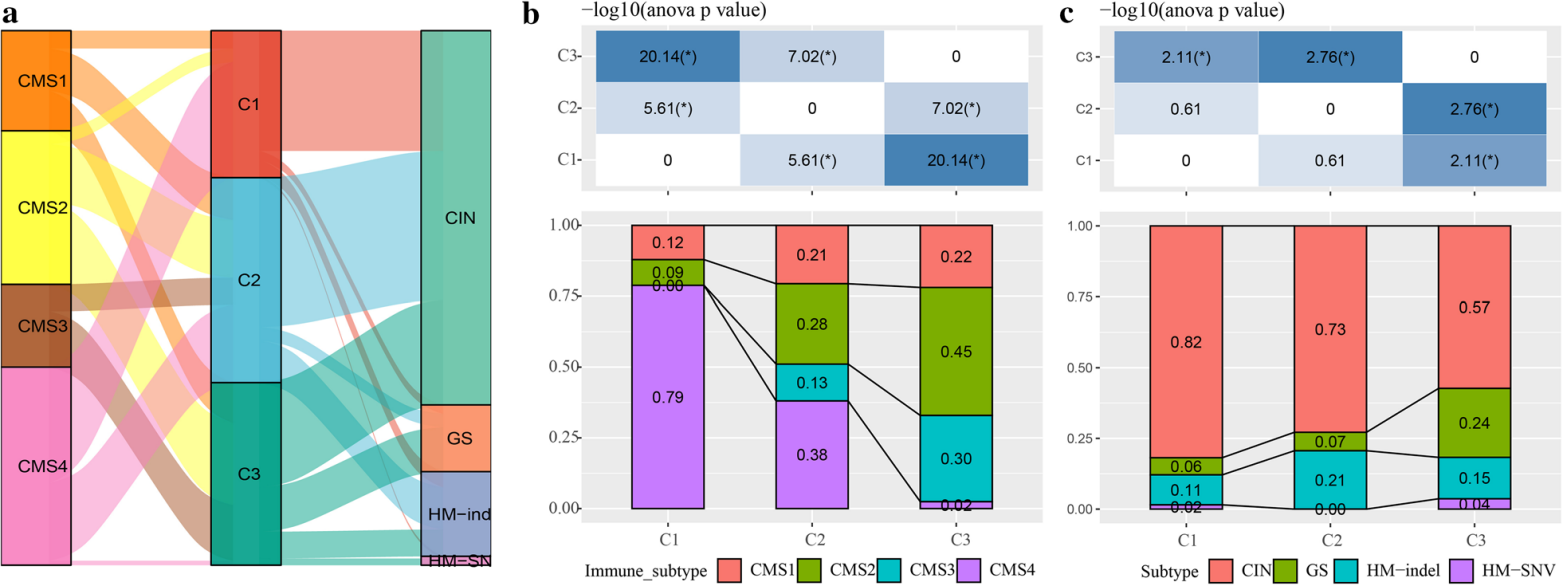
**a** Sankey map between our molecular subtype and existing subtypes; **b** Distribution comparison of CMS subtypes between different molecular subtype; **c** Distribution comparison of Thorsson subtypes between different molecular subtype

There are 4 consensus molecular subtypes of colon cancer: CMS1 (MSI-immune), CMS2 (canonical), CMS3 (metabmal), and CMS4 (mesenchymal). Most of the colon cancer patients in the TCGA cohort were CMS2 and CMS4 (about 66.26%). In addition, we obtained 4 subtypes of TCGA colon cancer from the literature [17]: CIN, GS, HM-indel, and HM-SNV. A Sankey diagram was used to show the distribution of relationships between them (Fig. 5a).

In the C1 subtype, the proportion of the CMS4 subtype was significantly higher than that in the C2 and C3 subtypes, and the proportion of the CMS1 subtype was significantly less than that in the C2 and C3 subtypes (Fig. 5b). In addition, in the C1 subtype, the proportion of the CIN subtype was significantly higher than that in the C3 subtype, and the proportion of the GS subtype in the C3 subtype was higher than that in the C1 and C2 subtypes (Fig. 5c).

### Construction of a prognostic risk model based on invasion-related genes

**Fig. 6:**
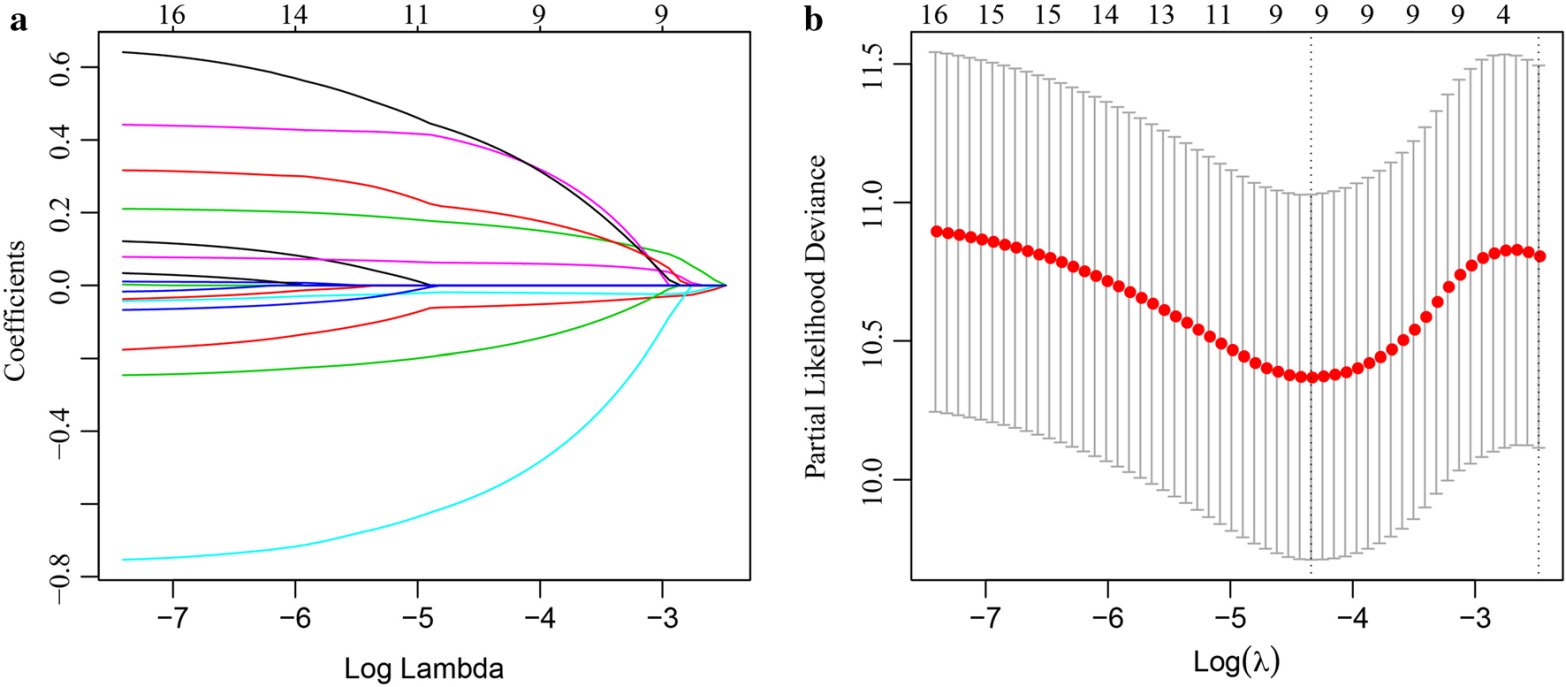
**a** The changing trajectory of each independent variable. The horizontal axis represents the log value of the independent variable lambda, and the vertical axis represents the coefficient of the independent variable. **b** The confidence interval under each lambda

The 365 samples in the TCGA data set were divided into training and verification cohorts (219 and 146 samples, respectively) according to the ratio of 3:2. For the training cohort, 983 DEGs between the molecular subtypes were analyzed by univariate Cox analysis, and P < 0.05 was selected as the threshold for filtering. We obtained 17 genes related to prognosis (Additional file 6: Table S6). The R package glmnet was used to perform Lasso-Cox regression. First, the change trajectory of each independent variable was analyzed (Fig. 6a). The results showed that the number of independent variable coefficients approaching 0 increased with the gradual increase of lambda. A tenfold cross-validation was applied to construct the model, and the confidence interval of each lambda is shown in Fig. 6b. Nine genes (*ITLN1*, *AKR1B10*, *FABP4*, *HOXD9*, *TSPAN11*, *GPRC5B*, *TIMP1*, *SNAI1*, and *CXCL13*) were selected as target genes for the next analysis when the model reached the optimal value, with lambda equal to 0.01429895.

Furthermore, the stepwise regression algorithm was used to reduce the 9 genes to 6: *ITLN1*, *HOXD9*, *TSPAN11*, *GPRC5B*, *TIMP1*, and *CXCL13*.

The final 6-gene signature formula was: RiskScore = − 0.0918378 * ITLN1 + 0.2165212 * HOXD9 − 0.7173256 * TSPAN11 + 0.6454939 * GPRC5B + 0.6350192 * TIMP1 − 0.2074915 * CXCL13.

### Construction and verification of risk model

**Fig. 7:**
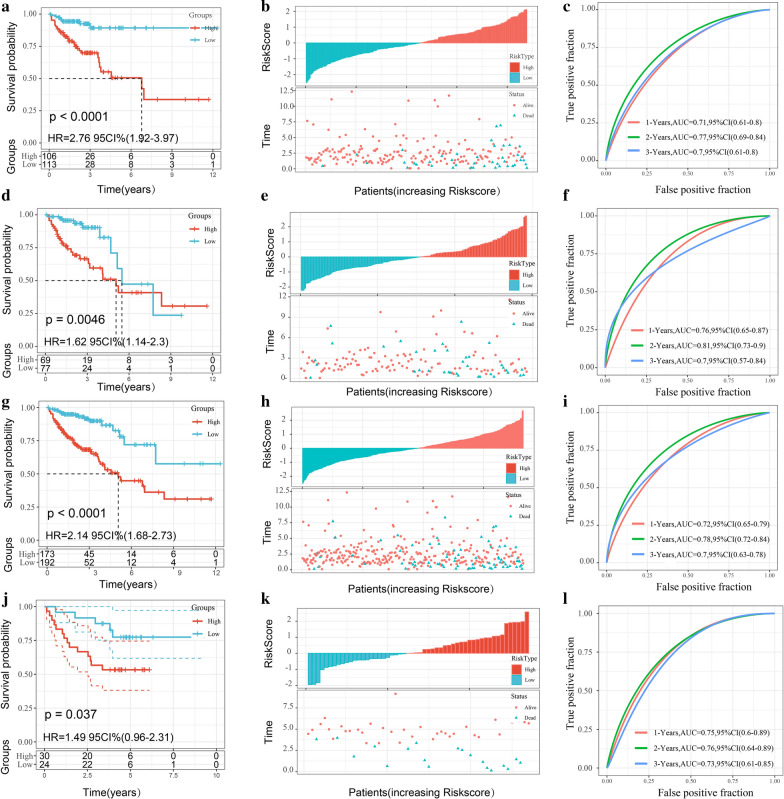
**a** Survival curves between two risk groups based on 6-gene signature classification; **b** distribution of RiskScore and survival status of 6-gene signature in TCGA training cohort; **c** ROC curve of 6-gene signature classification in TCGA training cohort; **d**–**f** survival curves between two risk groups, distribution of RiskScore and survival status, ROC curve of 6-gene signature in TCGA testing cohort; **g**–**i** survival curves between two risk groups, distribution of RiskScore and survival status, ROC curve of 6-gene signature in entire TCGA cohort; **j**–**l** survival curves between two risk groups, distribution of RiskScore and survival status, ROC curve of 6-gene signature in GSE17537cohort

The risk score of each sample of the training cohort was calculated according to expression levels. The risk score distribution is shown in Fig. 7b. The overall survival rates of the samples with high risk scores were significantly lower than those of samples with low risk scores, suggesting worse prognoses in samples with high risk scores. Furthermore, receiver operating characteristic (ROC) analysis was conducted by using the R software package timeROC. We analyzed the classification efficiency of prognosis prediction (Fig. 7c). The average areas under the curve (AUCs) of 1, 2, and 3 years reached 0.71, 0.77, and 0.70, respectively. Finally, the Z-score method was applied in the preprocessing of the risk scores, and samples with risk scores > 0 were divided into the high-risk group (106 samples), while samples with risk scores < 0 were divided into the low-risk group (113 samples). The Kaplan–Meier curve showed that there was a significant difference between the 2 risk groups (P < 0.0001; Fig. 7a).

To determine the robustness of the model, we used the same model and the same coefficient as the training cohort in the different cohorts. We calculated the risk scores of the samples according to the expression levels of each sample and plotted the risk score distributions of the samples.

The risk score distributions of the TCGA testing cohort, the entire TCGA cohort, and the independent verification cohort (GSE17537) are shown in Fig. 7e, h, k. In the testing cohort, the predictive efficiency of 1, 2, and 3 years reached 0.76, 0.81, and 0.70, respectively (Fig. 7f). The prognosis of the high-risk group (69 samples) was significantly worse than that of the low-risk group (77 samples) (P = 0.0046; Fig. 7d).

Similarly, in the entire TCGA cohort, the predictive efficiency of 1, 2, and 3 years reached 0.72, 0.78, and 0.70, respectively (Fig. 7i). There was a significant difference between the high-risk group (173 samples) and low-risk group (192 samples) (P < 0.0001; Fig. 7g).

In the independent verification cohort (GSE17537), the predictive efficiency of 1, 2, and 3 years reached 0.75, 0.76, and 0.30, respectively (Fig. 7l). There was a significant difference between the high-risk group (30 samples) and low-risk group (24 samples) (P = 0.037; Fig. 7j).

### Prognostic analysis of risk model and clinical features

**Fig. 8:**
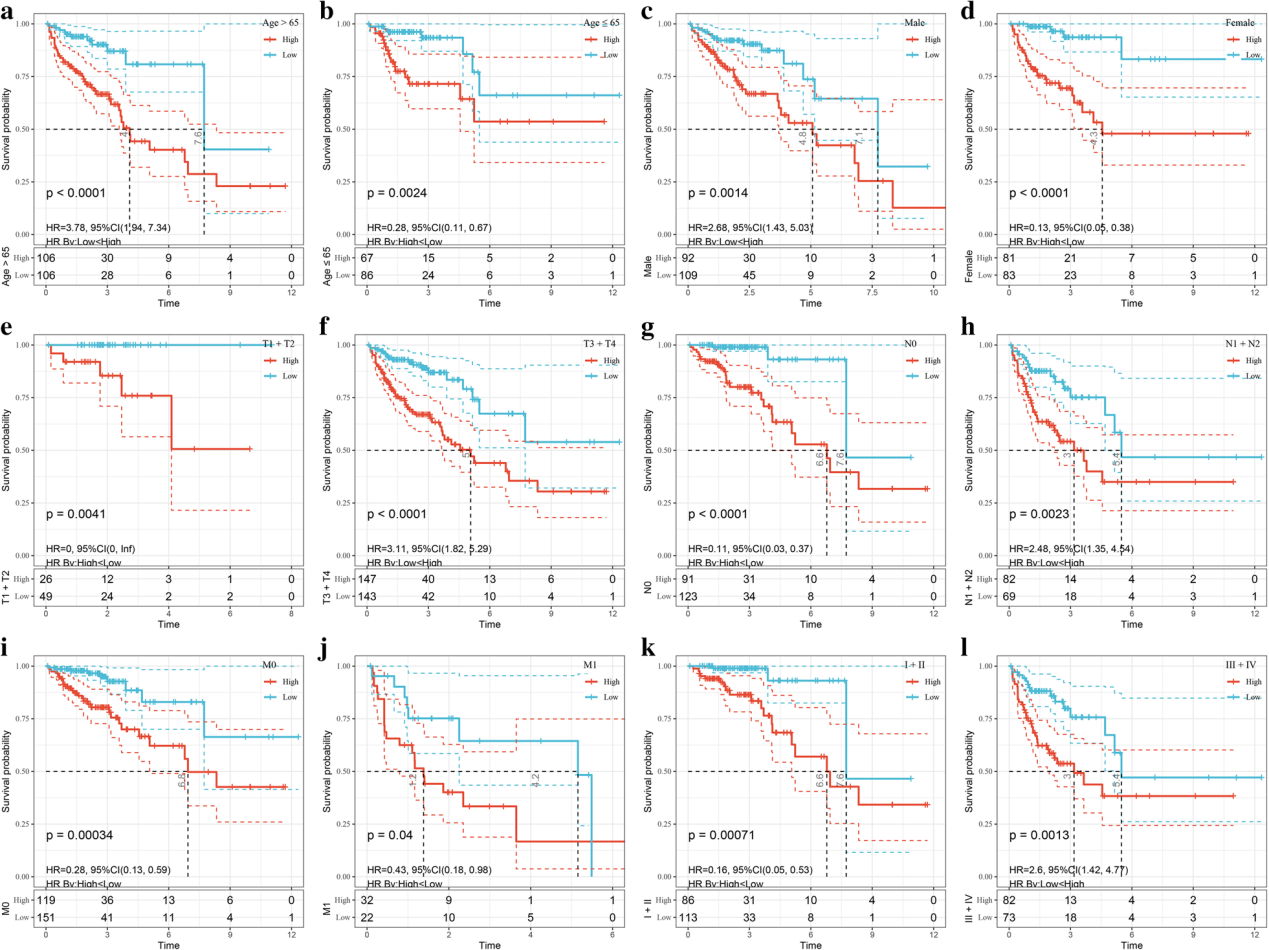
The prognostic performance of the risk model in different clinical features

Based on risk score, age, sex, T stage, N stage, M stage, and clinical stage could be grouped into high- and low-risk groups, and there were significant prognostic differences (P < 0.05; Fig. 8). This result further suggested that the risk score model had good predictive ability in terms of different clinical features.

### Correlations between risk score and clinical features

**Fig. 9:**
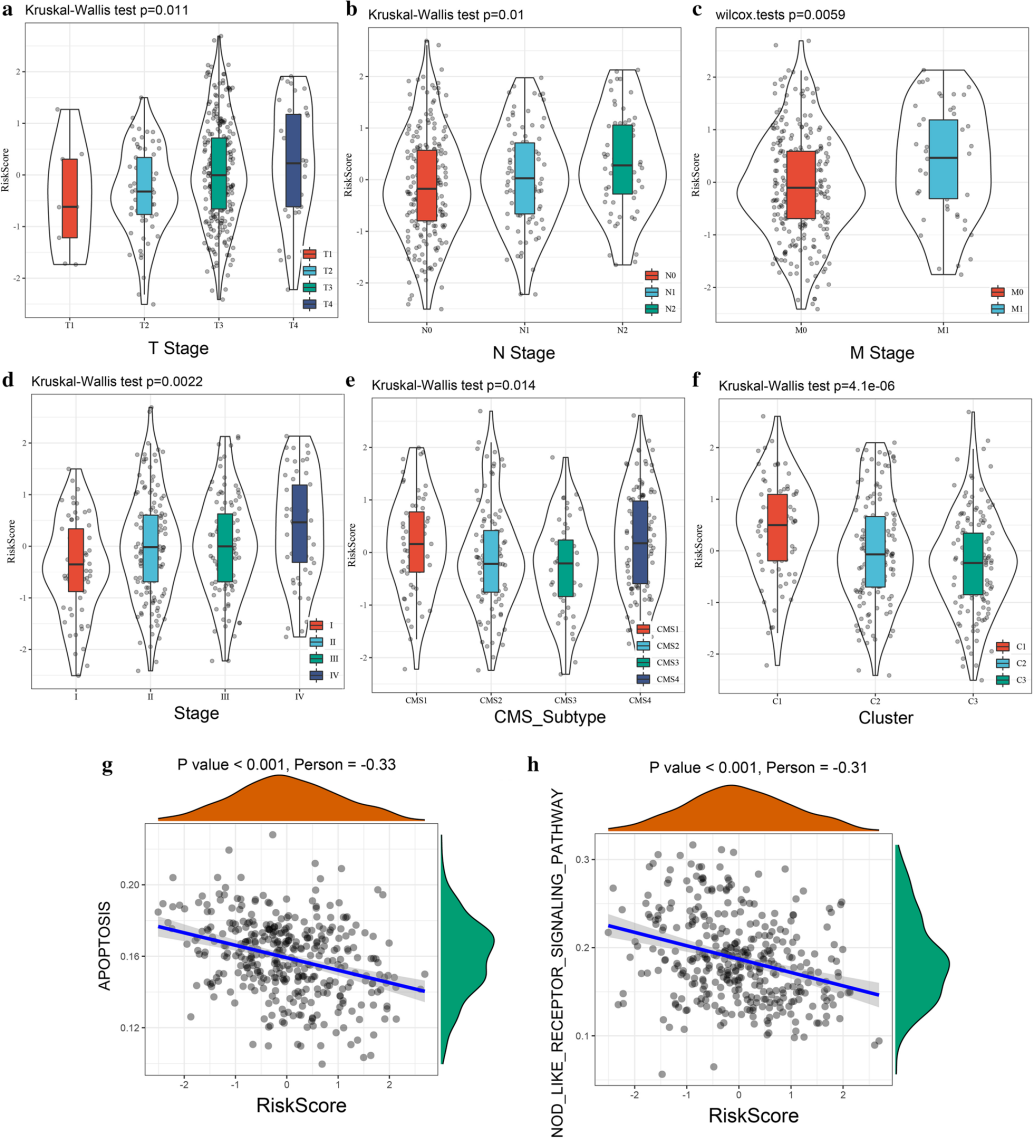
**a** comparison of the risk score in T Stage grouping samples; **b** comparison of the risk score in N Stage grouping samples; **c** comparison of the risk score in M Stage grouping samples; **d** comparison of the risk score in clinical stage grouping samples; **e** comparison of the risk score in CMS Subtypes; **f** comparison of the risk score in our molecular subtypes; **g** correlation between RiskScore with KKEGG_APOPTOSI; **h** correlation between RiskScore with KEGG_NOD_LIKE_ RECEPTOR_SIGNALING_PATHWAY

By comparing the risk score distribution with clinical features, we found that there were significant differences in T stage, N stage, M stage, clinical stage, and consensus molecular subtypes (P < 0.05; Fig. 9a–e). We also compared the risk score distribution with molecular subtypes. The results showed that the risk score of the C1 subtype with poor prognosis was significantly higher than the risk scores of the C2 and C3 subtypes (Fig. 9f).

We calculated the ssGSEA scores of each sample for different functions and calculated the correlations between these functions and risk score according to “Discussion” of the Methodology. Functions with correlation coefficients > 0.3 were selected, and there were significant negative correlations between KEGG_APOPTOSIS, KEGG_NOD_LIKE_RECEPTOR_SIGNALING_PATHWAY, and risk score (Fig. 9g–h).

### Univariate and multivariate analyses and nomogram construction

**Figure10:**
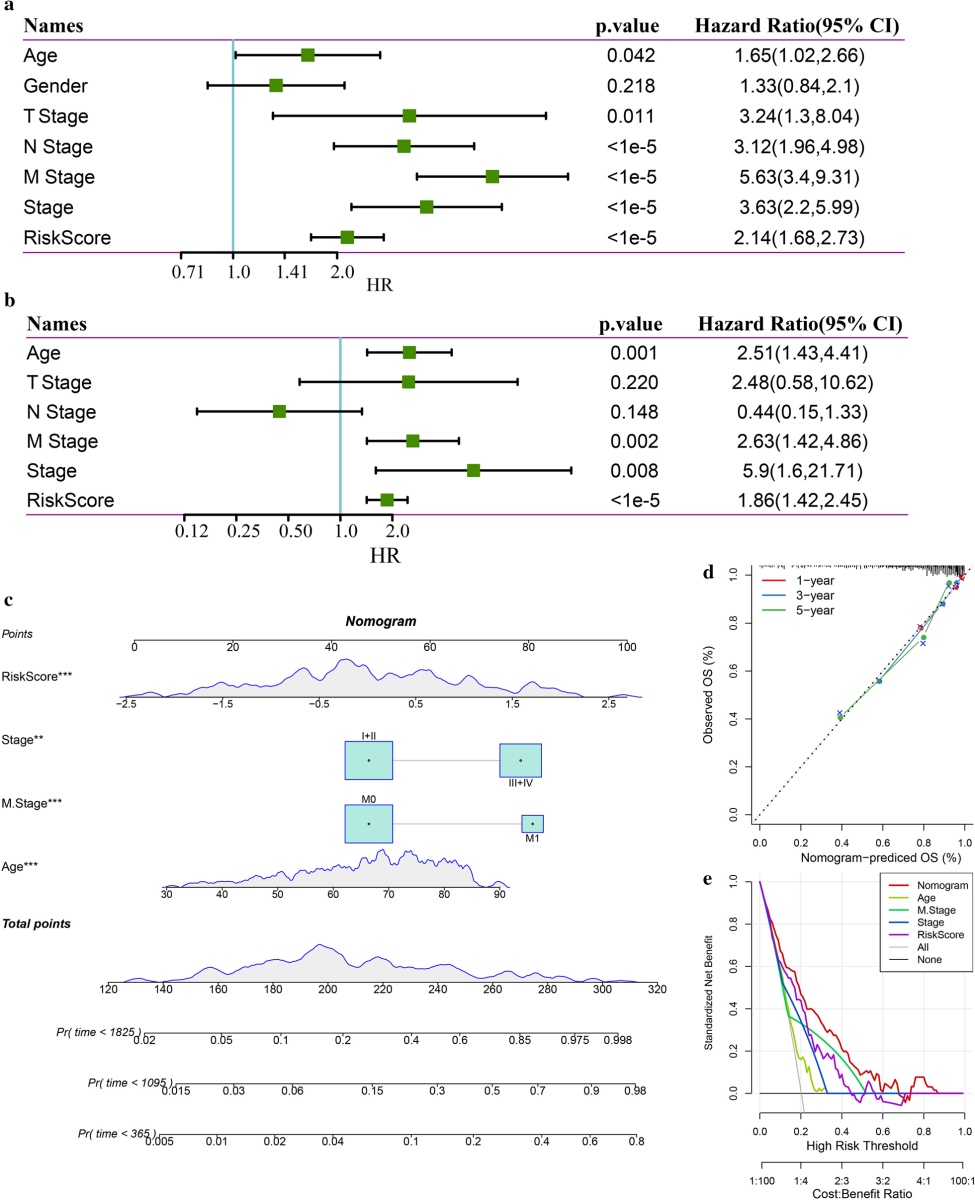
**a** Univariate analysis of clinical features and RiskScore; **b** multivariate analysis of clinical features and RiskScore; **c** construction of nomogram model; **d** the Calibration curves of 1-, 3-, 5- year in nomogram; **e** DCA analysis of Age, M Stage, clinical Stage, risk score and nomogram

Univariate Cox regression analysis results showed a significant correlation between risk score and prognosis. Multivariate Cox regression analysis results showed that risk score (HR = 1.86, 95% CI 1.42–2.45, P < 1e-5), clinical stage (HR = 5.9 s, 95% CI 1.60–21.71, P = 0.008), M stage (HR = 2.63, 95% CI 1.42–4.86, P = 0.002), and age (HR = 2.51, 95% CI 1.43–4.41, P = 0.001) were independent risk factors for prognosis in patients with colon cancer (Fig. 10a, b).

A nomogram was constructed with the variables that had the most significant values in the multivariate analysis (Fig. 10c). The results showed that risk score had the greatest influence on survival prediction, suggesting that the risk model based on 6 genes could predict prognosis well. The calibration curve analysis results showed that at 1, 3, and 5 years, the predicted calibration curve was close to the standard curve, which suggested that the risk model had good predictive performance (Fig. 10d).

The DCA curve showed that the risk score and nomogram benefits were higher than those of the extreme curve, while the nomogram’s benefit was higher than that of the risk score, and age, M stage, and clinical stage were close to the extreme curve, suggesting good reliability of the risk score and nomogram (Fig. 10e).

### Comparison of risk model with other models

**Fig. 11:**
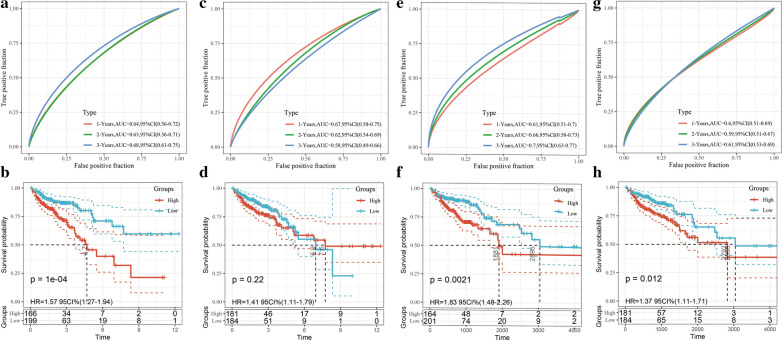
**a**, **b** The ROC of 15-gene signature (Xu) risk model and KM curves of the High/Low COAD samples; **c**, **d** the ROC of 15-gene signature (Dai) risk model and KM curves of the High/Low COAD samples; **e**, **f** the ROC of 12-gene signature (Sun) risk model and KM curves of the High/Low COAD samples; **g**, **h** the ROC of 9-gene signature (Mo) risk model and KM curves of the High/Low COAD samples

Four recurrence prognostic risk models were identified in the literature: a 15-gene signature by Xu et al., a 15-gene signature by Dai et al., a 12-gene signature by Sun et al., and a 9-gene signature by Mo et al. To make the models comparable, the same method was conducted to calculate the risk score of each COAD sample in the TCGA cohort based on the corresponding genes in these 4 models, and the Z-score method was used in the preprocessing of risk scores. Samples with risk scores > 0 were divided into the high-risk group, and samples with risk scores < 0 were divided into the low-risk group. The differences in COAD prognoses between the 2 groups were calculated. The results showed that the differences in COAD prognoses between the high- and low-risk groups in our model were significantly different from those in 3 of the other models (log-rank P < 0.05; Fig. 11b, f, h), excepting the 15-gene signature of Dai et al. (Fig. 11d). However, the AUCs of all 4 models were lower than the AUC of our 6-gene risk score model, whether at 1, 2, or 3 years (Fig. 11a, c, e, g). In summary, our 6-gene risk score model was found to be a more reasonable and efficient model with fewer genes.

### Correlations between gene expression and tumor immune cell infiltration

The results showed that the expression levels of the *GPRC5B*, *TIMP1*, and *TSPAN1* genes were significantly positively correlated with CD4+ T cells, macrophages, neutrophils, and dendritic cells. *GPRC5B* (R = 0.53, P = 1.9e−30) and *TSPAN1* (R = 0.581, P = 1.16e−37) had the strongest correlations with CD4+ T cells.

*CXCL13* had significantly positive correlations with 6 immune cells. Specifically, there were significant positive correlations with neutrophils (R = 0.61, P = 1.03e−42), dendritic cells (R = 0.59, P = 1.59e−39), and CD8+ T cells (R = 0.53, P = 3.39e−30).

**Fig. 12:**
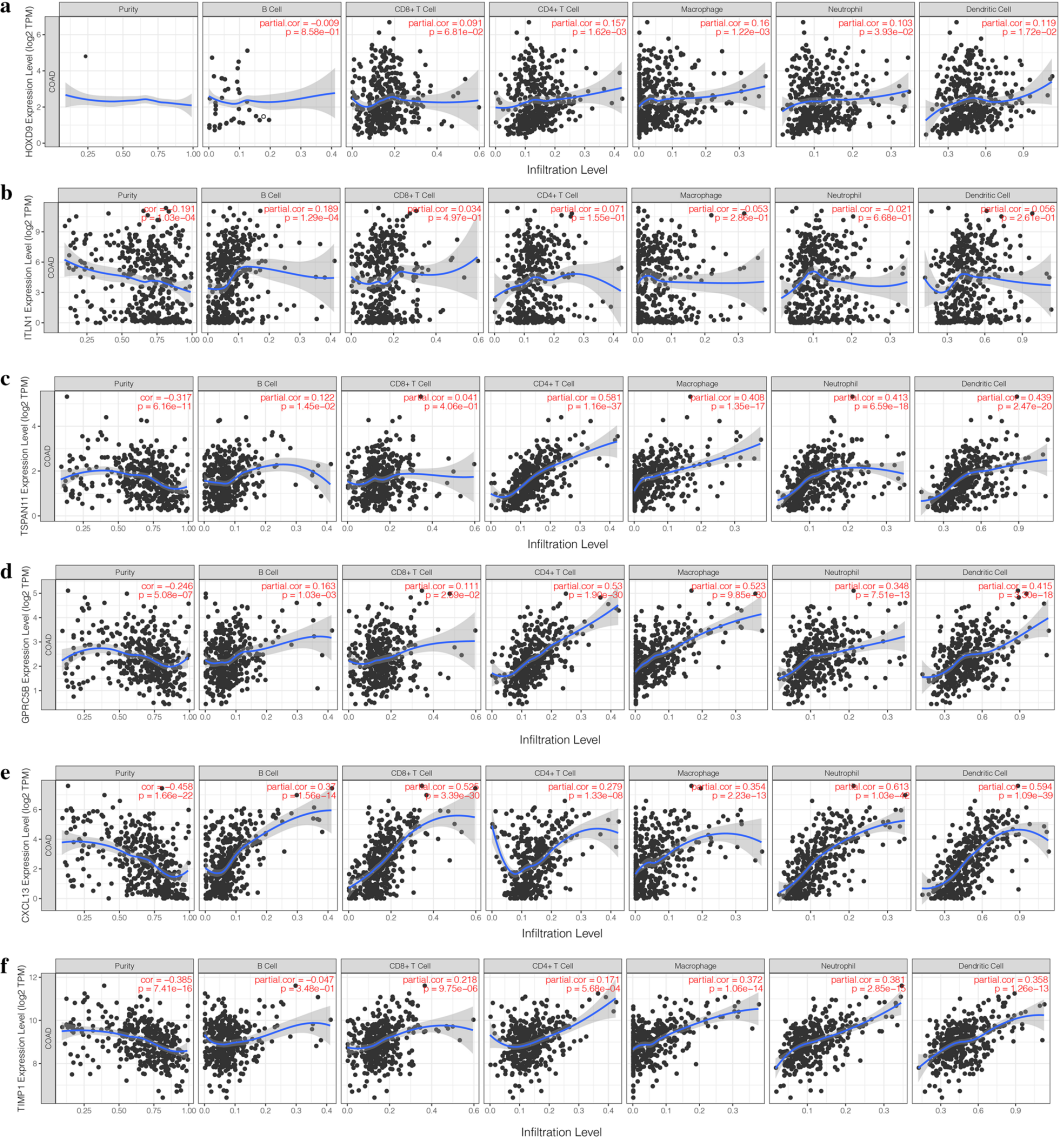
Correlation between the 8 genes’ expression and immune cell infiltration score; **a**–**g** correlation between the expression of HOXD9, ITLN1, TSPAN1, GPRC5B, CXCL13, TIMP1 with B cell, CD8+ T cell, CD4+ T cell, Macrophage, and Dendritic Cell, respectively. The horizontal axis refers to the infiltration level, and the vertical axis represents gene expression

*HOXD9* was positively correlated with CD4+ T cells, macrophages, neutrophils, and dendritic cells. *ITLN1* was only significantly correlated with B cells (R = 0.189, P = 1.29e−04). See Fig. 12a–f for related details.

### Gene expression in 33 pan-cancers

**Fig. 13:**
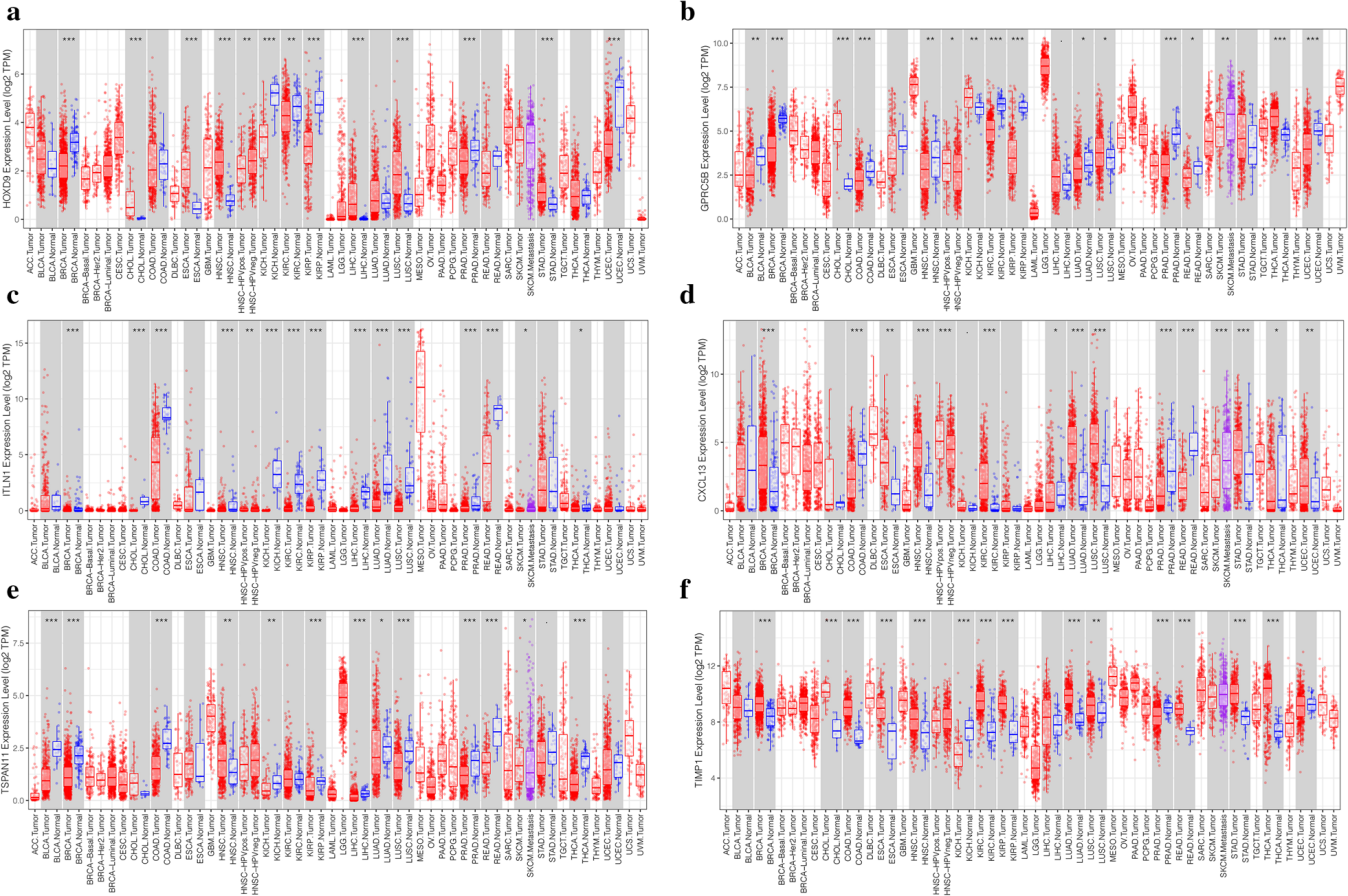
Expression box diagram of gene expression in pan-cancer. **a**–**f** The gene expression of HOXD9, CPRC5B, ITLN1, CXCL13, TSPAN11 and TIMP1 in different tumors, respectively

The box diagram showed that *HOXD9* expression was low in colon cancer with no significant difference; *HOXD9* was significantly lowly expressed in BRCA, UCEC, PRAD, KIRC, KIRP, and other tumors, while in CHOL, ESCA, HNSC, LUSC, and STAD tumors, *HOXD9* was significantly highly expressed and showed tissue specificity (Fig. 13a).

Compared with the expression levels in normal samples, *ITLN1*, *TSPAN11*, *CPRC5B*, and *CXCL13* showed significantly low expression levels in most cancer types, including COAD (Fig. 13b–e), while *TIMP1* was expressed highly in most cancer types (Fig. 13f).

### Clinical validation of genes via protein and mRNA expression levels

**Fig. 14:**
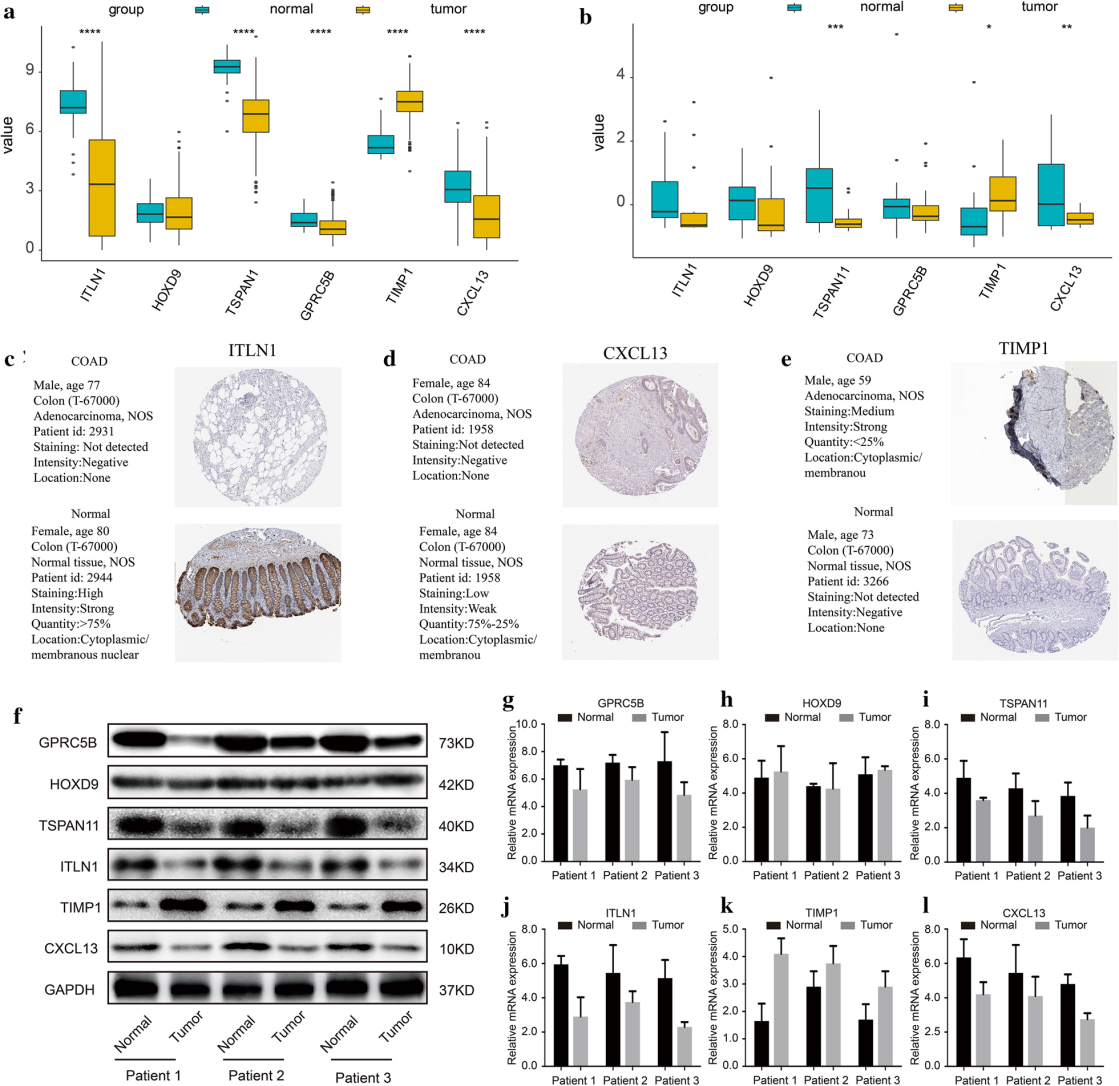
Clinical validation of 6 genes in protein and mRNA expression. **a** The expression box graph of 6 genes in TCGA-COAD; **b** the expression box graph of 6 genes in GSE10972; **c** ITLN1 protein expression in cancer and normal control; **d** CXCL13 protein expression in cancer and normal control; **e** TIMP1 protein expression in cancer and a normal control; **f** Westernblot expression of 6 genes in 3 pairs of cancer and adjacent normal tissues; **g**–**l** mRNA expression of GPRC5B, HOXD, TSPAN1, ITLN1, TIMP1 and CXCL13, respectively in 3 pairs of cancer and adjacent normal tissues

The TCGA-COAD and GSE10972 cohorts were used to verify the 6 genes’ expression levels in the cancer and normal samples with the ggplot2 R package. The box plots suggested that the *ITLN1*, *TSPAN11*, *CPRC5B*, and *CXCL13* genes were significantly lowly expressed in the colon cancer samples and the *TIMP1* gene was highly expressed in the colon cancer samples in the TCGA cohort (Fig. 14a).

In the GSE10972 cohort, *ITLN1* and *CPRC5B* did not show significant differences. They both showed a trend of low expression in the cancer samples (Fig. 14b). In general, the results from the GEO and TCGA databases were consistent.

Using the HPA database, the immunochemistry results of the 6 genes were analyzed. Only 3 genes (*ITLN1*, *TIMP1*, and *CXCL13*) had protein expression data. The results showed that the expression levels of the *ITLN1* and *CXCL13* genes in normal tissues were higher than those in colon cancer tissues, and the expression was located in the cytoplasm and cell membrane. *TIMP1* expression in colon cancer tissues was greater than that in normal tissues, and the expression was located in the cytoplasm and cell membrane (Fig. 14c–e), which was consistent with the expression trends of the GEO and TCGA cohorts.

Subsequently, we measured the protein expression levels of the 6 genes in 3 pairs of COAD tumor tissues and normal tissues. We found that, compared with normal tissues, the TIMP1 protein was expressed highly and significantly in cancer samples, while the ITLN1, TSPAN11, CPRC5B, and CXCL13 proteins were expressed poorly in cancer samples (Fig. 14f). Further, polymerase chain reaction analysis results showed that the mRNA expression trends of the 6 genes were consistent with protein expression levels (Fig. 14g–l).

## Discussion

The prognosis of CRC is poor. About half of patients with CRC die from recurrent, metastatic disease or complications. We conducted a comprehensive study to establish and validate a 6-gene signature, which was applied to explore the potential relationship between risk score and the survival rate of patients with colon cancer in order to provide a novel biomarker for predicting the prognosis of colon cancer.

A total of 365 COAD samples in the TCGA cohort were divided into 3 subtypes based on 97 invasion-related genes. The expression levels of genes in the C1, C2, and C3 subtypes were different, and most of the genes were overexpressed in the C1 subtype and under-expressed in the C3 subtype. There were significant differences in the prognoses of C1, C2, and C3. By using the limma package, 983 DEGs were found among the different subtypes. KEGG pathway analysis and GO functional enrichment analysis were performed to find DEGs that were significantly correlated with pathways of tumorigenesis and tumor development, including the intestinal immune network for IgA production, extracellular matrix-receptor interactions, the AGE-RAGE signaling pathway in diabetic complications, focal adhesions, and the PI3K-AKT signaling pathway. Subsequently, 17 of these genes were identified in the univariate Cox analysis, and 9 were selected as target genes by using Lasso regression. The stepwise regression algorithm was used to obtain 6 genes (*ITLN1*, *HOXD9*, *TSPAN11*, *GPRC5B*, *TIMP1*, and *CXCL13*) to construct a 6-gene signature as a prognostic risk model.

Omentin-1, also known as intelectin-1 (ITLN1), is a novel adipokine with 313 amino acids; it can be used as a marker of obesity and metabolic disorders, including insulin resistance, diabetes, and metabolic syndrome [18–20]. ITLN1 also acts as a tumor suppressor in various cancers, such as gastric cancer, ovarian cancer, neuroblastoma, and colon cancer [21–25]. Katsuya showed that the ITLN1 protein was lowly expressed in 87 of 148 CRC cases (59%) by immunohistochemistry; CRC cases with reduced ITLN1 expression had higher M grades than CRC cases in which ITLN1 was retained (P = 0.0017), and patients with retained ITLN1 expression tended to have more favorable prognoses than those with reduced ITLN1 expression [25]. The transcription factor HOXD9 is a member of the HOX family and plays an important role in tumorigenesis. By exploring the regulatory mechanism of HOXD9 on a molecular level, HOXD9 overexpression was found to significantly enhance the migration, invasion, and metastasis of hepatoma cells, gastric cancer cells, cervical cancer cells, and CRC cells [26–30]. The TSPAN11 protein has not been thoroughly studied in tumors. It has been reported that the direction of bone matrix organization is determined by fibrillar focal adhesion assembly mediated by TSPAN11. TSPAN11 silencing significantly destroys the arrangement of osteoblasts, and with further construction of bone matrix, the alignment is orthogonal [31]. G protein-coupled receptor class C group 5 member B (GPRC5B) controls the contractility and differentiation of smooth muscle [32]. There is no correlation between GPRC5B and tumors. However, molecular biological methods and knockout mouse studies have shown that proteins in the GPRC5 family play key roles in the control of tumor progression and metabolic homeostasis [33]. GPRC5A disorders have been associated with a variety of cancers, including non-small-cell lung carcinoma, breast cancer, CRC, liver cancer, and gastric cancer [34]. Tissue inhibitor of metalloproteinase 1 (TIMP1) is an intrinsic inhibitor of matrix metalloproteinases [35]. TIMP1 acts as an effective biomarker in patients with metastatic CRC with good sensitivity [36–38]. TIMP1 level increases in CRC have been associated with lymph node metastasis, distant metastasis, and vascular invasion, and TIMP1 mediation of the AK-PI3K/AKT and MAPK pathways may be involved in inhibiting proliferation, metastasis, and increased apoptosis [39]. Chemokine CXC ligand 13 (CXCL13) is an inflammatory factor in the microenvironment and plays a crucial role in the development of inflammatory diseases and tumors. The CXCL13 and chemokine receptor 5 (CXCR5) signaling axis has a key role in the occurrence and development of several human cancers [40, 41]. CXCL13 and CXCR5 are associated with poor prognosis in advanced colon cancer [42]. Further, the CXCL13-CXCR5 axis may promote the growth, migration, and invasion of colon cancer cells via the PI3K/AKT pathway [43].

The results of the prognostic analysis of risk models with clinical features suggested that our model had good predictive power in different clinical features, and the multivariate Cox analysis results showed that our risk model could be used as an independent prognostic risk factor. We also compared risk scores among molecular subtypes, and the results showed that the risk score of the C1 subtype with poor prognosis was significantly higher than the risk scores of the C2 and C3 subtypes. We further observed the relationships between risk score and biological functions in different samples and found that KEGG_APOPTOSIS and KEGG_NOD_LIKE_RECEPTOR_SIGNALING_PATHWAY were negatively correlated with risk score.

Dysregulation of the death mechanism of apoptotic cells is a mark of cancer. Apoptotic changes are not only responsible for tumorigenesis and cancer development, but also for the resistance of tumors to treatment [44–46]. In CRC development, the balancing between cell growth rate and apoptosis, which maintains the homeostasis of intestinal epithelial cells, is disturbed [47].

NOD-like receptors (NLRs) are cytosolic pattern-recognition receptors, and they are involved in mucosal immune defense. NLRs have been identified as key regulators of inflammation-related tumorigenesis, angiogenesis, cancer stemness, and chemical resistance, orchestrating the tumor microenvironment and enhancing the risk of tumors [48, 49]. The association between the NLR NOD2 and inflammatory bowel disease is indicative of the potential ability of NLRs to protect the gut barrier. NLRs work in maintaining intestinal homeostasis, protecting against intestinal infection, and shaping the gut microbiota [50]. Estrogen receptor alpha regulates the Wnt/β-catenin signaling pathway in colon cancer by targeting NLRs [51]. In short, NLRs are potential biomarkers for cancer.

In recent years, there have been more and more studies of colon cancer prognosis models. We selected 4 published gene signatures of colon cancer to demonstrate the superiority of our model. Xu et al. identified a 15-gene signature by using support vector machine analysis and 5 gene expression profile data confirmations, and it could distinguish high- and low-risk groups of colon cancer recurrence and predict prognosis [52]. Dai et al. developed an mRNA signature, and the 1-year AUC suggested that the predictive accuracy of the classifier was higher than that of the American Joint Commission on Cancer tumor-node-metastasis staging system. This mRNA signature may help detect early recurrence of stage I-III colon cancer to help patients with high-risk colon cancer receive more positive treatment interventions [53]. Sun et al. applied a 2-step supervised machine learning approach to establish a 12-gene signature. Seven of the 12 genes were involved in immune system function and regulation, so the signature may be used to guide decision-making in adjuvant therapy in patients with stage II-III and proficient DNA mismatch repair COAD [54]. Mo et al. established a 9-gene autophagy-related signature, which was found to be a reliable method for predicting the early relapse of stage I-III colon cancer [55]. The results of ROC analyses of these 4 models revealed that their 1-, 2-, and 3-year AUCs were all lower than those of our 6-gene signature, suggesting that our model had a reasonable number of genes and a significantly high discrimination ability to predict overall survival.

Finally, based on data from the GEO, TCGA, and HPA databases and our clinical samples, we found that compared with normal tissues, the TIMP1 protein was expressed highly and significantly in COAD samples, while the ITLN1, TSPAN11, CPRC5B, and CXCL13 proteins were poorly expressed in colon cancer samples.

There were some limitations in our study. The limited sample size may have led to selection bias. For better clinical application value, further studies with larger sample sizes are needed to support these findings, and more biological function studies should be conducted on the 6 genes in this research. Furthermore, animal studies and clinical practice should be conducted to test the predictive accuracy of our model and to identify potential invasion-related mechanisms.

## Conclusion

In this study, we constructed a 6-gene signature (ITLN1, HOXD9, TSPAN11, GPRC5B, TIMP1 and CXCL13) prognostic stratification system based on the colon cancer invasion-related genes, and evaluated the stability and accuracy of the model. The risk model had better AUC in both the training cohort and the independent validation cohort and was independent of clinical features. Therefore, we recommend this classifier as a molecular diagnostic test to assess the prognostic risk in patients with colon cancer.

## Supplementary Information

**Additional file 1: Table S1.** 97 invasion-related genes obtained from the CancerSEA.

**Additional file 2: Table S2.** Eight genes associated with the prognosis of colon carcinoma

**Additional file 3: Table S3.** Differentially expressed genes between C1 ~ C3 cluster.

**Additional file 4: Table S4.** Differentially expressed genes between C1 ~ C2 cluster.

**Additional file 5: Table S5.** Differentially expressed genes between C2 ~ C3 cluster.

**Additional file 6: Table S6.** 17 genes related to prognosis based on 983 DEGs

## Data Availability

The datasets used during the current study are available from the corresponding author on reasonable request.
