# Supplementary material for: Identification and development of a novel invasion-related gene signature for prognosis prediction in colon adenocarcinoma
Source: Cancer Cell Int. 2021 Feb 12;21:101. doi: 10.1186/s12935-021-01795-1 (PMC7881672; doi:10.1186/s12935-021-01795-1)
Supplement: Supplementary file 1 — Additional file 1: Table S1. 97 invasion-related genes obtained from the CancerSEA. [file 12935_2021_1795_MOESM1_ESM.docx]

p.value HR Low 95%CI High 95%CI

AEBP1 0.09284682 1.135349002 0.979117237 1.316509718

AKR1B1 0.852579646 1.018896643 0.836334588 1.241309858

AMD1 0.250655792 0.829496356 0.602974665 1.141116275

SLC25A5 0.153597417 0.796377061 0.582506761 1.088770923

ATP5PB 0.042275346 0.673195675 0.459491161 0.986291913

BAG1 0.304833458 1.25640722 0.812427665 1.943014954

BGN 0.042958212 1.17754673 1.00519327 1.379452432

C1QB 0.203837731 1.107138647 0.946287742 1.295331143

CALD1 0.221008106 1.113350459 0.937458914 1.322243809

CAPG 0.85742815 1.02374543 0.792497461 1.322470742

CCNE1 0.530334345 0.895931713 0.635633862 1.262823904

CDH11 0.307927388 1.106667626 0.910755684 1.344722033

CKS1B 0.201973896 1.272734311 0.878722432 1.843417861

CKS2 0.597525171 0.918699855 0.670598595 1.258591101

COL1A1 0.224255172 1.091826957 0.947591458 1.258016938

COL1A2 0.297132303 1.08402331 0.931473184 1.261557024

COL3A1 0.347720389 1.075878651 0.92356095 1.253317251

COL5A1 0.210696396 1.119428646 0.938151324 1.335733864

COL5A2 0.355410585 1.08412274 0.913445607 1.286690861

COL6A2 0.172179498 1.127631511 0.949018477 1.339860978

COL6A3 0.61053025 1.042352897 0.888566437 1.222755572

COL10A1 0.154828858 1.082194529 0.970609161 1.206608227

COL11A1 0.226112996 1.089100299 0.94852023 1.250515723

COMP 0.119045348 1.087041028 0.978749943 1.207313682

CSE1L 0.282745002 0.834436574 0.599743576 1.160970162

VCAN 0.305625437 1.094650821 0.920732831 1.301420325

CTSK 0.739663246 0.970317201 0.812322435 1.15904157

DAB2 0.593372962 0.936222271 0.73505694 1.192441146

DDX5 0.435636908 0.823036079 0.504396024 1.342969325

EDNRA 0.484281539 1.072762459 0.881101415 1.306114453

FAP 0.072296598 1.177789916 0.985293679 1.407894027

FBN1 0.384896116 1.080467028 0.907393073 1.28655269

FN1 0.035795521 1.145458652 1.009050988 1.300306465

GNAS 0.546469788 1.108162954 0.793670189 1.547273856

H2AFZ 0.189804008 0.778064113 0.534689408 1.132215741

HMGB2 0.954234403 1.008665722 0.751229054 1.35432267

HNRNPU 0.416719846 1.213016555 0.761140276 1.933164241

HSD17B4 0.023199629 0.66534347 0.46802491 0.945851223

CCN1 0.066229883 1.179192922 0.989014519 1.405940885

INHBA 0.105590967 1.154162841 0.970186794 1.373026175

LAMB1 0.179327652 1.216042058 0.914027765 1.617848325

LAMC1 0.21324348 1.223743645 0.890447775 1.681792633

LGALS1 0.557325849 1.060857355 0.870889423 1.292263172

LOX 0.236790332 1.124063937 0.926072834 1.364384841

LOXL2 0.350869365 1.113924162 0.888004924 1.39732

LUM 0.650870437 1.033918672 0.894843922 1.194608125

MMP2 0.514207896 1.052898541 0.901823941 1.229281334

MMP11 0.507611714 1.048563745 0.911322179 1.206473356

HNRNPM 0.949729626 1.015385096 0.631676956 1.6321743

NDUFB7 0.620622326 0.932591776 0.707419565 1.229436481

YBX1 0.185968859 0.752512003 0.493762638 1.146855332

PDGFRB 0.115434622 1.178592739 0.960535979 1.446151808

PLAU 0.695497603 0.955995004 0.763109888 1.197634132

PRRX1 0.122338658 1.150541896 0.963039465 1.374550786

PNN 0.07542048 1.383628441 0.967294997 1.979155964

PPIC 0.359991167 1.173550857 0.833083141 1.653162268

PROS1 0.872451054 0.983928762 0.807352357 1.199124274

PSMA2 0.868094001 0.975638205 0.729260051 1.305254422

PSMB4 0.97489695 1.007883164 0.618021072 1.643679345

RGS4 0.169752173 1.175315258 0.933273027 1.48013059

SNAI2 0.378335223 1.116590353 0.873655687 1.427077092

SPOCK1 0.090849657 1.139165243 0.979483043 1.324879957

TGFBI 0.55119549 0.947375012 0.793066565 1.131707544

THBS2 0.131602861 1.102321788 0.971209292 1.251134369

THY1 0.194053335 1.150631431 0.931046864 1.422004348

TNFAIP6 0.147145851 1.1426568 0.954144481 1.368413891

UBE2V2 0.667928334 0.941777038 0.71601699 1.238719196

ADAM12 0.062331435 1.169067794 0.991984232 1.377763337

MFAP5 0.223041134 1.104318763 0.941403315 1.295427699

ITGBL1 0.038704303 1.219371955 1.010352532 1.471632839

TP53I3 0.119734575 0.752556232 0.526034469 1.076623142

NUAK1 0.01645473 1.37287558 1.059673648 1.778648892

HNRNPDL 0.480085171 1.181403271 0.743801254 1.876460523

TXNDC9 0.58588366 0.926356702 0.703497496 1.219814916

LRRC17 0.277676029 1.186469386 0.87133811 1.615572172

IFI30 0.014730206 1.587050014 1.094948563 2.300316043

POSTN 0.156065718 1.099473748 0.964442215 1.253411043

CBX1 0.584698202 1.107483281 0.767956839 1.597119987

NID2 0.665167231 1.053154295 0.832977233 1.331529752

RRAS2 0.172189983 1.202755249 0.92272009 1.567777928

RALY 0.79435812 0.952056329 0.657990041 1.37754555

SEPHS2 0.879236136 1.030049225 0.703060488 1.50911824

HEY1 0.058855583 1.334195757 0.989269202 1.799387179

MXRA5 0.938011112 1.006358815 0.857778458 1.180675562

OLFML2B 0.074219043 1.187720397 0.983305716 1.43462986

TMEM158 0.202498609 0.8715675 0.705491302 1.07673887

WWTR1 0.075311321 1.199529956 0.981615607 1.465820333

GREM1 0.40280614 1.061533837 0.922950043 1.220926414

NOX4 0.02415325 1.371990949 1.042206411 1.806128945

CLEC4A 0.424446953 0.90123135 0.698250586 1.163218423

COPZ2 0.196024578 1.161824588 0.92554123 1.458429217

ASPN 0.150151502 1.102507272 0.965286911 1.259234194

CEMIP 0.936204322 0.992682937 0.829298841 1.188256107

CRISPLD2 0.07300828 1.201582213 0.983020585 1.468738128

TUBB6 0.231930898 1.140952392 0.919120248 1.416324321

LRRC15 0.918311208 1.00915665 0.847830964 1.201179467

TUBB 0.803376235 0.942991057 0.594062155 1.496867163
