# Supplementary material for: Identification and development of a novel invasion-related gene signature for prognosis prediction in colon adenocarcinoma
Source: Cancer Cell Int. 2021 Feb 12;21:101. doi: 10.1186/s12935-021-01795-1 (PMC7881672; doi:10.1186/s12935-021-01795-1)
Supplement: Supplementary file 2 — Additional file 2: Table S2. Eight genes associated with the prognosis of colon carcinoma [file 12935_2021_1795_MOESM2_ESM.docx]

p.value HR Low 95%CI High 95%CI

ATP5PB 0.042275346 0.673195675 0.459491161 0.986291913

BGN 0.042958212 1.17754673 1.00519327 1.379452432

FN1 0.035795521 1.145458652 1.009050988 1.300306465

HSD17B4 0.023199629 0.66534347 0.46802491 0.945851223

ITGBL1 0.038704303 1.219371955 1.010352532 1.471632839

NUAK1 0.01645473 1.37287558 1.059673648 1.778648892

IFI30 0.014730206 1.587050014 1.094948563 2.300316043

NOX4 0.02415325 1.371990949 1.042206411 1.806128945
