# Supplementary material for: Identification and development of a novel invasion-related gene signature for prognosis prediction in colon adenocarcinoma
Source: Cancer Cell Int. 2021 Feb 12;21:101. doi: 10.1186/s12935-021-01795-1 (PMC7881672; doi:10.1186/s12935-021-01795-1)
Supplement: Supplementary file 3 — Additional file 3: Table S3. Differentially expressed genes between C1 ~ C3 cluster. [file 12935_2021_1795_MOESM3_ESM.docx]

logFC AveExpr t P.Value adj.P.Val B

AEBP1 -3.091794141 6.174865279 -23.2842848 4.06E-62 6.30E-58 130.8728466

SPOCK1 -2.794935332 2.170827921 -22.5716085 5.69E-60 4.41E-56 125.9695764

FN1 -3.747978513 6.243188093 -22.44335908 1.39E-59 7.20E-56 125.0806011

GFPT2 -2.233105619 1.786006386 -22.13699308 1.19E-58 4.63E-55 122.9489424

SSC5D -2.376302088 1.95940561 -21.90514051 6.11E-58 1.89E-54 121.3282663

GAS1 -2.797070281 1.984495303 -21.85885777 8.47E-58 2.19E-54 121.0039805

GGT5 -2.069167787 3.062700498 -21.83530297 1.00E-57 2.22E-54 120.8388436

ZNF469 -1.659016932 1.36413223 -21.64955677 3.72E-57 7.22E-54 119.5343358

NTM -2.098265075 1.805114118 -21.52068661 9.28E-57 1.60E-53 118.6269008

BGN -2.919934028 7.524318902 -21.41960271 1.90E-56 2.95E-53 117.9137726

CCDC8 -1.833813673 1.645717108 -21.31518075 4.00E-56 5.65E-53 117.1758573

FNDC1 -3.05982406 3.016785709 -20.97802979 4.45E-55 5.75E-52 114.784831

COL6A2 -2.495258626 7.145956938 -20.83772059 1.22E-54 1.43E-51 113.7860049

MRC2 -2.301615491 4.133556822 -20.82966932 1.29E-54 1.43E-51 113.7286235

COL1A1 -3.194469787 8.670477901 -20.81624656 1.42E-54 1.47E-51 113.6329433

ARSI -1.690709335 1.321084512 -20.74118716 2.43E-54 2.36E-51 113.0975369

COL10A1 -3.753143145 2.912749072 -20.48119439 1.58E-53 1.44E-50 111.2382034

MXRA8 -2.167707392 4.836547731 -20.45714648 1.88E-53 1.62E-50 111.065854

SCARF2 -2.066888856 2.687470732 -20.31123726 5.40E-53 4.25E-50 110.0188035

MEIS3 -1.520974923 1.866676718 -20.30873537 5.50E-53 4.25E-50 110.0008301

GLI3 -1.461143331 1.006911139 -20.30253908 5.75E-53 4.25E-50 109.9563133

CAVIN1 -2.018977304 6.080390518 -20.20109539 1.20E-52 8.45E-50 109.2269192

CSDC2 -1.449240122 0.984054743 -19.99816197 5.23E-52 3.53E-49 107.7645577

ADAMTS10 -1.280403036 1.279490546 -19.9837265 5.81E-52 3.60E-49 107.660371

EVC -1.734792988 1.554834496 -19.96754085 6.53E-52 3.90E-49 107.543527

SFRP2 -4.803633643 4.319281542 -19.96033001 6.88E-52 3.95E-49 107.4914633

EFEMP2 -1.880732182 3.692400519 -19.94946281 7.45E-52 4.13E-49 107.4129899

ITGA11 -2.297974388 2.613126292 -19.88287184 1.21E-51 6.46E-49 106.931864

PLPP4 -2.313955893 1.74128017 -19.82600135 1.83E-51 9.45E-49 106.5206109

MRAS -1.639927791 1.965017249 -19.77273501 2.70E-51 1.35E-48 106.1351233

OLFML2B -2.305002988 4.043118469 -19.73615583 3.52E-51 1.71E-48 105.8702347

ADAM12 -2.466252263 2.157832834 -19.71596368 4.08E-51 1.92E-48 105.7239554

THBS2 -3.431249858 4.815009534 -19.49738923 2.01E-50 9.18E-48 104.1379267

P4HA3 -1.587465734 1.29324028 -19.44133385 3.03E-50 1.34E-47 103.7304187

MAP1A -1.669041738 1.840194186 -19.41287169 3.74E-50 1.61E-47 103.5233895

GLI2 -1.358685522 1.21873898 -19.39032738 4.41E-50 1.85E-47 103.3593501

HHIPL1 -1.141624034 0.97143144 -19.36246839 5.41E-50 2.21E-47 103.156572

ITGA5 -2.092183518 4.362057706 -19.3021046 8.41E-50 3.35E-47 102.7169446

COL1A2 -2.87295077 7.943774064 -19.25508779 1.19E-49 4.61E-47 102.3742822

MMP14 -1.876522346 7.24836394 -19.22952331 1.43E-49 5.42E-47 102.1878783

TNS1 -2.368327631 3.334118875 -19.19370462 1.86E-49 6.88E-47 101.9266019

FBLN2 -2.949132239 3.803549949 -19.15111281 2.55E-49 9.19E-47 101.6157628

ISM1 -2.035731515 1.412561496 -19.10222994 3.65E-49 1.29E-46 101.2588025

SYDE1 -1.587922735 2.984885229 -19.08810562 4.05E-49 1.40E-46 101.1556203

KCNE4 -1.688887307 1.892960235 -19.08362692 4.19E-49 1.41E-46 101.1228983

ADAMTS2 -2.473109792 3.578844582 -18.95414679 1.09E-48 3.53E-46 100.1761009

ATP10A -1.351692163 1.380632703 -18.95050169 1.12E-48 3.53E-46 100.1494248

CDH2 -1.42735447 0.973352198 -18.95042963 1.12E-48 3.53E-46 100.1488974

CERCAM -2.155596968 3.664846798 -18.89958033 1.62E-48 5.03E-46 99.77663772

PDGFRB -2.092134616 4.867599205 -18.86512036 2.09E-48 6.36E-46 99.52422951

EFS -1.434192741 1.724508609 -18.83341144 2.64E-48 7.88E-46 99.29187804

MFGE8 -1.829920778 4.82094092 -18.82792208 2.75E-48 8.06E-46 99.25164493

HLX -1.246402768 2.014349893 -18.77595845 4.04E-48 1.16E-45 98.87065587

COL8A2 -2.097385254 2.269112923 -18.77027374 4.21E-48 1.19E-45 98.82896199

COL11A1 -2.969756508 2.88186939 -18.76683174 4.32E-48 1.20E-45 98.80371563

TGFB3 -2.281656546 2.300489427 -18.74468466 5.09E-48 1.38E-45 98.64124657

CMTM3 -1.734232524 3.962118983 -18.70320826 6.91E-48 1.85E-45 98.33686361

EHD2 -2.084411377 4.691600746 -18.65471232 9.89E-48 2.60E-45 97.98077654

ARHGEF25 -1.707099996 2.248356518 -18.56523719 1.92E-47 4.96E-45 97.32326319

CLIP3 -1.647980684 2.765304881 -18.5407986 2.30E-47 5.84E-45 97.14355597

ST6GALNAC5 -1.53355081 1.239153558 -18.52710227 2.54E-47 6.36E-45 97.04281898

HTRA3 -2.484880019 4.913205121 -18.51681322 2.74E-47 6.75E-45 96.96713223

FBXL7 -1.424151441 1.752772554 -18.51321545 2.82E-47 6.83E-45 96.94066479

SERPING1 -2.37155795 5.709497514 -18.45268552 4.41E-47 1.05E-44 96.49520546

C3orf80 -1.512147094 1.432781708 -18.39819109 6.61E-47 1.55E-44 96.09390231

COL6A1 -2.280708888 6.779348238 -18.27521262 1.64E-46 3.81E-44 95.18737977

GPR68 -1.775488768 2.533477694 -18.27284573 1.67E-46 3.82E-44 95.1699205

CCDC80 -2.482509943 2.619960418 -18.25375765 1.93E-46 4.34E-44 95.02910131

THY1 -1.97394002 5.245667669 -18.18824587 3.14E-46 6.95E-44 94.54557794

COL5A1 -2.404444328 5.523572357 -18.18134476 3.30E-46 7.21E-44 94.49462301

TAGLN -2.557692379 6.464186019 -18.1693588 3.61E-46 7.78E-44 94.40611483

MYL9 -2.310221026 6.802045495 -18.12324214 5.09E-46 1.08E-43 94.0654693

EMILIN1 -2.246293894 5.487853035 -18.03350923 9.92E-46 2.08E-43 93.40217474

TMEM200B -1.485677639 1.55339349 -18.01793391 1.11E-45 2.30E-43 93.28698097

CCDC184 -1.032750482 0.948074871 -18.01186162 1.16E-45 2.38E-43 93.24206583

SLC22A17 -1.451595788 1.838793995 -18.00292458 1.25E-45 2.51E-43 93.17595576

ANGPTL2 -2.250357833 4.543540759 -17.99923225 1.28E-45 2.54E-43 93.14864067

NOTCH3 -1.92411606 4.361503417 -17.99398798 1.33E-45 2.61E-43 93.10984289

TUBB6 -1.810621806 4.020543732 -17.98470987 1.43E-45 2.76E-43 93.04119716

FLNA -2.262863093 7.012203044 -17.97921122 1.49E-45 2.84E-43 93.00051132

DNAJB5 -1.304322003 1.52531618 -17.9623377 1.68E-45 3.19E-43 92.875646

PRELP -2.639645604 2.166694847 -17.91655664 2.37E-45 4.43E-43 92.53675517

BOC -1.657736357 1.366174448 -17.90308226 2.62E-45 4.84E-43 92.4369824

ACTA2 -2.201880573 6.873762864 -17.87749798 3.17E-45 5.78E-43 92.24750333

VIM -1.847931208 6.937177585 -17.86752223 3.41E-45 6.16E-43 92.17360916

PRRX1 -2.155526896 2.343489177 -17.84629416 4.00E-45 7.13E-43 92.01634048

ISLR -2.70802255 5.351071716 -17.79986178 5.65E-45 9.96E-43 91.67223052

GLIS2 -1.853974292 2.861599778 -17.78620098 6.26E-45 1.09E-42 91.5709608

SYNDIG1 -1.699179268 1.581294864 -17.73424239 9.23E-45 1.59E-42 91.18566109

PCOLCE -2.022363631 4.728995121 -17.6870473 1.31E-44 2.24E-42 90.83551922

TWIST2 -1.424793855 1.232580165 -17.5924609 2.66E-44 4.48E-42 90.13331211

HS3ST3A1 -1.112365592 0.923236059 -17.55960062 3.40E-44 5.67E-42 89.88921454

ADGRA2 -1.785061636 3.370378518 -17.52155413 4.52E-44 7.46E-42 89.60650085

TIMP2 -2.160149214 6.065943584 -17.44604242 7.95E-44 1.30E-41 89.04510725

AOC3 -2.307755568 3.214409932 -17.43851353 8.41E-44 1.36E-41 88.98911299

SPHK1 -1.99123469 2.766565326 -17.42154074 9.55E-44 1.53E-41 88.86286848

FAP -2.132083025 2.07511365 -17.41249984 1.02E-43 1.62E-41 88.79561418

TENM4 -1.101722092 0.768947198 -17.38127526 1.29E-43 2.02E-41 88.56329711

MRGPRF -2.048877026 2.762215865 -17.33942181 1.77E-43 2.74E-41 88.25180142

IFFO1 -1.204910568 2.060397833 -17.33706482 1.80E-43 2.76E-41 88.23425616

COL8A1 -2.463174489 2.948047488 -17.32387874 1.98E-43 3.02E-41 88.13609331

PTGIR -1.075944431 1.248021892 -17.29469639 2.47E-43 3.70E-41 87.91880872

TIE1 -1.543983454 2.551218509 -17.28766319 2.60E-43 3.84E-41 87.86643323

C1R -2.052103583 5.695919032 -17.23194828 3.95E-43 5.78E-41 87.45142265

COL6A3 -2.504733705 5.43139137 -17.22884068 4.05E-43 5.86E-41 87.42826913

GSDME -1.136906093 0.990794397 -17.22325214 4.22E-43 6.06E-41 87.3866295

MSC -1.604572279 2.973474819 -17.21697091 4.42E-43 6.29E-41 87.33982649

FBN1 -2.28314687 3.504658876 -17.20151144 4.97E-43 6.99E-41 87.22462386

FAM180A -1.141509653 0.725083367 -17.20044346 5.01E-43 6.99E-41 87.21666486

VEGFC -1.487806844 2.038205415 -17.1967487 5.15E-43 7.13E-41 87.18912946

SULF1 -2.774955745 5.024060164 -17.17033147 6.27E-43 8.53E-41 86.99222972

COMP -3.527527922 2.808851275 -17.14975959 7.32E-43 9.87E-41 86.83886926

HTRA1 -1.924613028 5.93001296 -17.14759667 7.44E-43 9.95E-41 86.82274349

HAND2 -1.672888991 0.92602403 -17.14530771 7.57E-43 1.00E-40 86.80567781

TGFB1I1 -1.45785583 3.765496779 -17.12079463 9.10E-43 1.20E-40 86.62289708

GPR176 -1.42387918 1.991772569 -17.11144092 9.76E-43 1.27E-40 86.5531423

COPZ2 -1.670673184 2.860423713 -17.06647996 1.37E-42 1.77E-40 86.21777718

MEIS1 -1.231192972 1.299156683 -17.05239949 1.52E-42 1.95E-40 86.11272657

SFRP4 -3.478019961 3.626702848 -16.99974687 2.26E-42 2.87E-40 85.71979979

COL3A1 -2.657355283 8.739201089 -16.97960825 2.63E-42 3.31E-40 85.5694716

PDLIM7 -1.484988692 4.938503156 -16.97439382 2.73E-42 3.41E-40 85.53054393

NOX4 -1.235713774 0.947998264 -16.95811028 3.09E-42 3.83E-40 85.40897152

SLC11A1 -1.5847497 1.663926254 -16.94932274 3.30E-42 4.06E-40 85.34335795

FNDC4 -1.322477113 2.139143962 -16.93147468 3.77E-42 4.60E-40 85.21007955

ITGAM -1.915294277 1.995917207 -16.90943147 4.45E-42 5.39E-40 85.04545037

TRO -1.00090482 1.027741025 -16.89738641 4.87E-42 5.85E-40 84.9554811

MMP2 -2.470115041 6.507232416 -16.88478555 5.35E-42 6.39E-40 84.86135199

PLEKHO1 -1.439089968 3.238904167 -16.86312242 6.30E-42 7.44E-40 84.69950739

CYS1 -1.461761765 1.410820836 -16.86238871 6.33E-42 7.44E-40 84.69402549

COL5A2 -2.348614005 5.57751941 -16.85526536 6.68E-42 7.79E-40 84.64080141

CD248 -2.074996075 5.055047821 -16.85322457 6.79E-42 7.85E-40 84.62555263

LZTS1 -1.398946147 1.80551962 -16.79289297 1.07E-41 1.22E-39 84.17465607

NKX3-2 -1.209395282 1.013624892 -16.78129331 1.17E-41 1.32E-39 84.08794284

RCN3 -1.761269193 5.057695287 -16.71220433 1.96E-41 2.20E-39 83.57132769

LAYN -1.475335153 2.150261687 -16.69280803 2.27E-41 2.53E-39 83.42624853

DACT3 -1.401139863 1.370187908 -16.67602869 2.57E-41 2.85E-39 83.30072867

CTHRC1 -2.576157661 4.921508544 -16.64553523 3.24E-41 3.56E-39 83.07258385

ADAMTS12 -1.90246151 2.489651734 -16.59924754 4.59E-41 4.98E-39 82.72618566

NLGN2 -1.597726918 2.637956932 -16.58805919 4.99E-41 5.38E-39 82.6424415

SYNC -1.294162772 1.061154882 -16.58161248 5.24E-41 5.60E-39 82.59418564

TNC -2.624707027 4.220870996 -16.5751073 5.50E-41 5.85E-39 82.54549017

NNMT -1.96755086 4.613589858 -16.54875886 6.71E-41 7.05E-39 82.34823511

NXN -1.879924488 2.70525824 -16.54843966 6.73E-41 7.05E-39 82.34584525

MSRB3 -1.988878097 2.71680679 -16.52653107 7.93E-41 8.26E-39 82.18180437

LTBP2 -2.01491499 3.539944059 -16.50798528 9.12E-41 9.43E-39 82.04292563

ARHGEF17 -1.505362948 2.820731915 -16.48349737 1.10E-40 1.12E-38 81.85952641

CHST1 -1.23188927 1.516016169 -16.45256653 1.39E-40 1.40E-38 81.62783611

TAFA5 -1.56425511 2.076120712 -16.44495329 1.47E-40 1.48E-38 81.5708021

VSTM4 -1.370617367 1.677362454 -16.4207903 1.76E-40 1.76E-38 81.38977052

VASN -1.557593084 3.413066821 -16.37804195 2.43E-40 2.40E-38 81.06943529

CCN4 -1.80474282 2.423434435 -16.32519597 3.62E-40 3.55E-38 80.67332965

ATP8B2 -1.565579863 2.386963671 -16.25561498 6.12E-40 5.97E-38 80.15161817

SPARC -2.125942621 8.594395465 -16.24374639 6.69E-40 6.49E-38 80.06261007

P3H1 -1.009980107 3.635589707 -16.23088038 7.37E-40 7.10E-38 79.96611578

JAM3 -1.285474494 2.770315131 -16.22667913 7.61E-40 7.29E-38 79.93460535

LMOD1 -2.436852127 3.18413001 -16.22398263 7.77E-40 7.39E-38 79.91438051

CNN1 -2.890486627 4.152478485 -16.22089597 7.95E-40 7.52E-38 79.89122905

FXYD6 -1.770924419 2.166281572 -16.21420148 8.36E-40 7.86E-38 79.84101587

RAB31 -2.128534646 4.191993978 -16.21268548 8.46E-40 7.90E-38 79.82964464

CDH11 -1.926067685 3.130908386 -16.1183963 1.72E-39 1.59E-37 79.12223508

LDOC1 -1.654226805 2.483019019 -16.10857411 1.86E-39 1.70E-37 79.0485256

LRRC32 -1.586308625 4.432037296 -16.10384481 1.92E-39 1.75E-37 79.01303391

DDR2 -1.883275074 2.217807575 -16.08064936 2.29E-39 2.08E-37 78.8389497

PLPP7 -1.076351 1.227251839 -16.07487254 2.39E-39 2.16E-37 78.79559129

GYPC -1.533636485 2.752672711 -16.0574392 2.73E-39 2.45E-37 78.66473717

NRP2 -1.713856939 2.581551282 -16.05088268 2.87E-39 2.56E-37 78.6155215

LOX -1.910531587 3.05312407 -16.03977309 3.12E-39 2.77E-37 78.53212565

HSPB8 -2.007765239 2.488968466 -16.03470262 3.24E-39 2.86E-37 78.49406211

MMP19 -1.526585118 2.255179865 -16.01326218 3.81E-39 3.34E-37 78.33310145

ADAMTS4 -1.661632773 2.48451451 -15.9652554 5.48E-39 4.77E-37 77.97264621

INHBA -2.152005202 3.366977292 -15.90763897 8.47E-39 7.30E-37 77.53994603

HSPB7 -2.010908826 1.567085589 -15.90214414 8.83E-39 7.56E-37 77.49867479

CHRD -1.100191617 1.247396718 -15.87977314 1.05E-38 8.91E-37 77.33063896

ANTXR1 -2.407544571 4.478323862 -15.87693838 1.07E-38 9.03E-37 77.30934515

FBLN5 -1.657167642 2.831671789 -15.87646349 1.07E-38 9.03E-37 77.30577787

MEDAG -1.901602274 2.051283517 -15.84596715 1.35E-38 1.13E-36 77.07668488

SLIT3 -1.6116729 1.555654693 -15.84371355 1.37E-38 1.14E-36 77.05975446

SERPINF1 -1.942518423 5.338105707 -15.73548833 3.11E-38 2.58E-36 76.24654863

PLEKHO2 -1.391236178 4.123657756 -15.71544655 3.62E-38 2.99E-36 76.0959229

REM1 -1.028497051 1.142716847 -15.70691555 3.86E-38 3.17E-36 76.03180458

CALHM5 -1.132151382 1.125933344 -15.68411759 4.59E-38 3.75E-36 75.86044887

ADAMTS7 -1.247393228 1.899954331 -15.66192935 5.43E-38 4.41E-36 75.69366493

PDLIM3 -1.795134306 2.475667432 -15.65604068 5.67E-38 4.58E-36 75.64939941

AMOTL1 -1.616763133 2.015333959 -15.64112848 6.35E-38 5.10E-36 75.53729997

MFAP5 -2.222760304 1.884305166 -15.63717234 6.54E-38 5.21E-36 75.50755971

RFTN1 -1.474581985 3.706599991 -15.63715198 6.55E-38 5.21E-36 75.50740665

PXDN -1.833815949 3.752573638 -15.62429785 7.21E-38 5.71E-36 75.41077358

CRYAB -1.815588397 2.961035334 -15.58567821 9.66E-38 7.60E-36 75.1204239

JPH2 -1.543995045 1.285007006 -15.58504409 9.71E-38 7.60E-36 75.11565625

TSHZ3 -1.323047294 1.686337324 -15.56021392 1.17E-37 9.13E-36 74.92896285

C20orf194 -1.144832944 1.23025537 -15.51571939 1.64E-37 1.27E-35 74.59438864

VCAN -2.228785647 3.824525311 -15.507809 1.74E-37 1.34E-35 74.53490325

PPP1R18 -1.439817082 4.644820286 -15.49759434 1.88E-37 1.44E-35 74.45808844

PDLIM4 -1.751762567 2.753775407 -15.48576154 2.06E-37 1.57E-35 74.36910297

BNC2 -1.042211085 0.779699526 -15.48350203 2.09E-37 1.59E-35 74.35211068

TIMP3 -1.799349752 2.016894886 -15.48193337 2.12E-37 1.60E-35 74.34031376

DPYSL3 -2.234342285 4.409285175 -15.47794668 2.18E-37 1.64E-35 74.3103321

GAS7 -1.542869071 2.243869507 -15.44300844 2.84E-37 2.12E-35 74.04757095

OSCAR -1.553152062 2.234833742 -15.41560736 3.50E-37 2.60E-35 73.84148199

LHFPL6 -1.61802623 3.95070349 -15.41440127 3.53E-37 2.61E-35 73.83241051

FAM20C -1.761623456 3.725750694 -15.4060788 3.76E-37 2.76E-35 73.76981331

FAM20A -1.259693291 1.264964217 -15.3497155 5.76E-37 4.21E-35 73.3458543

TENM3 -1.227595376 0.744610003 -15.34502576 5.97E-37 4.35E-35 73.31057679

AKT3 -1.398006383 1.867128225 -15.33831366 6.28E-37 4.53E-35 73.26008607

FGFR1 -1.612234888 2.637372619 -15.33363667 6.51E-37 4.67E-35 73.22490378

FRMD6 -1.560460224 1.926045806 -15.32744333 6.82E-37 4.87E-35 73.17831456

ITGBL1 -1.783744254 1.253404063 -15.31119285 7.71E-37 5.49E-35 73.0560687

SPON2 -1.630561989 4.934994101 -15.28729013 9.24E-37 6.54E-35 72.876253

LOXL2 -1.684630773 4.701896565 -15.27871596 9.86E-37 6.95E-35 72.81174968

CHSY3 -1.011127577 1.137212236 -15.2710042 1.05E-36 7.34E-35 72.75373369

KCNMB1 -1.577659367 1.380937108 -15.26486192 1.10E-36 7.65E-35 72.70752453

MARCO -2.674428803 1.759656542 -15.26179435 1.12E-36 7.79E-35 72.68444674

JCAD -1.622513715 2.290825324 -15.25601443 1.17E-36 8.11E-35 72.6409632

COL18A1 -1.795641478 5.071343239 -15.25322514 1.20E-36 8.24E-35 72.61997871

LOXL1 -1.65295809 3.405758856 -15.25074984 1.22E-36 8.36E-35 72.60135631

FLT4 -1.143162216 1.614629987 -15.24946094 1.23E-36 8.41E-35 72.59165956

PTGIS -2.086180643 1.821474769 -15.24594501 1.26E-36 8.60E-35 72.56520815

POPDC2 -1.357798525 1.549637354 -15.22112796 1.52E-36 1.03E-34 72.37849893

FIBIN -2.004680831 3.157960883 -15.1820203 2.05E-36 1.37E-34 72.08426577

ZNF521 -1.238264033 1.435701494 -15.13838762 2.85E-36 1.90E-34 71.755976

MXRA5 -2.310168512 4.124676962 -15.12746964 3.10E-36 2.05E-34 71.67382811

HSPA12B -1.299385216 2.194012554 -15.09301112 4.02E-36 2.65E-34 71.41455555

RTL8B -1.49794987 2.506959811 -15.08924464 4.14E-36 2.72E-34 71.38621553

BMP1 -1.103252196 4.024144365 -15.08175707 4.38E-36 2.87E-34 71.32987685

TMEM204 -1.407150759 4.04274916 -15.0604187 5.15E-36 3.35E-34 71.16931982

SLIT2 -1.456515301 1.0589442 -15.05283571 5.45E-36 3.54E-34 71.11226256

COL16A1 -1.664885164 3.590229362 -15.02425549 6.77E-36 4.37E-34 70.89721317

NUAK1 -1.408726479 2.34480525 -14.99623035 8.37E-36 5.36E-34 70.68633934

CILP -2.077009617 1.172162205 -14.98264674 9.28E-36 5.92E-34 70.58413

FSTL3 -1.802845229 4.177333148 -14.96008551 1.10E-35 6.99E-34 70.41436892

PODNL1 -1.390456964 2.27980966 -14.94145021 1.27E-35 8.02E-34 70.27414864

CHST3 -1.498364439 2.19194642 -14.93539665 1.33E-35 8.36E-34 70.22859903

ADAMTS16 -1.233794227 0.713305468 -14.93400229 1.34E-35 8.42E-34 70.2181073

MITF -1.301944602 1.486245934 -14.92710069 1.41E-35 8.83E-34 70.16617679

PDGFB -1.331812068 3.592268433 -14.92639556 1.42E-35 8.85E-34 70.16087111

EMP3 -1.601657747 4.72753283 -14.92509855 1.43E-35 8.90E-34 70.1511119

NAP1L3 -1.110298096 0.911340173 -14.91558491 1.54E-35 9.52E-34 70.07952752

PLA2G5 -1.029583695 0.783219765 -14.91416669 1.56E-35 9.59E-34 70.06885629

MN1 -1.375334436 1.376986687 -14.90393076 1.68E-35 1.03E-33 69.99183739

A4GALT -1.727588435 2.987764284 -14.89332359 1.82E-35 1.11E-33 69.91202542

PCDH7 -1.351712557 1.416111121 -14.89175223 1.85E-35 1.12E-33 69.90020206

MSN -1.539079345 5.91847528 -14.87548906 2.09E-35 1.26E-33 69.7778332

CNTNAP1 -1.47653829 1.999768616 -14.85164428 2.50E-35 1.50E-33 69.59841996

DOK5 -1.095687876 1.012195226 -14.84773529 2.58E-35 1.54E-33 69.56900812

SH2D3C -1.236474983 2.468367028 -14.83801423 2.77E-35 1.65E-33 69.49586574

BCAT1 -1.421987059 1.544180533 -14.81760102 3.24E-35 1.92E-33 69.34227582

LBH -1.523294604 4.85006789 -14.80813663 3.48E-35 2.05E-33 69.27106613

CLMP -1.692412177 2.339792388 -14.80244656 3.63E-35 2.13E-33 69.22825452

VGLL3 -1.244051256 0.919973406 -14.77687471 4.41E-35 2.58E-33 69.03585667

C14orf132 -1.259343309 1.250052583 -14.77070619 4.62E-35 2.69E-33 68.98944661

POSTN -2.820444651 5.449966732 -14.75103973 5.36E-35 3.11E-33 68.84148391

SORCS2 -1.276943068 1.307348117 -14.74601566 5.56E-35 3.22E-33 68.80368527

NID2 -1.548840312 2.964563713 -14.73532396 6.03E-35 3.48E-33 68.7232469

FOXS1 -1.305246433 2.314984378 -14.73285613 6.15E-35 3.53E-33 68.7046805

PLXDC1 -1.073098623 1.978186062 -14.73054322 6.26E-35 3.58E-33 68.68727963

COL15A1 -1.951901052 4.598613465 -14.70757137 7.44E-35 4.23E-33 68.51445665

HSD17B14 -1.451900796 2.516716594 -14.69573229 8.14E-35 4.61E-33 68.42539023

SERPINH1 -1.07512724 6.936616997 -14.67119252 9.80E-35 5.53E-33 68.24078005

KANK2 -1.234272808 4.229013804 -14.66153517 1.05E-34 5.93E-33 68.16813059

RAB34 -1.598449356 3.366233403 -14.63185546 1.32E-34 7.39E-33 67.94486536

GPNMB -2.480416112 4.39996469 -14.61907288 1.45E-34 8.12E-33 67.84871186

PODN -1.929905376 3.004020375 -14.60597162 1.61E-34 8.93E-33 67.75016336

MEOX2 -1.4636888 0.85331893 -14.59674273 1.72E-34 9.54E-33 67.68074445

GLI1 -1.259157679 1.69857502 -14.5840136 1.90E-34 1.05E-32 67.58499897

HIC1 -1.198816896 1.929723593 -14.57691883 2.00E-34 1.10E-32 67.5316347

COL12A1 -2.121725858 5.110051608 -14.55334646 2.39E-34 1.31E-32 67.35433751

AXL -1.651156345 3.619749211 -14.54729257 2.50E-34 1.37E-32 67.30880512

CRISPLD2 -1.708279557 3.501913354 -14.54291509 2.59E-34 1.41E-32 67.27588164

COLEC12 -1.544053813 1.410842959 -14.54253011 2.60E-34 1.41E-32 67.27298618

PCDH12 -1.065923858 2.135501937 -14.51898921 3.10E-34 1.67E-32 67.09593815

TSPYL5 -1.21455118 1.479573641 -14.50015684 3.58E-34 1.91E-32 66.95430889

CALHM2 -1.365186404 3.087089053 -14.49501754 3.72E-34 1.98E-32 66.91565977

ZNF532 -1.364028654 2.348749043 -14.49326137 3.77E-34 2.00E-32 66.90245292

ARL4C -1.798788903 4.330069528 -14.48171637 4.11E-34 2.17E-32 66.8156329

TSPAN4 -1.394606763 3.052809012 -14.47898659 4.20E-34 2.21E-32 66.79510494

TWIST1 -1.728552359 1.98433795 -14.47195483 4.43E-34 2.32E-32 66.74222663

FBLN1 -2.315415143 4.531818328 -14.46903177 4.53E-34 2.36E-32 66.72024571

GREM1 -2.525662722 3.49859274 -14.44335805 5.50E-34 2.85E-32 66.52719041

OLR1 -2.018662099 1.927645158 -14.44046956 5.62E-34 2.91E-32 66.50547101

MAB21L2 -2.069141016 2.569977976 -14.42185335 6.47E-34 3.32E-32 66.36549418

DLG4 -1.086269371 1.973243827 -14.42082729 6.52E-34 3.34E-32 66.35777938

GUCY1A1 -1.767526683 2.308942773 -14.41556564 6.79E-34 3.45E-32 66.31821793

MGP -2.406313997 5.01768079 -14.40539469 7.33E-34 3.72E-32 66.24174596

THBS4 -2.430079112 1.604153879 -14.3913589 8.15E-34 4.12E-32 66.1362191

IGFBP5 -2.363270648 6.233147591 -14.38668898 8.44E-34 4.25E-32 66.10110962

SGCD -1.284990957 1.268888509 -14.33516613 1.25E-33 6.24E-32 65.71378177

PHYHIP -1.037217328 0.829515829 -14.33309739 1.27E-33 6.32E-32 65.69823109

RGMA -1.304701003 1.070008617 -14.31071925 1.50E-33 7.46E-32 65.53002151

CCN2 -1.757642413 6.883172811 -14.30092208 1.62E-33 7.99E-32 65.45638299

RAB3IL1 -1.468853628 2.804601023 -14.29921458 1.64E-33 8.06E-32 65.44354919

ZBTB47 -1.235692143 3.123770308 -14.28647429 1.80E-33 8.85E-32 65.34779337

ARHGEF15 -1.165201172 1.96584571 -14.25619284 2.27E-33 1.11E-31 65.12021532

MPDZ -1.134211337 1.163515376 -14.23505861 2.66E-33 1.30E-31 64.9613969

CLEC11A -1.520012976 4.258861886 -14.22581013 2.85E-33 1.39E-31 64.8919007

ASPN -2.60366774 3.395265806 -14.22501077 2.87E-33 1.39E-31 64.88589414

VASH1 -1.283695053 2.660577615 -14.22420757 2.89E-33 1.39E-31 64.8798588

PTPRM -1.190010564 2.365941472 -14.21865917 3.01E-33 1.45E-31 64.83816763

SEMA6B -1.353241023 2.608337261 -14.21591828 3.07E-33 1.48E-31 64.81757261

HMCN1 -1.129460769 0.97139216 -14.20268794 3.40E-33 1.62E-31 64.71816323

ITGAX -1.620494493 2.497736719 -14.19703181 3.54E-33 1.69E-31 64.67566607

HK3 -1.72193914 1.991724965 -14.18451526 3.90E-33 1.85E-31 64.58162652

NEXN -1.742970343 2.208292041 -14.18119046 4.00E-33 1.89E-31 64.55664735

HEYL -1.35886997 3.210140089 -14.18031338 4.02E-33 1.90E-31 64.55005794

COL4A2 -1.594472516 6.765929463 -14.1715203 4.30E-33 2.02E-31 64.48399748

HAPLN3 -1.684131363 3.395699554 -14.16937833 4.37E-33 2.05E-31 64.46790571

HEG1 -1.612091689 3.097416619 -14.16868491 4.39E-33 2.05E-31 64.46269637

PALM -1.55750806 1.99430868 -14.16215246 4.61E-33 2.14E-31 64.41362153

SLC1A3 -1.340165723 1.223454214 -14.15122792 5.01E-33 2.32E-31 64.33155426

TMEM119 -1.752116873 3.672118535 -14.14071626 5.43E-33 2.50E-31 64.252592

HOPX -1.682325239 1.966809569 -14.13558205 5.64E-33 2.60E-31 64.21402575

CPXM1 -1.810288186 3.912662982 -14.10196833 7.27E-33 3.34E-31 63.96155265

EGR2 -1.714147582 2.168045625 -14.0972706 7.54E-33 3.44E-31 63.92627085

LAMB2 -1.417843725 5.108739006 -14.09029081 7.94E-33 3.61E-31 63.87385121

APOE -2.740012743 6.212881975 -14.07395159 8.99E-33 4.08E-31 63.75114667

CPXM2 -1.880252809 2.083909656 -14.06851567 9.37E-33 4.23E-31 63.71032592

PRKD1 -1.29449258 1.439674361 -14.06622951 9.53E-33 4.30E-31 63.69315839

CTIF -1.044700416 3.053186833 -14.05923215 1.00E-32 4.52E-31 63.64061407

LILRB4 -1.705109242 2.124372728 -14.03801333 1.18E-32 5.29E-31 63.4812889

COX7A1 -1.360061142 3.3085816 -14.01380921 1.42E-32 6.31E-31 63.29956751

DKK3 -1.549793818 4.695027225 -14.01038245 1.45E-32 6.46E-31 63.27384149

LGALS1 -1.721594685 8.255104542 -14.00978115 1.46E-32 6.47E-31 63.26932738

RBPMS2 -1.499475129 1.58428818 -14.0048308 1.52E-32 6.70E-31 63.23216409

C1S -2.010822778 5.746648692 -13.99502212 1.63E-32 7.15E-31 63.15853102

LRRC15 -2.036425725 2.406797098 -13.97021578 1.97E-32 8.58E-31 62.97232749

GALNT15 -1.183501161 0.946701169 -13.96856254 1.99E-32 8.66E-31 62.95991862

CALD1 -1.986602447 5.159920568 -13.96398911 2.06E-32 8.94E-31 62.92559199

RHOJ -1.2891064 2.299134658 -13.92815029 2.70E-32 1.17E-30 62.65662563

SLC12A4 -1.127979572 3.339422281 -13.92717377 2.72E-32 1.17E-30 62.64929766

BCL6B -1.251249751 2.266434328 -13.90259943 3.28E-32 1.41E-30 62.4649003

TNFSF4 -1.366935162 1.785954113 -13.89010888 3.60E-32 1.54E-30 62.37118496

PRRX2 -1.698995953 2.035249158 -13.8879523 3.66E-32 1.57E-30 62.35500502

STON1 -1.180434671 1.267160637 -13.86313309 4.42E-32 1.88E-30 62.16881038

SLC15A3 -1.438351101 3.198640882 -13.86055018 4.51E-32 1.91E-30 62.14943483

BASP1 -1.846456137 3.798852979 -13.84019188 5.26E-32 2.23E-30 61.99672786

KIAA1755 -1.120281393 1.62554416 -13.82601988 5.85E-32 2.47E-30 61.89043474

CYP1B1 -1.890273507 1.340410976 -13.82479046 5.90E-32 2.49E-30 61.88121421

MMRN2 -1.305135032 2.834175676 -13.79418332 7.44E-32 3.13E-30 61.65168597

SLC24A3 -1.683580331 2.611908843 -13.77301399 8.73E-32 3.64E-30 61.49295832

TNFAIP6 -1.924312816 2.481105029 -13.77296227 8.73E-32 3.64E-30 61.49257052

PRR16 -1.21832602 1.782684508 -13.76461725 9.30E-32 3.86E-30 61.43000519

ZEB2 -1.202361583 1.534805402 -13.75007243 1.04E-31 4.29E-30 61.32096581

FERMT2 -1.531083334 2.595389285 -13.7419273 1.10E-31 4.55E-30 61.2599079

TPM2 -1.750704256 6.104807184 -13.73590713 1.15E-31 4.75E-30 61.21478122

MAFB -1.794205762 3.306364594 -13.72517808 1.25E-31 5.14E-30 61.13436149

PTGER3 -1.039682169 0.871110653 -13.71219413 1.38E-31 5.64E-30 61.03704759

C3 -2.661351397 5.10450617 -13.71211697 1.38E-31 5.64E-30 61.03646932

SYT11 -1.352862283 2.486483551 -13.70640556 1.44E-31 5.87E-30 60.99366524

OSMR -1.505080766 2.649903124 -13.69870834 1.53E-31 6.21E-30 60.93598109

RFLNB -1.218119436 3.321102749 -13.69489421 1.57E-31 6.37E-30 60.90739855

GLT8D2 -1.518380798 2.559228781 -13.67909858 1.77E-31 7.16E-30 60.78903592

GPC6 -1.519749973 1.963032183 -13.65965088 2.05E-31 8.25E-30 60.64332421

FEZ1 -1.014453131 1.516429363 -13.6405668 2.37E-31 9.50E-30 60.50035575

APBB1 -1.287813988 2.173968131 -13.60276907 3.15E-31 1.26E-29 60.21725009

RASL12 -1.252441229 2.662389658 -13.58739742 3.54E-31 1.41E-29 60.10213796

SHISAL1 -1.020447472 0.774604966 -13.58649536 3.56E-31 1.41E-29 60.09538322

SNAI2 -1.451097635 2.891354839 -13.57988688 3.74E-31 1.48E-29 60.04589921

PLXDC2 -1.754924651 2.725249344 -13.57311394 3.94E-31 1.56E-29 59.99518619

MMP11 -2.352114728 5.387822407 -13.54151958 5.00E-31 1.96E-29 59.75865413

DEPP1 -1.690683924 4.439284039 -13.53023711 5.44E-31 2.13E-29 59.67420116

ECM2 -1.367660978 1.446190298 -13.52708542 5.57E-31 2.18E-29 59.65061101

CHST11 -1.487711064 2.505595731 -13.52425649 5.69E-31 2.22E-29 59.62943718

SLC2A3 -1.6330665 3.356732898 -13.51575304 6.07E-31 2.36E-29 59.56579373

NUMBL -1.024977584 2.809414849 -13.50431083 6.62E-31 2.55E-29 59.48016201

EBF4 -1.256780208 1.9687381 -13.49878148 6.90E-31 2.65E-29 59.43878388

MFAP2 -1.879594475 3.951561436 -13.49605904 7.04E-31 2.70E-29 59.41841154

MAP1B -1.277049957 1.71593071 -13.49119646 7.31E-31 2.78E-29 59.38202534

FSTL1 -1.644354093 5.468095493 -13.4881324 7.48E-31 2.84E-29 59.35909806

DPT -2.290223737 2.676112985 -13.48747573 7.51E-31 2.85E-29 59.35418445

GRASP -1.038043604 2.364900133 -13.47853948 8.04E-31 3.04E-29 59.28732102

GNAI2 -1.031154313 6.073343428 -13.45668592 9.47E-31 3.57E-29 59.12382671

LYNX1 -1.061748509 0.949482224 -13.45395039 9.67E-31 3.64E-29 59.10336329

NPR1 -1.137309365 1.585575573 -13.4497774 9.98E-31 3.74E-29 59.07214759

CHRNA3 -1.256467349 0.869932776 -13.44218506 1.06E-30 3.95E-29 59.01535648

MRVI1 -1.485086525 2.783820619 -13.4361098 1.11E-30 4.12E-29 58.96991569

C1QTNF6 -1.161284193 2.998555892 -13.41033538 1.34E-30 4.99E-29 58.77715751

COL5A3 -1.555315118 3.031292863 -13.39291579 1.53E-30 5.67E-29 58.6469056

OLFML1 -1.34110453 2.139152404 -13.38106428 1.67E-30 6.18E-29 58.55829898

ROR2 -1.638989045 2.02750251 -13.37936803 1.70E-30 6.25E-29 58.54561785

AKAP12 -2.113288649 2.642397502 -13.35751352 2.00E-30 7.31E-29 58.38225062

TNS2 -1.122811629 3.516711861 -13.32528888 2.55E-30 9.27E-29 58.14142097

PSD -1.264288596 1.473027983 -13.31991969 2.65E-30 9.63E-29 58.10130116

NPR3 -1.090255455 0.622458819 -13.29701927 3.15E-30 1.14E-28 57.93020561

HSD17B6 -1.154038142 1.798339447 -13.28907886 3.34E-30 1.20E-28 57.87088878

FMNL1 -1.243566371 2.6309568 -13.2838422 3.48E-30 1.25E-28 57.83177201

FGR -1.54430833 2.399539856 -13.28205788 3.53E-30 1.26E-28 57.81844392

C5orf46 -1.271884854 0.783757904 -13.26092481 4.13E-30 1.48E-28 57.6606057

SHISA4 -1.448926383 2.813080798 -13.25418978 4.35E-30 1.55E-28 57.61030975

SLAMF8 -1.706350575 2.955254442 -13.24551648 4.64E-30 1.65E-28 57.54554375

NDN -1.624105547 3.449050899 -13.23955585 4.85E-30 1.72E-28 57.50103713

EFEMP1 -2.115991041 3.779671297 -13.2370475 4.95E-30 1.75E-28 57.48230858

SRPX -1.550605962 2.55553515 -13.23428224 5.05E-30 1.78E-28 57.46166232

SEMA7A -1.54816905 2.854039626 -13.23309332 5.09E-30 1.79E-28 57.45278569

C5AR1 -1.564429994 3.154270738 -13.21323413 5.91E-30 2.08E-28 57.30452884

CLEC5A -1.250436667 1.178267624 -13.20553551 6.27E-30 2.19E-28 57.24706309

GPR4 -1.062639852 2.509516618 -13.19483662 6.79E-30 2.37E-28 57.16720908

LOXL4 -1.024913228 1.106553364 -13.16982534 8.20E-30 2.84E-28 56.98056319

RTL5 -1.000535399 1.068210178 -13.16600464 8.43E-30 2.92E-28 56.95205537

TSHZ2 -1.050452827 0.960729955 -13.14021152 1.02E-29 3.54E-28 56.75963052

TRPV2 -1.348523138 3.21027972 -13.13698249 1.05E-29 3.61E-28 56.7355444

SPARCL1 -1.908002246 5.231805538 -13.12134951 1.18E-29 4.06E-28 56.61894541

CFH -1.625925259 2.744889683 -13.11232116 1.26E-29 4.32E-28 56.55161559

S1PR3 -1.233306519 2.056851737 -13.10679763 1.32E-29 4.49E-28 56.51042642

LAMP5 -1.487782666 1.307819635 -13.09341309 1.45E-29 4.96E-28 56.41062707

MYO5A -1.071168847 1.554001805 -13.09054232 1.49E-29 5.06E-28 56.38922347

LAMA4 -1.350960585 3.802336657 -13.06987349 1.74E-29 5.88E-28 56.23514189

CACNA2D1 -1.044883208 1.00260305 -13.05446013 1.95E-29 6.57E-28 56.12026049

PMP22 -1.47582724 5.135674257 -13.04315257 2.12E-29 7.12E-28 56.03599299

ANGPTL1 -1.327561236 0.780126383 -13.03517273 2.25E-29 7.53E-28 55.97653078

SIGLEC9 -1.148069689 1.380629635 -13.03212347 2.30E-29 7.69E-28 55.95381045

CLEC14A -1.227144799 3.416315605 -13.01742256 2.57E-29 8.55E-28 55.84428289

ITGB2 -1.941291253 4.079860385 -13.0150089 2.62E-29 8.68E-28 55.8263018

FCGR1A -1.155607999 1.200201994 -13.0102784 2.71E-29 8.96E-28 55.79106235

EHD3 -1.18073854 2.242315534 -12.99703992 3.00E-29 9.85E-28 55.6924531

CDK14 -1.333114847 1.665246192 -12.97916736 3.43E-29 1.12E-27 55.55934872

SERPINE1 -1.944250145 4.657473562 -12.95526456 4.10E-29 1.34E-27 55.38137603

BTBD19 -1.050150978 1.702166712 -12.95102068 4.23E-29 1.38E-27 55.34978247

MYH11 -2.695418665 3.71946163 -12.94471388 4.44E-29 1.44E-27 55.30283419

UNC5B -1.428470069 3.860748024 -12.93955086 4.61E-29 1.49E-27 55.26440285

SDK1 -1.051792808 1.040750904 -12.9301408 4.95E-29 1.60E-27 55.19436413

SPI1 -1.654636811 4.06417023 -12.92869237 5.00E-29 1.61E-27 55.18358415

CNRIP1 -1.057713207 1.961811243 -12.92793469 5.03E-29 1.62E-27 55.17794518

CD34 -1.164959467 3.124533455 -12.89342185 6.52E-29 2.08E-27 54.9211387

MAF -1.343839498 2.794705955 -12.88416782 6.99E-29 2.23E-27 54.85229796

MYLK -1.703888813 3.119505361 -12.88096325 7.16E-29 2.28E-27 54.82846089

DAAM2 -1.125385884 1.82332007 -12.87929647 7.25E-29 2.30E-27 54.81606294

ENG -1.22381652 5.633501079 -12.85086979 8.96E-29 2.83E-27 54.604656

SV2A -1.041480656 1.294558042 -12.83909032 9.79E-29 3.09E-27 54.51707399

PKD2 -1.191860377 3.039301424 -12.83818935 9.86E-29 3.10E-27 54.51037567

PLN -2.071623449 2.399954848 -12.8381417 9.86E-29 3.10E-27 54.51002137

TUBA1A -1.566115332 5.742171933 -12.81313056 1.19E-28 3.73E-27 54.3241036

LATS2 -1.080732926 3.23626316 -12.80798103 1.24E-28 3.86E-27 54.28583208

KIFC3 -1.267509278 2.590153467 -12.80654328 1.25E-28 3.90E-27 54.2751471

CYGB -1.247028314 3.924093735 -12.80503343 1.26E-28 3.93E-27 54.26392656

DIPK2B -1.057998739 1.916397354 -12.79618879 1.35E-28 4.19E-27 54.19820089

GJA5 -1.342747062 2.353981086 -12.79420768 1.37E-28 4.24E-27 54.18347999

NFATC1 -1.118039422 1.420865234 -12.79210623 1.39E-28 4.30E-27 54.16786527

CLIP4 -1.001876616 1.037874515 -12.7781344 1.55E-28 4.76E-27 54.0640588

NRP1 -1.437444775 3.042125958 -12.74163951 2.03E-28 6.23E-27 53.79299837

BMERB1 -1.258615584 2.416371808 -12.71866103 2.41E-28 7.37E-27 53.62239357

LSP1 -1.479889169 3.778043509 -12.70966957 2.58E-28 7.87E-27 53.55564981

CACNA1H -1.471677318 2.478119729 -12.69966222 2.78E-28 8.47E-27 53.48137421

PILRA -1.498639599 2.452236339 -12.69809206 2.81E-28 8.55E-27 53.46972117

CDH5 -1.306793553 3.45447796 -12.69184248 2.95E-28 8.94E-27 53.42334196

ADAMTS14 -1.227373018 2.427878058 -12.68182396 3.17E-28 9.62E-27 53.34900077

ZNF512B -1.107930454 1.826947472 -12.67048671 3.46E-28 1.04E-26 53.26488591

TGFB1 -1.591047386 4.996141315 -12.65186337 3.97E-28 1.20E-26 53.12674057

UBTD1 -1.143732245 3.836727346 -12.64442336 4.20E-28 1.26E-26 53.07156116

SAMD4A -1.022327042 1.920691798 -12.64392966 4.21E-28 1.27E-26 53.0678998

SIGLEC1 -1.40334873 1.53975795 -12.62447024 4.87E-28 1.46E-26 52.92360465

CCL21 -2.834190488 4.36399894 -12.61402653 5.27E-28 1.57E-26 52.84617838

NRROS -1.079453743 2.018226661 -12.59053506 6.28E-28 1.87E-26 52.67206055

DYSF -1.234012721 2.640914322 -12.57188808 7.21E-28 2.14E-26 52.53389012

GPSM1 -1.270855984 2.000839222 -12.55491302 8.19E-28 2.42E-26 52.40813947

SUSD2 -1.271690246 1.691802775 -12.54400316 8.88E-28 2.62E-26 52.32733546

DACT1 -1.586962183 2.714728709 -12.53627815 9.41E-28 2.77E-26 52.27012766

NGFR -1.512369676 1.204930553 -12.52006738 1.06E-27 3.11E-26 52.15009866

RUSC2 -1.145943033 3.262021353 -12.51731577 1.08E-27 3.17E-26 52.12972781

DSE -1.145587478 1.698248055 -12.50886177 1.15E-27 3.37E-26 52.06714557

EVL -1.190310663 2.46497473 -12.49902661 1.24E-27 3.62E-26 51.9943487

KIRREL1 -1.380404755 3.512348324 -12.4756579 1.48E-27 4.29E-26 51.82142223

CSF1 -1.236440471 3.493591008 -12.45260943 1.76E-27 5.09E-26 51.65092352

LAIR1 -1.316217161 2.185204182 -12.44293642 1.89E-27 5.45E-26 51.57938575

PPP1R3C -1.292121162 1.418970204 -12.44183046 1.90E-27 5.48E-26 51.57120719

UCHL1 -1.82648993 2.134525582 -12.41363774 2.35E-27 6.74E-26 51.36276736

IL1R1 -1.440400275 3.305975261 -12.40265449 2.55E-27 7.30E-26 51.28158785

MATN3 -1.532947899 1.572547201 -12.37943844 3.03E-27 8.65E-26 51.11003788

MMP9 -2.359755056 5.075642256 -12.37562334 3.11E-27 8.85E-26 51.08185293

WWTR1 -1.443160613 2.940842978 -12.37237686 3.19E-27 9.03E-26 51.05787

IGFBP6 -1.706868354 3.840910918 -12.36381991 3.40E-27 9.61E-26 50.99466257

TREM2 -2.02391345 3.246033613 -12.35595858 3.61E-27 1.02E-25 50.93660086

ZEB1 -1.302033684 2.292854193 -12.3492794 3.79E-27 1.06E-25 50.88727583

TRPV4 -1.084457193 1.328642741 -12.32101954 4.67E-27 1.31E-25 50.67863663

MFAP4 -2.144453037 4.944867572 -12.31321386 4.95E-27 1.38E-25 50.62102464

ANKRD65 -1.130829318 1.301784574 -12.30962043 5.09E-27 1.42E-25 50.59450471

COL14A1 -1.830790384 3.163882015 -12.29009564 5.88E-27 1.63E-25 50.4504361

TNXB -1.163479151 0.975315323 -12.28423511 6.14E-27 1.70E-25 50.40720152

HSPG2 -1.626112943 5.055233219 -12.2831993 6.19E-27 1.71E-25 50.39956053

ANOS1 -1.459507439 1.768377015 -12.27759169 6.46E-27 1.78E-25 50.35819625

PALLD -1.338482224 4.761791213 -12.26935135 6.86E-27 1.89E-25 50.29741863

SGCE -1.381522028 2.609938359 -12.25537845 7.61E-27 2.09E-25 50.19437844

CSPG4 -1.270712148 2.798632854 -12.24590334 8.17E-27 2.23E-25 50.12451958

NIBAN1 -1.37505461 2.094011356 -12.23188426 9.07E-27 2.47E-25 50.02117853

LIMS2 -1.383699197 2.497493087 -12.19462593 1.20E-26 3.21E-25 49.74664699

DOK3 -1.058415868 2.126357945 -12.18577335 1.28E-26 3.42E-25 49.68144344

CTSK -1.763874838 6.409439598 -12.18486671 1.29E-26 3.44E-25 49.67476617

PLPPR2 -1.103215861 3.555974781 -12.16904694 1.45E-26 3.85E-25 49.55827195

BICC1 -1.360813512 2.097276293 -12.16108705 1.53E-26 4.08E-25 49.49966848

PDPN -1.594736561 3.994959406 -12.14753041 1.70E-26 4.49E-25 49.39987813

ICAM1 -1.332438132 4.693061255 -12.13360657 1.88E-26 4.96E-25 49.29740896

CRMP1 -1.059015872 1.731068471 -12.13261808 1.89E-26 4.99E-25 49.29013537

SDC2 -1.505766302 4.212550573 -12.12922583 1.94E-26 5.10E-25 49.26517509

C1orf162 -1.182248215 2.328977859 -12.10562747 2.31E-26 6.06E-25 49.0915783

LMCD1 -1.107651794 2.610462028 -12.1000991 2.41E-26 6.30E-25 49.05092022

GUCY1B1 -1.342921008 2.728485295 -12.09691886 2.47E-26 6.44E-25 49.02753319

DCN -2.050564334 4.668444948 -12.09396488 2.52E-26 6.57E-25 49.00581113

FCGR2B -1.043019409 1.01858095 -12.0699665 3.01E-26 7.82E-25 48.82938162

KIF26B -1.489234707 1.709571832 -12.06718256 3.07E-26 7.96E-25 48.80891969

CAVIN3 -1.605280099 4.444164707 -12.05107854 3.46E-26 8.94E-25 48.69057541

ERG -1.01616922 1.63981143 -12.0280991 4.11E-26 1.05E-24 48.52176477

ARMCX2 -1.392173634 2.551457863 -12.01453055 4.54E-26 1.16E-24 48.42212097

SNAI1 -1.146053435 3.472360127 -12.00725683 4.79E-26 1.23E-24 48.3687149

MYH10 -1.473947021 2.822712509 -11.98497232 5.65E-26 1.44E-24 48.20513893

FHL3 -1.281906316 4.24683165 -11.98412562 5.68E-26 1.45E-24 48.19892522

SHANK3 -1.114924188 2.667773874 -11.96701806 6.45E-26 1.63E-24 48.0733975

LTBP1 -1.492718589 4.32837593 -11.9597548 6.81E-26 1.72E-24 48.02011501

DCHS1 -1.282301314 2.867918169 -11.95525151 7.04E-26 1.77E-24 47.98708296

PLVAP -1.197709264 6.578026074 -11.95490047 7.06E-26 1.78E-24 47.98450819

FILIP1L -1.409379251 4.314640666 -11.94747449 7.45E-26 1.87E-24 47.93004438

GNB4 -1.241352911 2.20987164 -11.93798691 7.99E-26 2.00E-24 47.86047148

TCEAL3 -1.20524602 2.834998316 -11.93702908 8.05E-26 2.01E-24 47.85344837

ARMCX1 -1.329213907 2.295287743 -11.92759098 8.63E-26 2.16E-24 47.7842519

CHST15 -1.350785589 2.611302469 -11.92346872 8.90E-26 2.22E-24 47.75403295

PECAM1 -1.310069139 4.668845115 -11.9217898 9.01E-26 2.24E-24 47.74172605

PDGFC -1.38542099 2.49844292 -11.91874498 9.22E-26 2.29E-24 47.71940775

CCN1 -1.788387316 5.890175553 -11.91131994 9.74E-26 2.42E-24 47.66498811

ITGA7 -1.172891438 2.638488006 -11.90921093 9.89E-26 2.45E-24 47.64953216

IL18BP -1.088221237 3.001060758 -11.90338416 1.03E-25 2.55E-24 47.60683371

HTR2B -1.069277853 0.702961928 -11.9015747 1.05E-25 2.58E-24 47.59357494

KLHDC8B -1.190394301 3.43036382 -11.89538203 1.10E-25 2.70E-24 47.548202

LUM -2.180855308 7.306096939 -11.89274846 1.12E-25 2.75E-24 47.52890782

ADAMTS1 -1.405520434 2.964327122 -11.8919347 1.12E-25 2.75E-24 47.52294616

CRIP2 -1.289270087 4.128445896 -11.89186675 1.12E-25 2.75E-24 47.52244839

CTSL -1.39845033 5.856592555 -11.8814978 1.21E-25 2.97E-24 47.44649356

RBMS1 -1.353225371 2.398048849 -11.86398199 1.38E-25 3.36E-24 47.3182211

SPP1 -3.152652066 5.749548759 -11.86049196 1.42E-25 3.44E-24 47.29266796

KCNJ8 -1.197362289 2.270218094 -11.84306517 1.61E-25 3.88E-24 47.16509958

P3H3 -1.389250114 3.346800655 -11.83463983 1.71E-25 4.12E-24 47.10343972

THBD -1.190704613 3.229493407 -11.81701427 1.95E-25 4.67E-24 46.9744823

A2M -1.485773491 6.530776363 -11.78351825 2.50E-25 5.96E-24 46.72953344

RAI14 -1.154922727 3.473805159 -11.77835507 2.60E-25 6.18E-24 46.69179091

PDGFRL -1.468548647 1.498319831 -11.75754041 3.03E-25 7.20E-24 46.53967717

PLEKHG2 -1.024390809 2.757490074 -11.74214108 3.39E-25 8.04E-24 46.4271803

ADAM33 -1.097390452 1.069009766 -11.74036169 3.43E-25 8.12E-24 46.41418358

SGCA -1.247417065 1.075947579 -11.73987958 3.45E-25 8.13E-24 46.41066233

ADAMTSL4 -1.157165457 1.846189012 -11.73792072 3.50E-25 8.24E-24 46.39635541

C4B -1.143512733 1.264348379 -11.73313672 3.62E-25 8.52E-24 46.36141711

VWF -1.519185298 4.496360865 -11.72619345 3.81E-25 8.94E-24 46.31071533

CD37 -1.50630286 2.600409414 -11.72449575 3.86E-25 9.04E-24 46.29831935

SPART -1.237491406 2.007825658 -11.71311433 4.20E-25 9.81E-24 46.21522771

LRRN2 -1.17815571 1.122491711 -11.71018981 4.29E-25 1.00E-23 46.19388

ELN -1.742796106 3.989345499 -11.70659138 4.40E-25 1.03E-23 46.16761484

LAPTM5 -1.652939275 6.159326899 -11.70478909 4.46E-25 1.04E-23 46.15446051

GAP43 -1.119961435 0.702050788 -11.69324149 4.86E-25 1.12E-23 46.07019034

ARHGAP31 -1.086703683 2.397787087 -11.68633065 5.11E-25 1.18E-23 46.01976729

JAM2 -1.098529645 1.456554238 -11.68432826 5.19E-25 1.19E-23 46.00515876

CHRDL1 -2.020104673 1.667824545 -11.6632509 6.05E-25 1.38E-23 45.85142543

MOXD1 -1.458690452 2.292715573 -11.66241297 6.09E-25 1.39E-23 45.84531515

MYO1F -1.0524844 2.464196626 -11.65818675 6.28E-25 1.43E-23 45.81449892

APOC1 -2.041852608 4.764445771 -11.64847411 6.75E-25 1.54E-23 45.74368791

FLNC -1.858524272 3.107417638 -11.64601355 6.87E-25 1.56E-23 45.72575131

MCAM -1.152945819 4.683702205 -11.64423804 6.96E-25 1.58E-23 45.712809

GPR161 -1.006944618 1.632953554 -11.64054059 7.15E-25 1.62E-23 45.68585868

CD93 -1.3614815 4.059377524 -11.6215404 8.22E-25 1.86E-23 45.54740191

LRRC25 -1.335500889 2.503599495 -11.60984711 8.96E-25 2.02E-23 45.46221966

TIMP1 -1.076365462 8.96332219 -11.59339219 1.01E-24 2.27E-23 45.34238691

KIF3C -1.053382246 2.519381387 -11.58891922 1.04E-24 2.34E-23 45.30981996

GNAO1 -1.020690833 0.804081257 -11.56808245 1.22E-24 2.72E-23 45.15815311

GJA4 -1.202263541 3.398664028 -11.55806502 1.31E-24 2.92E-23 45.08526301

CRISPLD1 -1.108013101 1.260944903 -11.53575099 1.54E-24 3.43E-23 44.92295691

HSD11B1 -1.538724703 1.983836728 -11.52717082 1.64E-24 3.65E-23 44.86056859

IL4I1 -1.584418141 2.999097986 -11.51517623 1.79E-24 3.98E-23 44.77337343

DLC1 -1.048450614 2.26411307 -11.51265912 1.83E-24 4.04E-23 44.75507818

CADM3 -1.080508799 0.618226904 -11.50741325 1.90E-24 4.19E-23 44.7169527

CALB2 -2.004430508 1.80057165 -11.50489411 1.93E-24 4.26E-23 44.6986459

FGF7 -1.262481722 1.312642464 -11.49236062 2.12E-24 4.66E-23 44.60757959

TCF4 -1.0460775 1.857686543 -11.45391554 2.81E-24 6.15E-23 44.32840623

HS3ST2 -1.033473438 0.828006365 -11.44878407 2.92E-24 6.37E-23 44.29116221

TREM1 -1.340054887 1.423458026 -11.42779304 3.40E-24 7.41E-23 44.13885593

LTBP3 -1.359589905 3.884078468 -11.396112 4.29E-24 9.31E-23 43.90912672

HOMER3 -1.26967961 3.16623918 -11.38644146 4.60E-24 9.95E-23 43.8390367

TNFSF12 -1.167968446 3.951706266 -11.38250376 4.74E-24 1.02E-22 43.8105017

THEMIS2 -1.209209656 3.174055512 -11.37969075 4.83E-24 1.04E-22 43.79011852

APLNR -1.450535637 3.703222647 -11.36602917 5.34E-24 1.15E-22 43.69114566

FZD2 -1.227875386 2.467047176 -11.35706611 5.70E-24 1.22E-22 43.6262293

WAS -1.259112949 2.896001688 -11.32787303 7.06E-24 1.50E-22 43.41489087

RARRES2 -1.529092013 5.66026122 -11.30808743 8.15E-24 1.73E-22 43.2717413

C11orf96 -1.507956418 4.327903036 -11.25905629 1.17E-23 2.45E-22 42.91729849

CCL18 -2.763079315 4.056816241 -11.24191334 1.32E-23 2.77E-22 42.79347493

SMIM10 -1.208952708 1.893781035 -11.24115859 1.33E-23 2.78E-22 42.78802459

MAPK11 -1.089015588 2.339082427 -11.21164056 1.65E-23 3.42E-22 42.57494481

WIPF1 -1.243669046 3.172201283 -11.2080538 1.69E-23 3.51E-22 42.54906413

FBXO17 -1.185896357 1.180716007 -11.20252418 1.76E-23 3.65E-22 42.50916916

WNT9A -1.01848282 1.140833027 -11.19842946 1.81E-23 3.75E-22 42.47963026

CHRDL2 -2.212046717 1.972584786 -11.19090758 1.91E-23 3.95E-22 42.42537627

C7 -1.913176297 1.767956921 -11.19035679 1.92E-23 3.96E-22 42.42140391

ZCCHC24 -1.465839664 3.522759349 -11.19035203 1.92E-23 3.96E-22 42.42136964

ADAM19 -1.291412332 3.83813932 -11.17087456 2.21E-23 4.54E-22 42.28093259

KLHL5 -1.185470687 2.846538856 -11.16404888 2.33E-23 4.77E-22 42.23173451

COL4A1 -1.397806225 6.754352839 -11.14756547 2.62E-23 5.36E-22 42.11296138

PRAF2 -1.070981669 4.19837429 -11.11880643 3.23E-23 6.56E-22 41.90585664

CYTH4 -1.148984434 2.143323834 -11.11537263 3.31E-23 6.69E-22 41.88113901

CD84 -1.182292815 1.513545772 -11.11514051 3.32E-23 6.69E-22 41.87946816

MXRA7 -1.226038445 3.47663778 -11.10452212 3.58E-23 7.21E-22 41.803048

LILRB1 -1.067126853 1.481488329 -11.10266405 3.63E-23 7.30E-22 41.78967767

NFAM1 -1.00585563 2.056791747 -11.09795745 3.76E-23 7.53E-22 41.75581299

SYNPO2 -1.793884563 2.249837334 -11.08627355 4.09E-23 8.15E-22 41.67176369

CCR1 -1.358174431 2.749740275 -11.07220009 4.53E-23 9.00E-22 41.57055952

OLFML2A -1.042425114 2.795229912 -11.06840955 4.66E-23 9.24E-22 41.54330778

GADD45B -1.112192696 4.836076793 -11.06675733 4.71E-23 9.34E-22 41.53143016

COL7A1 -1.532007193 2.869606004 -11.06006358 4.95E-23 9.76E-22 41.48331484

SAMD11 -1.088266021 1.731673158 -11.0560459 5.09E-23 1.00E-21 41.45443956

LYVE1 -1.214594751 1.256605635 -11.05595443 5.10E-23 1.00E-21 41.45378218

S1PR1 -1.21477547 3.215602012 -11.04717291 5.43E-23 1.06E-21 41.39067987

PLXND1 -1.190318155 4.177029235 -11.00488306 7.38E-23 1.44E-21 41.08700339

VAT1 -1.017799691 6.153931265 -10.99952095 7.67E-23 1.49E-21 41.04852394

P2RY6 -1.02541906 1.720276151 -10.98462807 8.54E-23 1.66E-21 40.94167984

PREX1 -1.072867943 3.479373801 -10.97583675 9.11E-23 1.77E-21 40.87863001

ADGRL2 -1.110062747 1.955385746 -10.97412417 9.22E-23 1.79E-21 40.86634946

WNT2 -1.389093194 2.946238568 -10.96179627 1.01E-22 1.95E-21 40.7779659

C1QTNF3 -1.089498714 1.97313251 -10.94305195 1.15E-22 2.21E-21 40.6436386

PHLDB2 -1.192623091 1.583933965 -10.92144584 1.35E-22 2.58E-21 40.48889048

CD163 -1.782040552 2.895421002 -10.91575905 1.41E-22 2.68E-21 40.44817593

CASQ2 -1.367724022 0.753203121 -10.89034557 1.69E-22 3.20E-21 40.26630872

CD14 -1.472952837 5.784734395 -10.88181108 1.80E-22 3.40E-21 40.20526271

TPST1 -1.197815888 2.588524645 -10.88103222 1.81E-22 3.41E-21 40.19969238

RGS19 -1.237820215 3.697323709 -10.8731997 1.91E-22 3.60E-21 40.14368174

PLAU -1.414766152 5.816005218 -10.87261619 1.92E-22 3.61E-21 40.13950955

HCK -1.417222161 3.390478231 -10.8692421 1.97E-22 3.68E-21 40.11538564

TNFRSF4 -1.065450995 2.601011521 -10.83716784 2.48E-22 4.61E-21 39.88617973

ACP5 -1.413582672 5.204738046 -10.83646152 2.49E-22 4.62E-21 39.88113467

CAV1 -1.288660088 4.458808816 -10.83267571 2.56E-22 4.74E-21 39.8540955

SCARA3 -1.303676468 2.456305446 -10.83194579 2.57E-22 4.76E-21 39.8488826

GPR84 -1.022218413 1.263177937 -10.81824335 2.84E-22 5.22E-21 39.75104359

TEK -1.043770231 1.961721824 -10.81776009 2.85E-22 5.24E-21 39.74759366

SYNM -1.757364782 2.148953335 -10.81505207 2.91E-22 5.33E-21 39.7282626

UBE2E2 -1.254361809 2.330875857 -10.8149988 2.91E-22 5.33E-21 39.72788235

STAB1 -1.258638606 3.288131697 -10.81136534 2.98E-22 5.46E-21 39.70194757

GEM -1.276096847 4.323062256 -10.77750537 3.81E-22 6.96E-21 39.46039636

FST -1.16952588 1.441042366 -10.77120259 3.98E-22 7.25E-21 39.41546015

SEMA3G -1.144888102 1.800978381 -10.76635627 4.12E-22 7.49E-21 39.38091361

LILRB2 -1.192390259 1.967492715 -10.76134485 4.28E-22 7.76E-21 39.34519549

FCGR3A -1.916842723 4.100826195 -10.75510485 4.47E-22 8.10E-21 39.30072826

CSF1R -1.566712571 3.693843569 -10.74906816 4.67E-22 8.45E-21 39.25771783

PLXNC1 -1.130059065 1.980979343 -10.74788235 4.71E-22 8.51E-21 39.24927002

DENND5A -1.006714594 3.136325058 -10.74465183 4.82E-22 8.70E-21 39.22625712

HSPB6 -2.141100592 2.872302779 -10.71502773 5.96E-22 1.07E-20 39.01533155

LIMD2 -1.086033174 2.893063058 -10.70326936 6.49E-22 1.16E-20 38.93166369

GPRC5B -1.064741738 2.141340011 -10.69913198 6.69E-22 1.20E-20 38.90223091

DPYD -1.3012161 1.887299221 -10.67272449 8.08E-22 1.44E-20 38.71445922

QKI -1.101317185 2.148485545 -10.66875055 8.31E-22 1.48E-20 38.68621552

FHL1 -1.44935319 3.116079217 -10.60814984 1.28E-21 2.25E-20 38.25594481

CXCL12 -1.414447602 3.159555502 -10.59482626 1.41E-21 2.46E-20 38.16145582

ACTG2 -2.243506606 4.97863394 -10.59204937 1.44E-21 2.51E-20 38.14176755

SSPN -1.101034603 2.078452705 -10.58906673 1.47E-21 2.56E-20 38.12062239

EPYC -1.64621521 0.953475352 -10.58235166 1.54E-21 2.68E-20 38.07302383

TYMP -1.440394793 5.201985276 -10.57313168 1.65E-21 2.86E-20 38.00768625

IGFBP3 -1.304173509 6.01214029 -10.5645101 1.75E-21 3.03E-20 37.94660668

PLIN4 -1.845016111 1.185760485 -10.55243576 1.91E-21 3.30E-20 37.86109436

ALOX5AP -1.406509381 3.99126149 -10.54074888 2.08E-21 3.57E-20 37.7783577

HCST -1.338858988 2.950031499 -10.53258892 2.20E-21 3.77E-20 37.72060825

THBS1 -1.504178587 6.00055667 -10.50880666 2.61E-21 4.45E-20 37.55238406

FGD5 -1.171731279 2.436693521 -10.48504339 3.09E-21 5.24E-20 37.38442456

HAVCR2 -1.27407302 2.493644956 -10.48465847 3.10E-21 5.25E-20 37.381705

IBSP -1.380087944 1.076601517 -10.48123753 3.18E-21 5.37E-20 37.35753682

GPX8 -1.240345141 3.329812505 -10.47982606 3.21E-21 5.42E-20 37.34756587

STC1 -1.309342539 3.13114646 -10.46489274 3.57E-21 6.00E-20 37.24210205

ARHGAP23 -1.222267341 2.897087496 -10.44515412 4.11E-21 6.87E-20 37.10278174

DOCK2 -1.05628138 1.598399003 -10.43351794 4.46E-21 7.45E-20 37.02069346

KCNMA1 -1.156597782 1.143920576 -10.41588819 5.06E-21 8.42E-20 36.89638411

SYNPO -1.004850659 4.117764709 -10.37538795 6.75E-21 1.11E-19 36.6110925

NCF2 -1.460679455 3.308499029 -10.37036915 6.99E-21 1.15E-19 36.57576644

L1CAM -1.91348295 1.770740066 -10.3573076 7.67E-21 1.26E-19 36.48385803

RASSF2 -1.212706451 2.593778979 -10.35495703 7.80E-21 1.28E-19 36.4673224

ALPL -1.104236681 2.151786428 -10.35350978 7.88E-21 1.29E-19 36.45714208

RAMP2 -1.08917296 4.287778824 -10.345781 8.33E-21 1.37E-19 36.40278454

ADGRF5 -1.065469412 2.773611456 -10.30338568 1.12E-20 1.83E-19 36.10487111

DIP2C -1.001095077 2.552238127 -10.29669728 1.18E-20 1.92E-19 36.05791162

GPC1 -1.2966157 4.053570154 -10.28999011 1.24E-20 2.01E-19 36.01083147

PDCD1LG2 -1.044860978 1.365599753 -10.28882944 1.25E-20 2.02E-19 36.00268543

DTX3 -1.123483422 2.564671429 -10.283289 1.30E-20 2.10E-19 35.9638049

RAMP3 -1.010548169 4.223804537 -10.27723304 1.35E-20 2.19E-19 35.92131536

AHNAK2 -1.560423164 2.067224896 -10.24173414 1.74E-20 2.80E-19 35.67243277

GRP -1.624673104 1.917353431 -10.23926456 1.77E-20 2.84E-19 35.65513026

C3AR1 -1.486923708 3.121370531 -10.21974142 2.03E-20 3.24E-19 35.51839988

FPR3 -1.620226449 3.351354944 -10.20690503 2.23E-20 3.55E-19 35.42855239

FCGR2A -1.288616006 3.272104976 -10.19993571 2.34E-20 3.72E-19 35.37978856

MSR1 -1.474387375 2.166227322 -10.18931158 2.52E-20 4.00E-19 35.30547588

MCEMP1 -1.27501778 1.159112205 -10.18159926 2.66E-20 4.21E-19 35.25154844

GIMAP8 -1.010215267 2.17196218 -10.16413311 3.01E-20 4.76E-19 35.12947456

DES -3.09681336 4.910262402 -10.16296441 3.04E-20 4.79E-19 35.1213091

FOXP3 -1.033402425 2.123635087 -10.12922025 3.85E-20 6.07E-19 34.88569706

FZD8 -1.05561061 3.067122597 -10.1201122 4.11E-20 6.47E-19 34.82215221

OBSL1 -1.488302075 2.297628654 -10.11594838 4.23E-20 6.64E-19 34.79310932

DKK2 -1.165869439 1.221509589 -10.08607722 5.22E-20 8.17E-19 34.58488832

APBB1IP -1.102232509 2.056241138 -10.08104824 5.41E-20 8.46E-19 34.54985609

AQP9 -1.559689161 1.791194351 -10.06728088 5.96E-20 9.31E-19 34.45398545

VSIG4 -1.738074181 3.226365231 -10.06064025 6.24E-20 9.74E-19 34.40776049

ANXA6 -1.099517099 5.822987737 -10.06008951 6.26E-20 9.76E-19 34.40392733

CLEC2B -1.072858441 2.090632502 -10.04182017 7.12E-20 1.11E-18 34.27681841

ZNF385A -1.285914705 3.439050325 -10.03330066 7.56E-20 1.18E-18 34.21757418

ABI3 -1.003975676 3.256880658 -10.01056967 8.87E-20 1.37E-18 34.05959839

CYBRD1 -1.518157359 4.088281397 -10.00103752 9.48E-20 1.46E-18 33.99339302

TYROBP -1.608887816 5.902185288 -9.992077722 1.01E-19 1.55E-18 33.93118503

FABP4 -2.113446674 1.513903881 -9.989230867 1.03E-19 1.58E-18 33.9114238

TCEA2 -1.201506673 2.222843735 -9.979170844 1.11E-19 1.69E-18 33.8416104

SFRP1 -1.354332503 0.918774768 -9.964111996 1.23E-19 1.87E-18 33.73715776

SCG2 -1.432651576 1.707383236 -9.948210506 1.37E-19 2.09E-18 33.62692701

HCLS1 -1.189128168 3.392456636 -9.939953857 1.45E-19 2.21E-18 33.56971819

DOK2 -1.185172733 2.7951853 -9.889920511 2.06E-19 3.11E-18 33.22344555

OMD -1.089600844 0.682943839 -9.888764551 2.08E-19 3.13E-18 33.21545348

NCKAP1L -1.127257231 2.17380715 -9.880765405 2.20E-19 3.31E-18 33.1601592

CMKLR1 -1.131008889 1.969270243 -9.878941187 2.23E-19 3.34E-18 33.14755174

ARHGAP30 -1.192098568 2.901705869 -9.871223886 2.35E-19 3.52E-18 33.09422644

RERG -1.121131079 1.344451216 -9.865725746 2.44E-19 3.64E-18 33.05624528

INMT -1.029350853 2.200072491 -9.863469858 2.48E-19 3.69E-18 33.04066404

MYADM -1.173822469 6.349299785 -9.854958346 2.63E-19 3.90E-18 32.98188847

SLA -1.075849713 2.11171335 -9.846978217 2.78E-19 4.11E-18 32.9268007

PLAT -1.208622383 4.379262732 -9.829890917 3.14E-19 4.61E-18 32.80890505

RSPO3 -1.389395492 1.454353592 -9.810273888 3.60E-19 5.27E-18 32.67365652

IL10RA -1.187944323 2.52300844 -9.787053941 4.23E-19 6.19E-18 32.51370852

ATP2B4 -1.116370861 3.894437225 -9.777879692 4.50E-19 6.57E-18 32.45055496

BEX4 -1.453327414 3.591520051 -9.760828755 5.07E-19 7.36E-18 32.33324381

F2R -1.096928874 4.666346928 -9.760627283 5.08E-19 7.37E-18 32.33185817

VCAM1 -1.194956486 2.662963553 -9.737125823 5.98E-19 8.61E-18 32.17030513

GLIPR2 -1.066338399 3.927521052 -9.737048452 5.98E-19 8.61E-18 32.16977353

FYN -1.092510681 3.09064931 -9.731752498 6.21E-19 8.91E-18 32.13339031

PIK3CD -1.038184964 2.214394796 -9.72323125 6.58E-19 9.43E-18 32.07486633

TSC22D3 -1.037121403 4.707998221 -9.718212785 6.82E-19 9.75E-18 32.04040931

ETS1 -1.167144845 4.32432555 -9.709206107 7.26E-19 1.04E-17 31.97858736

STX11 -1.056038867 1.850508417 -9.68835668 8.39E-19 1.19E-17 31.83556716

ALDH1A3 -1.169390369 2.599769026 -9.674534747 9.23E-19 1.31E-17 31.74082323

DIO2 -1.133621768 2.078633494 -9.670839041 9.47E-19 1.34E-17 31.71550011

ADAM8 -1.316087451 3.164551337 -9.667456015 9.69E-19 1.37E-17 31.69232299

IGFBP7 -1.251448887 8.464320822 -9.657482829 1.04E-18 1.46E-17 31.62401626

TSPAN11 -1.062076641 1.973159759 -9.654546755 1.06E-18 1.49E-17 31.60391256

AQP1 -1.462633576 5.246259022 -9.642277567 1.15E-18 1.62E-17 31.51993128

CSF2RB -1.315843405 2.539274966 -9.641295059 1.16E-18 1.63E-17 31.51320804

SDS -1.169256168 2.573979029 -9.615849159 1.38E-18 1.94E-17 31.33918313

LSAMP -1.008469999 1.54138755 -9.579881706 1.77E-18 2.46E-17 31.09353051

NPTXR -1.331114617 1.566550075 -9.564421701 1.97E-18 2.73E-17 30.98806024

SLFN11 -1.217518373 2.426799946 -9.541068181 2.32E-18 3.20E-17 30.82887629

EVA1B -1.032361411 4.192940049 -9.540020412 2.34E-18 3.22E-17 30.82173829

RASSF8 -1.059941205 1.514763522 -9.529998201 2.50E-18 3.44E-17 30.75347813

VMO1 -1.104104499 2.397119226 -9.513920234 2.80E-18 3.83E-17 30.64403689

ACKR1 -1.736527564 2.447303003 -9.471970115 3.73E-18 5.09E-17 30.35885964

FCN1 -1.003789756 1.05999585 -9.460012084 4.05E-18 5.51E-17 30.27766832

GJA1 -1.203293461 4.38679288 -9.45109204 4.30E-18 5.85E-17 30.21713288

OGN -1.410797499 1.074807536 -9.449256173 4.36E-18 5.92E-17 30.20467694

PHLDA3 -1.43115227 4.467599121 -9.442669583 4.56E-18 6.18E-17 30.15999702

CLEC4E -1.099339628 1.173760426 -9.413245524 5.58E-18 7.53E-17 29.9605654

CILP2 -1.082227981 0.982657595 -9.400086886 6.10E-18 8.23E-17 29.87146612

CBX6 -1.246839244 3.267189303 -9.392764857 6.42E-18 8.63E-17 29.8219111

FCER1G -1.462938153 5.368979168 -9.382794485 6.87E-18 9.20E-17 29.75445949

CD86 -1.160844684 2.284304973 -9.377144709 7.14E-18 9.55E-17 29.71625157

SIGLEC14 -1.07696501 1.353727887 -9.372892802 7.35E-18 9.82E-17 29.68750373

MDFIC -1.169800613 2.518648812 -9.367044953 7.65E-18 1.02E-16 29.64797484

CHIT1 -1.14704763 0.868255438 -9.366387282 7.69E-18 1.02E-16 29.64352996

FBXO32 -1.156331713 3.657853397 -9.339057614 9.26E-18 1.23E-16 29.45894356

NID1 -1.157855088 4.960259751 -9.319568736 1.06E-17 1.40E-16 29.32746036

CD300LF -1.065674611 1.946588271 -9.302638083 1.19E-17 1.56E-16 29.21333559

MAGEH1 -1.165257832 3.594518761 -9.27846994 1.40E-17 1.84E-16 29.05058554

SIGLEC10 -1.172305796 2.220860242 -9.270784112 1.48E-17 1.93E-16 28.99886836

GPX3 -1.394463912 4.242720738 -9.242784035 1.78E-17 2.33E-16 28.81062161

CLU -1.567039765 4.18711193 -9.232688319 1.91E-17 2.49E-16 28.7428103

CCL2 -1.482024588 3.808198848 -9.221376806 2.06E-17 2.68E-16 28.66687251

SIRPA -1.410302348 4.008161611 -9.207783754 2.26E-17 2.93E-16 28.57567385

DBN1 -1.187808117 4.717376855 -9.20474249 2.31E-17 2.99E-16 28.55527773

RETN -1.080890013 0.822113353 -9.201204303 2.37E-17 3.05E-16 28.53155288

OLFM2 -1.293716576 2.454156109 -9.194652609 2.47E-17 3.18E-16 28.4876323

CSF3R -1.101648717 1.598648535 -9.169428881 2.93E-17 3.75E-16 28.3186733

SOCS3 -1.188375446 5.608052616 -9.168201667 2.96E-17 3.78E-16 28.31045832

ADA2 -1.434118458 3.375333569 -9.167139063 2.98E-17 3.80E-16 28.30334565

C1QC -1.555159303 6.868500714 -9.158973538 3.15E-17 4.01E-16 28.24870132

FSCN1 -1.526076751 5.052223867 -9.131270091 3.80E-17 4.83E-16 28.06347439

C1QA -1.427973596 6.657063306 -9.111309006 4.35E-17 5.50E-16 27.93017371

TMEM47 -1.274594961 2.916817632 -9.106326128 4.50E-17 5.68E-16 27.89691895

RUNX3 -1.320398148 3.094919975 -9.101372263 4.65E-17 5.87E-16 27.86386618

ABLIM3 -1.095125635 1.828882218 -9.098533757 4.74E-17 5.97E-16 27.84493109

ITGAL -1.11844795 2.354311882 -9.083491873 5.24E-17 6.59E-16 27.74463553

PTAFR -1.262535022 2.821754732 -9.068805807 5.79E-17 7.25E-16 27.64678698

HLA-DQA1 -1.510518239 3.847912784 -9.053770784 6.41E-17 7.99E-16 27.54668997

CLDN5 -1.222707686 2.510244165 -9.044339247 6.83E-17 8.51E-16 27.48393831

LRP1 -1.037559396 5.568644643 -9.037822323 7.13E-17 8.88E-16 27.4405966

PCDHGC3 -1.21328972 2.163067681 -9.018991285 8.10E-17 1.01E-15 27.31544061

TMEM45A -1.02778646 1.73687111 -9.003469131 8.99E-17 1.11E-15 27.21236874

SELPLG -1.182072434 3.806657288 -8.994969015 9.51E-17 1.18E-15 27.15596086

SORBS1 -1.302207931 3.472153314 -8.970009879 1.12E-16 1.39E-15 26.9904746

C1QTNF1 -1.007789919 4.571514096 -8.95250763 1.26E-16 1.56E-15 26.87455993

CTSF -1.234055232 3.673554278 -8.950783746 1.28E-16 1.57E-15 26.86314874

NR3C1 -1.023056809 2.162405894 -8.940049023 1.37E-16 1.68E-15 26.79211423

RTL8C -1.332425557 6.208804976 -8.93144774 1.46E-16 1.78E-15 26.73522664

SMARCA1 -1.209187801 2.563206619 -8.913963775 1.64E-16 1.99E-15 26.61967113

CD53 -1.404593977 4.652131725 -8.888447014 1.94E-16 2.35E-15 26.45122025

LCP2 -1.041252249 2.713865629 -8.887053018 1.96E-16 2.37E-15 26.44202437

CAVIN2 -1.170488473 2.411093197 -8.878534782 2.07E-16 2.50E-15 26.38584661

SELP -1.185598953 1.579836662 -8.870265165 2.19E-16 2.64E-15 26.33133338

MRC1 -1.45965448 2.489179603 -8.857882501 2.38E-16 2.85E-15 26.24975295

CD109 -1.317873623 1.740947741 -8.83481041 2.78E-16 3.32E-15 26.09789511

F13A1 -1.506153398 2.504148389 -8.823006781 3.00E-16 3.58E-15 26.02027949

GIMAP4 -1.086476474 3.683844309 -8.819068996 3.08E-16 3.67E-15 25.99439755

CTF1 -1.033771697 1.512560198 -8.809554401 3.28E-16 3.91E-15 25.93188412

GPX7 -1.15047316 3.217940562 -8.80467059 3.39E-16 4.03E-15 25.89980899

HLA-DPB1 -1.484777002 6.212546103 -8.80142979 3.47E-16 4.11E-15 25.87852937

HAND1 -1.173376139 0.663399717 -8.788180999 3.79E-16 4.48E-15 25.79157559

VAMP5 -1.108347364 5.249243872 -8.780102618 3.99E-16 4.71E-15 25.73858751

LY86 -1.076485783 2.362044144 -8.776051712 4.10E-16 4.83E-15 25.71202565

SNCG -1.168952912 2.199218986 -8.771646491 4.22E-16 4.96E-15 25.68314737

TSPAN18 -1.182610359 2.552913485 -8.766717024 4.37E-16 5.11E-15 25.65084088

HLA-DOA -1.422001564 2.78382998 -8.751058947 4.84E-16 5.65E-15 25.54828116

PMEPA1 -1.363805566 6.472325664 -8.750128184 4.87E-16 5.68E-15 25.54218755

CD4 -1.231168857 4.014661284 -8.747932115 4.94E-16 5.76E-15 25.52781138

CLEC7A -1.028746251 1.847297961 -8.678519789 7.83E-16 8.99E-15 25.07434008

CD300A -1.102599705 2.875660516 -8.671636534 8.19E-16 9.38E-15 25.02946992

SLC37A2 -1.233743297 1.979328385 -8.671267739 8.21E-16 9.40E-15 25.02706635

RGS4 -1.047288268 1.123092682 -8.661579097 8.75E-16 1.00E-14 24.96394016

ROBO1 -1.153302815 2.591465116 -8.650293695 9.43E-16 1.08E-14 24.89045496

ANGPTL4 -1.248417242 3.026329463 -8.612332101 1.21E-15 1.37E-14 24.64362129

LAMA2 -1.152010116 1.875072693 -8.591331585 1.39E-15 1.57E-14 24.50730777

MNDA -1.231525282 2.527398063 -8.572215598 1.58E-15 1.77E-14 24.38337362

MMP13 -1.171506524 0.9145078 -8.53655625 1.99E-15 2.22E-14 24.15256088

SPOCK2 -1.092130581 3.067171094 -8.535938694 2.00E-15 2.23E-14 24.14856795

CCL19 -1.883550178 2.668789264 -8.523458785 2.17E-15 2.40E-14 24.06790847

GIMAP6 -1.005312655 2.780362348 -8.518560902 2.24E-15 2.47E-14 24.03626929

SULF2 -1.198390608 6.055250008 -8.505474333 2.44E-15 2.69E-14 23.95177901

PDZRN3 -1.081322087 2.7296145 -8.481932191 2.85E-15 3.12E-14 23.79995352

PI16 -1.009983157 0.755118269 -8.462939081 3.22E-15 3.52E-14 23.67762384

BCL2A1 -1.289350099 3.069890518 -8.458289891 3.32E-15 3.63E-14 23.64770129

TRIB2 -1.060813901 3.545597415 -8.448998116 3.53E-15 3.84E-14 23.58792428

PLA2G7 -1.140843145 3.242530076 -8.431894432 3.94E-15 4.27E-14 23.47798014

MEGF6 -1.22989433 2.810402587 -8.425069557 4.12E-15 4.45E-14 23.43414161

CYBB -1.355161409 3.964532247 -8.405738495 4.67E-15 5.02E-14 23.31007222

C1QB -1.460161009 6.623077588 -8.383505875 5.40E-15 5.77E-14 23.16756496

NES -1.205282979 4.341196648 -8.313487735 8.50E-15 8.93E-14 22.7200626

FPR1 -1.313611107 2.335837336 -8.305640766 8.94E-15 9.37E-14 22.67003484

LTB -1.117598351 3.453828675 -8.298747158 9.35E-15 9.78E-14 22.62610592

CCL3 -1.255855378 2.942132942 -8.298690541 9.35E-15 9.78E-14 22.62574521

RGS16 -1.057600624 4.410017726 -8.283013765 1.03E-14 1.08E-13 22.52591912

MS4A4A -1.230511484 2.600182032 -8.247602313 1.30E-14 1.34E-13 22.30079915

TNFSF13B -1.041794542 2.209871833 -8.246211859 1.31E-14 1.35E-13 22.29197023

CASP5 1.297445636 3.19739491 8.232471899 1.43E-14 1.47E-13 22.2047691

SMO -1.120324372 2.738685216 -8.227689852 1.48E-14 1.51E-13 22.17443805

AMIGO2 -1.212693926 3.783440263 -8.215214373 1.60E-14 1.64E-13 22.0953547

ZNF853 -1.099199825 1.982677048 -8.205479074 1.70E-14 1.74E-13 22.03368668

ECM1 -1.098487468 5.045284425 -8.20241977 1.74E-14 1.77E-13 22.01431576

OLFML3 -1.269051537 4.215679592 -8.194283197 1.83E-14 1.86E-13 21.96281559

SHISA2 -1.029540568 1.894751145 -8.176297052 2.06E-14 2.09E-13 21.84907131

GBP5 -1.132825614 1.81755399 -8.132243873 2.72E-14 2.73E-13 21.57105457

RRAD -1.164801365 2.142685432 -8.114339411 3.06E-14 3.05E-13 21.45829553

CDKN2B -1.12195479 3.197634655 -8.099718578 3.35E-14 3.35E-13 21.36631742

FMOD -1.308457211 5.326883295 -8.097473657 3.40E-14 3.39E-13 21.35220295

KRT17 -1.905223409 3.701019123 -8.096263942 3.43E-14 3.42E-13 21.344598

PGM5 -1.31541162 1.977734698 -8.094762752 3.46E-14 3.45E-13 21.33516156

IFI44L -1.157270249 1.598928714 -8.056356008 4.42E-14 4.38E-13 21.0940662

AKR1B1 -1.157359925 3.954690519 -8.028798026 5.27E-14 5.19E-13 20.92146464

CHI3L1 -1.52174521 4.066794335 -8.008796874 5.98E-14 5.85E-13 20.79639927

LCP1 -1.319255179 4.849346244 -8.007052769 6.05E-14 5.92E-13 20.78550178

SELE -1.002712327 1.425232527 -7.98497803 6.95E-14 6.78E-13 20.6476895

FOXC1 -1.11872671 1.980832085 -7.946822081 8.85E-14 8.57E-13 20.40998587

EDNRA -1.172302633 3.00345709 -7.92220654 1.03E-13 9.98E-13 20.25697664

PLEK -1.282857009 3.345258729 -7.885244097 1.30E-13 1.24E-12 20.02772453

SELENOM -1.034734502 4.755552833 -7.881865335 1.33E-13 1.27E-12 20.00679882

RGS2 -1.172911778 4.299549659 -7.812718069 2.06E-13 1.93E-12 19.57967457

TPSAB1 -1.411075918 3.112776688 -7.804971935 2.16E-13 2.01E-12 19.531961

CCL8 -1.148184622 1.608095108 -7.784030231 2.46E-13 2.29E-12 19.40310372

KLF2 -1.12037156 4.498725594 -7.759297811 2.87E-13 2.66E-12 19.2511793

ENPP2 -1.082215462 2.623241936 -7.692357762 4.35E-13 3.99E-12 18.84139553

SASH3 -1.040707098 3.35911358 -7.691543205 4.37E-13 4.01E-12 18.83642186

APOL3 -1.000611401 3.015044727 -7.687024626 4.50E-13 4.11E-12 18.80883709

CD70 -1.002997257 1.599966881 -7.620021654 6.81E-13 6.14E-12 18.40092016

MS4A7 -1.042491293 2.494048957 -7.616433237 6.96E-13 6.27E-12 18.37913316

TSPAN2 -1.180520706 2.586407428 -7.590128208 8.18E-13 7.36E-12 18.21960855

TPSB2 -1.431556861 2.896203681 -7.547638337 1.06E-12 9.48E-12 17.96262604

PTGDS -1.350825909 3.715871064 -7.536084858 1.14E-12 1.01E-11 17.89289853

BCAM -1.003903444 3.974599694 -7.534330636 1.15E-12 1.02E-11 17.88231705

IFIT1 -1.183620812 2.497659349 -7.529173066 1.19E-12 1.06E-11 17.85121509

SGK2 1.006033402 3.930716596 7.487499983 1.54E-12 1.35E-11 17.60038108

SLC6A6 -1.009739686 5.319413775 -7.473429549 1.67E-12 1.47E-11 17.51587926

CXCR4 -1.156313456 5.086864205 -7.467569531 1.73E-12 1.52E-11 17.48071447

MMP17 -1.001778116 1.495381893 -7.461544947 1.80E-12 1.57E-11 17.44457954

CRABP2 -1.428918115 3.53206987 -7.384845136 2.87E-12 2.47E-11 16.98609025

TGM2 -1.036702451 5.802450353 -7.320497998 4.23E-12 3.61E-11 16.60367913

APOD -1.630124018 3.432130056 -7.304828867 4.65E-12 3.95E-11 16.51087063

PTPRU -1.218384035 2.378694403 -7.30010063 4.79E-12 4.06E-11 16.48288935

ENO2 -1.033002441 3.919200923 -7.299664936 4.80E-12 4.07E-11 16.48031151

RSAD2 -1.114827377 2.408320193 -7.257715554 6.17E-12 5.17E-11 16.23256019

OAS2 -1.276428183 3.716073202 -7.248378893 6.53E-12 5.46E-11 16.17753902

FERMT3 -1.001999827 4.197518038 -7.160912167 1.10E-11 8.99E-11 15.6642498

ISG15 -1.082188668 6.940059814 -7.129990158 1.32E-11 1.07E-10 15.48372608

ST3GAL1 -1.077295358 3.186700931 -7.114389677 1.45E-11 1.17E-10 15.39283763

G0S2 -1.280242576 5.010399127 -7.10022311 1.58E-11 1.27E-10 15.31041261

CD52 -1.083703708 4.605972749 -7.05839424 2.02E-11 1.62E-10 15.06765122

TACR2 -1.069881515 1.572319257 -7.042075151 2.23E-11 1.78E-10 14.97318891

HLA-DQB2 -1.27049657 2.693123467 -7.03383043 2.34E-11 1.86E-10 14.92551797

LY96 -1.078283142 3.04465893 -6.989579996 3.03E-11 2.38E-10 14.67027534

SELL -1.037726553 2.377954704 -6.975358833 3.29E-11 2.57E-10 14.58846612

SRGN -1.172118153 6.241795044 -6.974496014 3.31E-11 2.58E-10 14.58350609

CD40 -1.005766647 3.545120636 -6.968213789 3.43E-11 2.68E-10 14.54740388

SLC43A3 -1.095229194 2.94505557 -6.949981844 3.82E-11 2.97E-10 14.44274915

IFI6 -1.315180002 7.921580019 -6.939251216 4.07E-11 3.15E-10 14.38123645

NAT2 1.104152916 3.412016289 6.900649133 5.09E-11 3.92E-10 14.16046338

MX1 -1.118901295 4.072381166 -6.86081185 6.42E-11 4.89E-10 13.93346913

CD74 -1.089015918 9.469469364 -6.854829225 6.65E-11 5.06E-10 13.89945426

ANO1 -1.202833361 3.599145095 -6.843678374 7.09E-11 5.38E-10 13.83610687

SELENBP1 1.21368903 7.605793342 6.83709319 7.37E-11 5.58E-10 13.79872861

RAMP1 -1.734588295 3.929559966 -6.83489049 7.46E-11 5.64E-10 13.78623111

OSM -1.146116004 2.44981991 -6.827146391 7.80E-11 5.89E-10 13.74231432

CCL4L2 -1.040748674 2.583498427 -6.807721319 8.73E-11 6.56E-10 13.63229908

TNFAIP2 -1.008808265 4.35687183 -6.80542578 8.85E-11 6.65E-10 13.61931179

PLTP -1.172649007 6.345120165 -6.800707316 9.09E-11 6.82E-10 13.59262561

AGR3 1.632456821 5.570696135 6.651410671 2.14E-10 1.56E-09 12.75461792

HLA-DRB5 -1.407289102 6.631648369 -6.609605434 2.71E-10 1.96E-09 12.52220132

EPHB6 -1.056984899 1.190577763 -6.59418726 2.96E-10 2.13E-09 12.43673391

HLA-DPA1 -1.270713141 5.470820234 -6.573599384 3.32E-10 2.39E-09 12.32282038

ADH6 1.024278963 2.449994622 6.557220444 3.64E-10 2.61E-09 12.23236795

C4BPA 1.322614462 2.822258711 6.534190854 4.15E-10 2.95E-09 12.10544742

ALOX5 -1.0671617 3.754651187 -6.528896292 4.27E-10 3.04E-09 12.07631114

SPON1 -1.446731036 3.682568679 -6.523451555 4.41E-10 3.13E-09 12.04636525

FKBP10 -1.130707493 5.820794377 -6.494865863 5.17E-10 3.64E-09 11.88942542

KCTD12 -1.089878808 4.035276939 -6.490935831 5.29E-10 3.72E-09 11.86788588

AKR1B10 1.561048839 3.828285432 6.450293385 6.64E-10 4.62E-09 11.64566027

PTPRC -1.01727486 2.911426751 -6.413246075 8.16E-10 5.61E-09 11.44393127

HLA-DRB1 -1.170875139 8.437865264 -6.384025467 9.59E-10 6.56E-09 11.28538785

CPE -1.302286241 4.432421149 -6.308176135 1.46E-09 9.82E-09 10.87620444

MPEG1 -1.009940374 3.47118462 -6.280093932 1.70E-09 1.14E-08 10.72557862

BST2 -1.278927217 6.883022334 -6.271313533 1.78E-09 1.19E-08 10.67857973

MT2A -1.034694421 6.456362729 -6.204522463 2.57E-09 1.68E-08 10.32258709

GBP4 -1.042665876 3.512530805 -6.1821554 2.90E-09 1.89E-08 10.20397572

ADH1B -1.059511892 1.153228521 -6.094956996 4.64E-09 2.95E-08 9.744487139

LCN2 1.620631527 9.245313754 6.081200088 5.00E-09 3.17E-08 9.672422896

CXCL9 -1.385684947 4.095533896 -6.065782814 5.43E-09 3.44E-08 9.591800201

FADS2 -1.166095462 3.11988259 -6.018655642 6.98E-09 4.36E-08 9.346270519

CCL28 1.089174674 3.78335337 6.008311743 7.38E-09 4.59E-08 9.292564866

CST2 -1.15763878 2.154408628 -5.961567791 9.46E-09 5.81E-08 9.050706565

UBD -1.283220038 4.556906514 -5.95877711 9.60E-09 5.89E-08 9.036310763

VIP -1.306631119 2.155979802 -5.956801198 9.70E-09 5.94E-08 9.026120937

SULT1B1 1.125328207 4.01049662 5.953747649 9.86E-09 6.03E-08 9.01037856

CCL5 -1.039506655 4.600699331 -5.948047129 1.02E-08 6.21E-08 8.981005645

ACHE -1.00281386 2.66813912 -5.940229032 1.06E-08 6.45E-08 8.940754944

IL6 -1.027219412 2.025439604 -5.934004157 1.09E-08 6.65E-08 8.908734443

PHGR1 1.28176415 8.487251933 5.92067518 1.17E-08 7.11E-08 8.840253276

FOLR2 -1.039087135 3.050653022 -5.855802518 1.65E-08 9.84E-08 8.508566468

CCL13 -1.182977997 2.123430084 -5.853333348 1.67E-08 9.96E-08 8.495994981

HLA-DQB1 -1.16609649 4.982545007 -5.802335355 2.18E-08 1.28E-07 8.237220853

HOXD9 -1.023321312 3.113370117 -5.724996496 3.25E-08 1.87E-07 7.847997851

LRRC19 1.006804709 3.815452291 5.639858544 5.03E-08 2.84E-07 7.424045631

DUOXA2 1.692860117 3.804301954 5.635814655 5.13E-08 2.89E-07 7.404027553

HLA-DQA2 -1.346617456 3.160777778 -5.607769637 5.92E-08 3.30E-07 7.26549723

C6orf15 -1.289812173 1.605665809 -5.601667553 6.10E-08 3.40E-07 7.23542474

HLA-DRA -1.085667398 9.154225207 -5.594280099 6.34E-08 3.52E-07 7.199050802

CXCL13 -1.221785359 2.727725925 -5.488834167 1.08E-07 5.83E-07 6.683842763

CXCL10 -1.233203896 4.905133583 -5.475815416 1.15E-07 6.19E-07 6.620752363

S100A8 -1.060943881 3.227929508 -5.45663801 1.26E-07 6.78E-07 6.528025575

STMN3 -1.090530887 4.746069648 -5.354759818 2.09E-07 1.09E-06 6.039627772

CES1 -1.472468359 3.027925937 -5.33024673 2.36E-07 1.22E-06 5.923176988

CD79A -1.196574898 3.359634854 -5.293374265 2.83E-07 1.43E-06 5.74879507

INHBB -1.053427655 2.628849228 -5.274311707 3.10E-07 1.56E-06 5.659012122

CCL20 1.105569941 6.554664022 5.263829351 3.26E-07 1.64E-06 5.609748988

ID1 1.090281335 8.084918358 5.214800978 4.13E-07 2.04E-06 5.38035351

FAM3D 1.04593756 8.137380212 5.181386579 4.85E-07 2.37E-06 5.224979741

KRT6A -1.132030925 1.410589476 -5.092004754 7.43E-07 3.53E-06 4.813243504

FDCSP -1.243361761 1.893826156 -5.061830446 8.57E-07 4.03E-06 4.675530118

ADTRP 1.09682509 2.829489065 4.998974125 1.15E-06 5.31E-06 4.390756628

C10orf99 1.354740571 7.222151714 4.746977316 3.65E-06 1.56E-05 3.277968641

NPTX2 -1.383113347 2.938990653 -4.733319235 3.88E-06 1.65E-05 3.21899597

KRT6B -1.287740406 2.924185545 -4.701523293 4.48E-06 1.89E-05 3.082248153

MMP7 -1.358651131 5.241066577 -4.700377526 4.50E-06 1.90E-05 3.077334583

MMP1 -1.252064312 5.943378659 -4.597467147 7.10E-06 2.89E-05 2.640040569

DUOX2 1.365315876 4.348893051 4.571431812 7.95E-06 3.21E-05 2.530680282

HMGCS2 1.46457127 6.467228502 4.563489109 8.23E-06 3.31E-05 2.497420026

CST1 -1.418528481 5.031964236 -4.546594365 8.86E-06 3.55E-05 2.426832934

IGF2 -1.730983907 4.542399264 -4.202833308 3.79E-05 0.000135046 1.038547017

VSIG2 1.324412499 4.262387893 4.170141623 4.33E-05 0.000152841 0.911353589

KLK10 -1.157599368 3.987881091 -4.156537419 4.58E-05 0.000160846 0.858674651

SPINK4 1.613862203 6.157254811 4.018382834 7.97E-05 0.000267702 0.332112381

REG3A 1.813346179 4.46454938 4.008708381 8.28E-05 0.000276817 0.295815962

REG4 1.775446843 5.742808564 4.000940174 8.54E-05 0.000285135 0.266726301

PIGR 1.658875767 9.43994093 3.99447201 8.76E-05 0.000291437 0.242542294

CEACAM7 1.339382783 5.851887237 3.895669359 0.000128817 0.000417081 -0.1226362

CLCA1 1.698643313 4.765198955 3.801785465 0.000184565 0.000579085 -0.462214265

CLCA4 1.143513323 2.451422946 3.653347396 0.000321386 0.00096112 -0.984191558

UGT2B17 1.306421573 3.606965791 3.636957834 0.000341323 0.001014677 -1.040694628

IGLL5 -1.043894363 4.281606666 -3.590538021 0.000404297 0.001187344 -1.199499011

RETNLB 1.0482772 3.447749303 3.406309321 0.000778514 0.002159539 -1.811717965

FCGBP 1.032555745 5.36268265 3.079667737 0.002327483 0.005800567 -2.825175943

DEFA6 1.271624892 4.512873303 3.050916506 0.002552472 0.006301543 -2.909902829

MUC2 1.050605597 5.409724584 2.869420446 0.004499787 0.01041619 -3.427783783

SLC26A3 1.004622412 5.223770866 2.714335905 0.007150158 0.015711504 -3.846871841

ITLN1 1.092594201 4.228974845 2.646474312 0.008702035 0.018671813 -4.023396469

REG1A 1.239670591 5.60204611 2.602981308 0.009849771 0.020809168 -4.134322851

DEFA5 1.069323896 4.406597632 2.383071166 0.017991759 0.035267214 -4.668564863
