# Supplementary material for: Identification and development of a novel invasion-related gene signature for prognosis prediction in colon adenocarcinoma
Source: Cancer Cell Int. 2021 Feb 12;21:101. doi: 10.1186/s12935-021-01795-1 (PMC7881672; doi:10.1186/s12935-021-01795-1)
Supplement: Supplementary file 4 — Additional file 4: Table S4. Differentially expressed genes between C1 ~ C2 cluster. [file 12935_2021_1795_MOESM4_ESM.docx]

logFC AveExpr t P.Value adj.P.Val B

REG4 1.501662909 5.557802255 3.418065231 0.00074925 0.004715958 -1.101182715

PIGR 1.399523013 9.264836103 3.400361333 0.000796907 0.004967537 -1.157984151

CLCA1 1.335281154 4.523946699 3.048457803 0.002577222 0.013332269 -2.231372351

CEACAM7 1.323701996 5.833226507 4.079642986 6.28E-05 0.000577597 1.207688306

ITLN1 1.297723428 4.351801796 3.189667264 0.001628525 0.009072122 -1.813468512

PRAC1 1.278132988 3.195668727 2.740988493 0.006620571 0.028474332 -3.080782052

MUC2 1.21119097 5.5046073 3.295389502 0.001142811 0.006788151 -1.489306204

SPINK4 1.210895114 5.891465879 3.028909609 0.002742824 0.014002124 -2.287856233

DUOXA2 1.167139201 3.460235678 3.990529853 8.94E-05 0.000783139 0.875956308

HMGCS2 1.161492521 6.265690006 3.402433447 0.000791186 0.004939818 -1.151349668

SLC26A3 1.137371303 5.30131923 3.016021767 0.002857297 0.014472179 -2.324912361

AKR1B10 1.107523648 3.530812865 4.833829072 2.49E-06 3.49E-05 4.260737996

VSIG2 1.09118096 4.106009751 3.610986365 0.00037655 0.002691205 -0.465114802

ZG16 1.080277182 3.118335769 2.90623953 0.004025694 0.019221923 -2.634652682

LRRC26 1.039949763 2.575119549 3.958346233 0.000101404 0.00087468 0.75770679

DUOX2 1.018044403 4.120005897 3.709873832 0.000261666 0.001975744 -0.12705598

AGR3 1.017435071 5.170452774 4.036479865 7.45E-05 0.000670899 1.046220689

CCN2 -1.003219332 7.372540396 -8.050344129 4.86E-14 5.66E-12 21.39769215

EMILIN1 -1.011101328 6.284964449 -8.651185147 1.01E-15 2.05E-13 25.17011973

HSPB7 -1.012532161 2.212644149 -6.125713618 4.02E-09 1.17E-07 10.42401933

CLU -1.013038445 4.548273568 -5.738698415 3.08E-08 7.30E-07 8.463185105

CRYAB -1.01472679 3.480198557 -8.106841685 3.39E-14 4.19E-12 21.74633532

SYNM -1.016856442 2.629504343 -4.700605895 4.53E-06 5.84E-05 3.690374499

SERPINE1 -1.017286894 5.257358518 -6.157284673 3.39E-09 9.99E-08 10.58801248

NTM -1.018408569 2.502858066 -8.789625114 4.04E-16 9.64E-14 26.05852781

FAP -1.021196147 2.792735199 -7.409718084 2.57E-12 1.73E-10 17.54116366

MEDAG -1.022739318 2.620419811 -7.450125998 2.01E-12 1.37E-10 17.77891612

PRRX1 -1.024067446 3.074295882 -7.329663459 4.17E-12 2.60E-10 17.07243365

SERPING1 -1.026665239 6.576918433 -7.534999086 1.20E-12 8.66E-11 18.28079041

CPXM2 -1.03024508 2.634627512 -6.737719574 1.35E-10 5.86E-09 13.70575485

ADAMTS2 -1.035090379 4.505921487 -8.112564332 3.27E-14 4.09E-12 21.78172273

PTGIS -1.035249581 2.500816167 -6.499489089 5.18E-10 1.92E-08 12.4030479

CCL19 -1.035649145 3.218194047 -4.634273082 6.08E-06 7.48E-05 3.411244274

LTBP2 -1.035948831 4.173232475 -8.054050457 4.74E-14 5.57E-12 21.42052324

FBN1 -1.036160752 4.309481447 -7.760563217 2.99E-13 2.71E-11 19.63066327

FLNA -1.037429225 7.803240439 -8.259404318 1.28E-14 1.82E-12 22.69428444

COL8A1 -1.044958138 3.86251473 -6.994971037 3.04E-11 1.52E-09 15.14682231

MMP11 -1.047693905 6.229478337 -5.881305477 1.47E-08 3.74E-07 9.174908744

MYL9 -1.054778059 7.612401756 -7.628181093 6.78E-13 5.31E-11 18.8356369

COL16A1 -1.06147637 3.983327398 -9.665545853 1.09E-18 8.30E-16 31.82569396

CCN1 -1.061856046 6.362074447 -7.074963946 1.90E-11 1.01E-09 15.60194432

GGT5 -1.066521961 3.711343109 -10.45680579 4.30E-21 9.53E-18 37.22026586

ACTA2 -1.067534215 7.606700412 -8.380114147 5.88E-15 9.13E-13 23.45086833

HTRA3 -1.075089245 5.822476322 -8.28150619 1.11E-14 1.61E-12 22.83238713

VCAN -1.075700772 4.569508722 -7.64252839 6.21E-13 4.91E-11 18.92141936

ISLR -1.084490863 6.397198362 -8.593936062 1.46E-15 2.80E-13 24.80474882

OLFML2B -1.086513461 4.830031311 -9.856981953 2.89E-19 2.78E-16 33.11636043

UCHL1 -1.090478036 2.612678572 -7.622877941 7.01E-13 5.46E-11 18.80395302

TNC -1.091148436 5.20945824 -6.55539291 3.78E-10 1.45E-08 12.70592532

COL6A2 -1.09887965 8.046799604 -10.28680932 1.43E-20 2.22E-17 36.04852406

INHBA -1.099837029 4.047531282 -7.496607113 1.52E-12 1.07E-10 18.05335309

PLPP4 -1.102455971 2.523823543 -7.938447584 9.84E-14 1.05E-11 20.71111321

CTHRC1 -1.103643713 5.871108633 -7.220600782 8.01E-12 4.76E-10 16.43884382

MMP9 -1.107224393 5.884476374 -5.796409512 2.29E-08 5.56E-07 8.749664959

GFPT2 -1.108597344 2.512915121 -8.852842451 2.66E-16 6.65E-14 26.46645369

MRC2 -1.109444546 4.903775187 -10.17494311 3.14E-20 4.06E-17 35.28106897

DPYSL3 -1.11032554 5.135890592 -8.40492367 5.01E-15 8.18E-13 23.60707026

TNS1 -1.135064757 4.130803011 -7.776336365 2.71E-13 2.47E-11 19.72591021

AOC3 -1.136443395 3.971454603 -7.592735325 8.43E-13 6.34E-11 18.62410825

COL5A2 -1.137460042 6.360069451 -8.431485278 4.22E-15 7.27E-13 23.77456487

ASPN -1.144401389 4.336653003 -5.561239362 7.61E-08 1.63E-06 7.595647748

SULF1 -1.146937966 6.073464804 -7.038324747 2.36E-11 1.23E-09 15.39307892

KRT17 -1.160470146 4.185222645 -4.368202122 1.92E-05 0.000206166 2.324699093

COL6A3 -1.168835332 6.29398227 -8.699455905 7.32E-16 1.53E-13 25.47911148

MFAP5 -1.170285957 2.565514141 -7.213529395 8.36E-12 4.93E-10 16.39796432

HSPB6 -1.170655871 3.501015203 -5.29343239 2.86E-07 5.22E-06 6.325617478

CCDC80 -1.178921273 3.461938825 -7.746293997 3.27E-13 2.91E-11 19.54459259

LMOD1 -1.179917357 3.996256412 -6.562869925 3.63E-10 1.40E-08 12.74656695

APOE -1.180040318 7.218953277 -5.840567999 1.82E-08 4.55E-07 8.970290539

KRT6B -1.180900913 3.000257378 -4.394100925 1.72E-05 0.000187524 2.42810521

MXRA5 -1.180937432 4.855079649 -8.167006971 2.31E-14 3.07E-12 22.11905075

COL5A1 -1.181424258 6.31400317 -9.978153747 1.24E-19 1.48E-16 33.93831117

COL3A1 -1.191894418 9.684862233 -8.929874323 1.59E-16 4.33E-14 26.96537869

MGP -1.19499483 5.800711448 -6.202766898 2.65E-09 8.02E-08 10.82531

CYP1B1 -1.202833086 1.788210309 -7.443681905 2.09E-12 1.42E-10 17.74094859

SSC5D -1.206925559 2.715670719 -9.66909506 1.06E-18 8.30E-16 31.84953197

TAGLN -1.214432435 7.331785427 -7.740626855 3.39E-13 2.95E-11 19.51043391

C3 -1.220235517 6.034771188 -6.365875538 1.09E-09 3.68E-08 11.68630974

CCL21 -1.224127211 5.402415651 -5.176268278 5.03E-07 8.73E-06 5.785216733

AKAP12 -1.23487849 3.212626271 -7.473045598 1.75E-12 1.20E-10 17.91411297

FBLN2 -1.241419299 4.904575116 -8.554702727 1.89E-15 3.58E-13 24.55504962

ISM1 -1.245517289 1.926414189 -9.660612534 1.12E-18 8.30E-16 31.79256546

SPOCK1 -1.246961614 3.169656099 -8.146056832 2.64E-14 3.39E-12 21.98910038

ADAM12 -1.252863914 2.942564835 -8.605816936 1.35E-15 2.66E-13 24.8804758

C7 -1.260405152 2.193943048 -6.469516633 6.12E-10 2.22E-08 12.24138542

TGFB3 -1.268747471 2.957018186 -9.860969408 2.81E-19 2.78E-16 33.14334813

MYH11 -1.27118008 4.639257127 -4.928100089 1.62E-06 2.40E-05 4.67211023

CCL18 -1.275008953 5.017489023 -4.75123744 3.62E-06 4.82E-05 3.90561432

DES -1.279223871 6.081854889 -3.578559853 0.00042358 0.002967164 -0.574203718

CALB2 -1.283260496 2.270481974 -7.210786622 8.50E-12 4.99E-10 16.38211506

POSTN -1.297899888 6.432852284 -6.604347162 2.87E-10 1.15E-08 12.97258216

COL1A2 -1.298579606 8.959832942 -10.35561979 8.80E-21 1.71E-17 36.52203494

CNN1 -1.299243565 5.179339705 -6.325020751 1.36E-09 4.52E-08 11.46919539

SPP1 -1.304548702 6.940835139 -5.130651704 6.26E-07 1.05E-05 5.577376051

PLIN4 -1.324393453 1.527588518 -6.672267313 1.95E-10 8.19E-09 13.34473807

CILP -1.338810869 1.653332877 -7.733878975 3.53E-13 3.04E-11 19.46977968

SFRP4 -1.359215438 4.99159417 -6.380046698 1.01E-09 3.44E-08 11.7618443

PRELP -1.362056867 2.993226579 -7.089094791 1.75E-11 9.38E-10 15.6826807

L1CAM -1.372018193 2.126217357 -7.020159212 2.62E-11 1.33E-09 15.28977829

FNDC1 -1.372823913 4.1054109 -8.517517356 2.41E-15 4.35E-13 24.31891033

COL11A1 -1.374573669 3.911742905 -7.964416709 8.36E-14 8.94E-12 20.86998547

BGN -1.374917376 8.522088296 -11.25565541 1.40E-23 1.09E-19 42.80529559

MARCO -1.386795037 2.592778282 -6.161608931 3.31E-09 9.81E-08 10.61052081

THBS4 -1.40299384 2.270630133 -6.204114266 2.63E-09 7.98E-08 10.83235843

AEBP1 -1.405948493 7.262967425 -10.79106526 3.98E-22 1.03E-18 39.54228618

GAS1 -1.425924887 2.871319449 -9.238407088 2.01E-17 8.41E-15 28.98340114

THBS2 -1.430140252 6.105038243 -9.173802845 3.10E-17 1.09E-14 28.55830508

COL1A1 -1.466172263 9.786141655 -11.57352811 1.39E-24 2.16E-20 45.0586515

FABP4 -1.575367234 1.868539559 -6.567237129 3.54E-10 1.38E-08 12.77031946

FN1 -1.62446827 7.61281942 -10.99252467 9.38E-23 3.64E-19 40.95245208

COMP -1.654884695 4.0181259 -7.314240926 4.58E-12 2.80E-10 16.98248653

COL10A1 -1.711152531 4.230772656 -7.853165504 1.68E-13 1.61E-11 20.19140713

SFRP2 -2.211728836 5.992511884 -8.858868254 2.55E-16 6.49E-14 26.50540887
