# Supplementary material for: Identification and development of a novel invasion-related gene signature for prognosis prediction in colon adenocarcinoma
Source: Cancer Cell Int. 2021 Feb 12;21:101. doi: 10.1186/s12935-021-01795-1 (PMC7881672; doi:10.1186/s12935-021-01795-1)
Supplement: Supplementary file 5 — Additional file 5: Table S5. Differentially expressed genes between C2 ~ C3 cluster. [file 12935_2021_1795_MOESM5_ESM.docx]

logFC AveExpr t P.Value adj.P.Val B

REG3A 1.294950554 4.478998097 3.43543542 0.000679191 0.003822051 -1.294723834

DEFA6 1.257957009 4.350538612 3.510077904 0.000520163 0.003027096 -1.047018961

TYROBP -1.002097589 5.816977443 -7.338889368 2.23E-12 5.38E-11 17.5098644

GGT5 -1.002645826 2.812062121 -13.18247504 2.46E-31 1.41E-28 60.45177387

HTRA1 -1.003878258 5.732021834 -10.83522212 3.75E-23 4.37E-21 41.87965274

HAPLN3 -1.006337423 3.285494091 -9.658796066 2.74E-19 1.82E-17 33.12182178

DDR2 -1.008223586 2.036840175 -10.44599905 7.48E-22 6.74E-20 38.93182208

CD163 -1.011617182 2.75257093 -7.462948295 1.02E-12 2.56E-11 18.27653541

FPR3 -1.011960333 3.266927108 -7.389773484 1.62E-12 3.94E-11 17.82327128

TGFB3 -1.012909074 1.978414445 -11.18978329 2.36E-24 3.42E-22 44.60338923

VIP -1.013052769 2.18498481 -5.789382448 1.85E-08 2.50E-07 8.726098797

EFEMP2 -1.013100231 3.51475254 -13.86967885 8.23E-34 9.82E-31 66.07432514

HSPB8 -1.018901932 2.26844858 -9.743207096 1.47E-19 1.02E-17 33.7342821

C1QC -1.020762447 6.81183612 -7.071777767 1.17E-11 2.55E-10 15.88912934

IGF2 -1.025083446 4.424567883 -2.82748796 0.005022323 0.021028942 -3.127362433

MAFB -1.025630678 3.166043107 -9.544466642 6.34E-19 3.97E-17 32.29658136

OLR1 -1.025865962 1.706635376 -9.068133965 1.98E-17 9.65E-16 28.91408024

ACKR1 -1.026688088 2.328266926 -7.372371788 1.81E-12 4.38E-11 17.71592496

THBS4 -1.027085272 1.23563504 -7.905529199 5.81E-14 1.76E-12 21.08061025

CAVIN1 -1.02896595 5.860795425 -12.13231902 1.30E-27 3.31E-25 51.99871804

LOX -1.029047862 2.872609814 -10.13456577 7.93E-21 6.37E-19 36.6076739

VSIG4 -1.030517428 3.108660074 -7.251094428 3.87E-12 8.93E-11 16.97259091

ELN -1.031323745 3.87033793 -8.235928068 6.41E-15 2.20E-13 23.24090024

TNFAIP6 -1.03693721 2.299518645 -8.834270204 1.04E-16 4.61E-15 27.28809197

CTSK -1.040610111 6.287421472 -8.732569855 2.11E-16 8.98E-15 26.58850393

SLC24A3 -1.044642589 2.52078458 -10.35724464 1.47E-21 1.30E-19 38.266228

ADAMTS12 -1.044856684 2.319835787 -11.11404917 4.27E-24 5.87E-22 44.01868759

IGFBP6 -1.045144618 3.741650447 -10.15050338 7.03E-21 5.68E-19 36.72582767

PTGIS -1.050931063 1.588514346 -10.084769 1.15E-20 9.03E-19 36.23906454

INHBA -1.052168174 3.110929871 -9.103114852 1.54E-17 7.62E-16 29.15931341

HLA-DPB1 -1.05237798 6.19680426 -7.458441837 1.05E-12 2.62E-11 18.24853436

MFAP5 -1.052474348 1.602936839 -9.496306647 9.02E-19 5.51E-17 31.95046356

THY1 -1.062534526 5.058833888 -11.9048659 8.11E-27 1.77E-24 50.19537797

CXCL10 -1.064643532 4.986004457 -5.288045314 2.46E-07 2.83E-06 6.230083763

CDH11 -1.068712688 2.964354217 -10.73842141 7.92E-23 8.19E-21 41.14224618

RAMP1 -1.06975038 3.832449917 -4.901814014 1.59E-06 1.59E-05 4.434154515

APOC1 -1.076256833 4.559927975 -7.025377505 1.56E-11 3.34E-10 15.61185601

LRRC15 -1.077326214 2.204760139 -9.469438789 1.10E-18 6.57E-17 31.75776159

COL15A1 -1.07916745 4.427913041 -10.21526127 4.31E-21 3.60E-19 37.20679701

MSRB3 -1.079797674 2.533105304 -10.54288392 3.57E-22 3.41E-20 39.6612496

NTM -1.079856506 1.582062321 -12.58787374 3.23E-29 1.25E-26 55.64179659

NNMT -1.083068199 4.439178407 -10.72672488 8.67E-23 8.91E-21 41.05333201

HLA-DQA2 -1.084614779 3.210664752 -5.595571123 5.14E-08 6.54E-07 7.739649919

TIMP3 -1.09293511 1.907899804 -11.71074999 3.83E-26 7.33E-24 48.66530222

GUCY1A1 -1.094032254 2.211944841 -10.30561793 2.18E-21 1.86E-19 37.88023866

PDLIM4 -1.096848435 2.663840815 -11.00495229 1.00E-23 1.27E-21 43.17915247

ITGA5 -1.097598017 4.149941084 -11.87020676 1.07E-26 2.31E-24 49.92156521

COL8A2 -1.100606856 2.056606908 -12.30326981 3.27E-28 9.56E-26 53.36112106

CD248 -1.106519334 4.853516512 -10.97241912 1.29E-23 1.60E-21 42.92943741

FAP -1.110886878 1.855184053 -10.82081252 4.19E-23 4.78E-21 41.76970962

SPHK1 -1.112120176 2.597949093 -10.82868441 3.94E-23 4.56E-21 41.82976349

CALD1 -1.114437766 4.994114417 -9.156655933 1.05E-17 5.40E-16 29.53566055

PLN -1.11607825 2.204349825 -8.180797413 9.30E-15 3.12E-13 22.87660735

ITGB2 -1.117928201 3.932086901 -8.900991021 6.48E-17 2.94E-15 27.74955577

PCOLCE -1.120565395 4.553335606 -12.16337686 1.01E-27 2.66E-25 52.24579797

DPYSL3 -1.124016745 4.159014714 -9.446989883 1.29E-18 7.68E-17 31.59697041

GFPT2 -1.124508275 1.536423428 -13.54249689 1.25E-32 1.08E-29 63.38988931

EHD2 -1.124763419 4.495673491 -12.38573065 1.67E-28 5.53E-26 54.02035538

MXRA5 -1.129231079 3.849679506 -9.169088683 9.63E-18 4.96E-16 29.62322354

PRRX1 -1.13145945 2.125261195 -11.24011978 1.59E-24 2.42E-22 44.99285575

ACTA2 -1.134346358 6.640270682 -11.28131328 1.15E-24 1.78E-22 45.31207612

SPARC -1.139289296 8.390677606 -10.86972873 2.87E-23 3.42E-21 42.14317798

PDGFRB -1.141550082 4.677166699 -12.85298613 3.70E-30 1.69E-27 57.7793116

COL12A1 -1.143099615 4.90973011 -9.532144901 6.94E-19 4.31E-17 32.20794139

FIBIN -1.1448413 3.000634171 -10.16522124 6.29E-21 5.14E-19 36.83501552

CXCL9 -1.148627489 4.162912133 -5.805004952 1.71E-08 2.31E-07 8.806771021

VCAN -1.153084875 3.5905846 -10.17995413 5.63E-21 4.65E-19 36.94438848

CERCAM -1.159703737 3.46051683 -12.39315949 1.58E-28 5.32E-26 54.07980941

SCARF2 -1.163408945 2.516902196 -14.58047988 2.14E-36 6.84E-33 71.94483615

CHRDL1 -1.16676538 1.515752895 -8.61767798 4.70E-16 1.88E-14 25.80379868

SSC5D -1.16937653 1.680390077 -13.81205959 1.33E-33 1.47E-30 65.60066777

AOC3 -1.171312173 2.961025337 -11.0787113 5.63E-24 7.41E-22 43.74639371

TIMP2 -1.179801004 5.869881911 -11.28468328 1.12E-24 1.75E-22 45.33821079

C1S -1.182662744 5.605755253 -9.975469982 2.62E-20 1.94E-18 35.43301607

C1R -1.188081148 5.542835548 -12.25377426 4.88E-28 1.35E-25 52.96606336

MRC2 -1.192170945 3.892665148 -13.20555591 2.04E-31 1.25E-28 60.63959566

MXRA8 -1.201453381 4.648438821 -13.53346392 1.35E-32 1.10E-29 63.31596076

MRGPRF -1.201625301 2.616970637 -12.35007465 2.24E-28 7.23E-26 53.7351423

EFEMP1 -1.203386412 3.611133176 -9.330018196 3.02E-18 1.68E-16 30.76239044

COL5A2 -1.211153963 5.329066097 -10.9377869 1.69E-23 2.06E-21 42.66393743

MGP -1.211319167 4.748536918 -9.285750981 4.16E-18 2.25E-16 30.44798165

PLPP4 -1.211499921 1.505474319 -12.54125215 4.73E-29 1.75E-26 55.2671685

ADAM12 -1.213388348 1.868131206 -12.98068471 1.30E-30 6.49E-28 58.81306816

DCN -1.214840605 4.529106874 -8.750105334 1.87E-16 7.97E-15 26.708798

FCGR3A -1.217752759 4.011063471 -7.764314586 1.47E-13 4.15E-12 20.17446676

OLFML2B -1.218489527 3.813981245 -13.61204349 7.03E-33 6.41E-30 63.95942304

CHRDL2 -1.222870291 1.779071351 -8.278448793 4.81E-15 1.69E-13 23.52289384

COL5A1 -1.22302007 5.260869617 -11.69002524 4.52E-26 8.34E-24 48.50245175

FLNA -1.225433868 6.801659448 -11.65807101 5.83E-26 1.06E-23 48.25155892

TNS1 -1.233262874 3.089467415 -11.59561484 9.57E-26 1.69E-23 47.76187131

EMILIN1 -1.235192566 5.288084977 -12.18286439 8.65E-28 2.35E-25 52.40093093

MAB21L2 -1.24172598 2.437205651 -11.00137916 1.03E-23 1.30E-21 43.15171174

TREM2 -1.24280361 3.130074439 -9.048852803 2.27E-17 1.09E-15 28.77913164

FBN1 -1.246986118 3.29743747 -11.78398817 2.13E-26 4.24E-24 49.24158515

LUM -1.249701519 7.137039072 -8.173274578 9.78E-15 3.27E-13 22.8270149

MMP9 -1.252530663 4.843575129 -7.846106795 8.59E-14 2.53E-12 20.69802904

MYL9 -1.255442967 6.589246261 -11.54529403 1.43E-25 2.41E-23 47.36800895

LMOD1 -1.256934771 2.926478976 -10.11006849 9.53E-21 7.62E-19 36.42623192

RAB31 -1.262415808 4.048039158 -11.14013376 3.48E-24 4.90E-22 44.21989829

PRELP -1.277588737 1.846219694 -10.39790228 1.08E-21 9.62E-20 38.57081651

MARCO -1.287633766 1.431611212 -8.963876403 4.16E-17 1.93E-15 28.18627776

COL6A1 -1.29479953 6.596574496 -13.45876897 2.51E-32 1.85E-29 62.70503655

CCDC80 -1.30358867 2.368870971 -12.57901038 3.48E-29 1.31E-26 55.57054492

MMP11 -1.304420824 5.184085454 -9.358507175 2.46E-18 1.38E-16 30.96515251

ANGPTL2 -1.309635998 4.37900715 -12.34265446 2.37E-28 7.50E-26 53.67581869

ITGA11 -1.324940795 2.438995341 -14.41005367 8.95E-36 2.31E-32 70.5330917

MFAP4 -1.334954164 4.830940475 -9.482716246 9.96E-19 5.99E-17 31.85295502

COL6A3 -1.335898373 5.188228688 -11.52111175 1.73E-25 2.88E-23 47.17895149

TAGLN -1.343259944 6.205587158 -11.14727918 3.29E-24 4.69E-22 44.27504852

SERPING1 -1.344892712 5.518711932 -12.0071807 3.57E-27 8.38E-25 51.00520954

GAS1 -1.371145393 1.653466746 -13.9237773 5.24E-34 6.82E-31 66.51937408

ACTG2 -1.385247585 4.853840089 -8.696917642 2.71E-16 1.13E-14 26.3443572

COL6A2 -1.396378976 6.936019666 -14.8686301 1.89E-37 9.78E-34 74.33679598

DPT -1.398569994 2.541068352 -9.538634718 6.62E-19 4.12E-17 32.25462043

ANTXR1 -1.409502314 4.306432901 -10.76496463 6.46E-23 7.00E-21 41.34417213

HTRA3 -1.409790774 4.713615596 -12.70411783 1.25E-29 5.25E-27 56.5775534

FBLN1 -1.415672128 4.396135419 -11.09241532 5.06E-24 6.77E-22 43.85194882

COL8A1 -1.418216351 2.760425408 -11.98608775 4.22E-27 9.78E-25 50.83807203

MYH11 -1.424238585 3.451200354 -8.23245325 6.56E-15 2.25E-13 23.21789489

ADAMTS2 -1.438019413 3.397407867 -13.24072741 1.52E-31 9.84E-29 60.92595324

C3 -1.44111588 4.856828903 -9.036130438 2.49E-17 1.19E-15 28.69017466

IGFBP5 -1.443779359 6.094092377 -10.49361839 5.20E-22 4.80E-20 39.2899696

ASPN -1.459266351 3.177302071 -9.326488289 3.10E-18 1.72E-16 30.73729011

COL3A1 -1.465460865 8.504963473 -12.02697409 3.04E-27 7.49E-25 51.16213574

CTHRC1 -1.472513948 4.719978943 -11.10687494 4.52E-24 6.10E-22 43.9633795

MMP2 -1.477931401 6.346548734 -12.34042184 2.42E-28 7.50E-26 53.65797121

CCL18 -1.488070362 3.7956635 -6.552375633 2.63E-10 4.69E-09 12.86018126

GPNMB -1.502356016 4.247612926 -10.16045524 6.52E-21 5.30E-19 36.79964977

POSTN -1.522544762 5.185156893 -9.614321986 3.80E-19 2.47E-17 32.8002108

TNC -1.533558592 4.031956054 -11.8111163 1.72E-26 3.55E-24 49.45535645

BGN -1.545016652 7.234770579 -13.76900979 1.90E-33 1.97E-30 65.2470233

SPOCK1 -1.547973718 1.927736837 -14.11105734 1.10E-34 2.13E-31 68.06245387

APOE -1.559972425 5.995477879 -8.967288557 4.06E-17 1.89E-15 28.2100234

GREM1 -1.567336126 3.361984645 -10.48696311 5.47E-22 5.02E-20 39.23987179

COL1A2 -1.574371164 7.685610248 -13.39074215 4.40E-32 2.97E-29 62.14928615

CNN1 -1.591243062 3.896318894 -10.76776086 6.32E-23 6.90E-21 41.36545638

COL11A1 -1.595182839 2.599114784 -12.77687002 6.90E-30 3.06E-27 57.16439052

CCL21 -1.610063277 4.137383529 -8.020783534 2.71E-14 8.51E-13 21.82788687

ISLR -1.623531688 5.17651612 -13.1336475 3.68E-31 1.97E-28 60.05469336

SULF1 -1.62801778 4.827620071 -11.74529368 2.91E-26 5.63E-24 48.9369598

AEBP1 -1.685845647 5.89287006 -15.76821908 9.43E-41 7.31E-37 81.83504768

FNDC1 -1.687000146 2.746868838 -14.57692195 2.20E-36 6.84E-33 71.91533977

FBLN2 -1.707712939 3.583692665 -14.07881117 1.44E-34 2.47E-31 67.79650769

COL1A1 -1.728297524 8.37244643 -14.12313043 9.91E-35 2.13E-31 68.16205178

DES -1.817589489 4.691404411 -7.526961762 6.78E-13 1.75E-11 18.6755082

SPP1 -1.848103364 5.525630885 -7.890756797 6.41E-14 1.93E-12 20.98532801

COMP -1.872643227 2.462075208 -11.84454274 1.31E-26 2.76E-24 49.71898673

THBS2 -2.001109606 4.566222067 -13.96003574 3.87E-34 6.01E-31 66.81783692

COL10A1 -2.041990614 2.568232631 -12.3272828 2.69E-28 8.18E-26 53.5529578

SFRP4 -2.118804523 3.419096766 -12.17189749 9.46E-28 2.53E-25 52.31361803

FN1 -2.123510243 5.940716653 -15.94641169 2.08E-41 3.23E-37 83.32412703

SFRP2 -2.591904807 3.867672984 -12.95365267 1.62E-30 7.61E-28 58.59401935
