# Supplementary material for: Identification and development of a novel invasion-related gene signature for prognosis prediction in colon adenocarcinoma
Source: Cancer Cell Int. 2021 Feb 12;21:101. doi: 10.1186/s12935-021-01795-1 (PMC7881672; doi:10.1186/s12935-021-01795-1)
Supplement: Supplementary file 6 — Additional file 6: Table S6. 17 genes related to prognosis based on 983 DEGs [file 12935_2021_1795_MOESM6_ESM.docx]

p.value HR Low 95%CI High 95%CI

CLCA1 0.031239748 0.897555625 0.813488027 0.990310949

ITLN1 0.006203957 0.856686458 0.766853197 0.957043265

SPINK4 0.047880924 0.902393691 0.815095095 0.999042172

SLC26A3 0.031814895 0.886339624 0.793887742 0.989557953

AKR1B10 0.007875422 0.800902097 0.679928966 0.943398799

ZG16 0.041994326 0.875793815 0.770705951 0.995210696

FABP4 0.024505462 1.169217456 1.020301785 1.339867753

CLCA4 0.022123496 0.830670753 0.708625496 0.973735639

FCGBP 0.022102889 0.864329142 0.762870598 0.979281241

HOXD9 0.006709146 1.342069626 1.084915861 1.660175637

INHBB 0.034815828 1.214487462 1.013956431 1.454677685

TSPAN11 0.029144956 0.671445516 0.469451173 0.960353509

GPRC5B 0.047468169 1.444404889 1.004114677 2.077756187

TIMP1 0.036778061 1.559312942 1.027660517 2.366011741

SNAI1 0.024073143 1.549466969 1.059139337 2.266791351

CXCL13 0.039226153 0.817181237 0.674478006 0.99007702

HOMER3 0.042238216 1.409798415 1.01211211 1.963746456
